# Elucidating fish oil-induced milk fat depression in dairy sheep: Milk somatic cell transcriptome analysis

**DOI:** 10.1038/srep45905

**Published:** 2017-04-05

**Authors:** Aroa Suárez-Vega, Pablo G. Toral, Beatriz Gutiérrez-Gil, Gonzalo Hervás, Juan José Arranz, Pilar Frutos

**Affiliations:** 1 Departamento de Producción Animal, Facultad de Veterinaria, Universidad de León, Campus de Vegazana s/n, León 24071, Spain; 2 Instituto de Ganadería de Montaña (CSIC-ULE), Finca Marzanas s/n, Grulleros 24346, León, Spain

## Abstract

In this study, RNA sequencing was used to obtain a comprehensive profile of the transcriptomic changes occurring in the mammary gland of lactating sheep suffering from fish oil-induced milk fat depression (FO-MFD). The milk somatic cell transcriptome analysis of four control and four FO-MFD ewes generated an average of 42 million paired-end reads per sample. In both conditions, less than 220 genes constitute approximately 89% of the total counts. These genes, which are considered as core genes, were mainly involved in *cytoplasmic ribosomal proteins* and *electron transport chain* pathways. In total, 117 genes were upregulated, and 96 genes were downregulated in FO-MFD samples. Functional analysis of the latter indicated a downregulation of genes involved in the *SREBP signaling* pathway (e.g., *ACACA, ACSL*, and *ACSS*) and Gene Ontology terms related to *lipid metabolism* and *lipid biosynthetic* processes. Integrated interpretation of upregulated genes indicated enrichment in genes encoding plasma membrane proteins and proteins regulating protein kinase activity. Overall, our results indicate that FO-MFD is associated with the downregulation of key genes involved in the mammary lipogenesis process. In addition, the results also suggest that this syndrome may be related to upregulation of other genes implicated in signal transduction and codification of transcription factors.

Sheep milk has a higher content of milk solids than either cow or goat milk, which makes it particularly suitable for the production of cultured dairy products, such as high-quality cheeses. The major proportion of sheep milk fatty acids (FAs) includes saturated FAs (SFA) (>60%), whereas the concentrations of monounsaturated and polyunsaturated FAs (MUFA and PUFA) are much lower (approx. 19–25% and 3–6%, respectively)^1^,^2^. Given the beneficial effects of MUFA and PUFA in human health^3^, different strategies have been developed to modulate the milk FA profile in dairy livestock.

Thus, in dairy sheep, genetic studies have been performed to assess the possible influence of candidate genes implicated in FA desaturation and to identify quantitative trait loci (QTLs) in relation to milk FA composition^4^,^5^. In the nutrition field, different feeding strategies have been applied with the aim of increasing milk PUFA rates^6^,^7^,^8^,^9^,^10^.

Diet supplementation with marine lipids (namely, fish oil, FO) is an effective tool to modify the milk FA profile by increasing the content of certain bioactive lipids, such as n-3 PUFA and conjugated linoleic acid (CLA)^7^,^8^,^10^. However, this type of supplementation has also been associated with milk fat depression (MFD) syndrome^11^. In general, this syndrome is characterized by a severe reduction of milk fat content with no changes in milk yield or other milk components^12^,^13^. Given that most milk from small ruminants is used for the manufacture of cheeses, MFD hinders the application of this feeding strategy (i.e., the addition of FO to the sheep diet) under practical farm conditions.

The molecular mechanisms underlying marine lipid-induced MFD are not well characterized in ruminants in general and particularly in sheep^11^,^13^,^14^, and MFD nutrigenomic research mainly relies on the evaluation of candidate genes associated with lipid metabolism^2^,^14^. However, molecular mechanisms of FO-MFD appear to be complex, and information at the omic level may be very useful to understand these mechanisms. In this regard, next generation RNA sequencing technology (RNA-Seq) offers valuable methodology to unravel previously challenging transcriptomic complexities as it allows complete characterization of the transcriptome and provides the opportunity to quantify transcripts and identify differential regulation between two or more conditions^15^,^16^.

Hence, the goal of this study was to elucidate molecular mechanisms underlying FO-induced MFD in dairy sheep using RNA-Seq. To that aim, we compared gene expression profiles of milk somatic cell (MSCs) transcriptomes from control and FO-MFD lactating ewes.

## Results

### Sequencing and alignment of the ovine MSCs transcriptome

For the four control and four FO-MFD samples sequenced, a total of 42 million paired-end reads per sample were generated on average (mean ± SD = 41,512,563.0 ± 6,396,467.09), with the exception of one control sample that had to be re-sequenced. For this control sample, we acquired two technical replicates of 17,686,309 and 24,006,681 million paired-end reads. Approximately 88.56% of total reads uniquely mapped to the *Ovis aries* genome (Oar_v3.1).

### Power calculations

The power results obtained using the online power analysis tool Scotty (http://scotty.genetics.utah.edu/) are described in Supplementary File S1. According to the parameters indicated in the Material and Methods section, the least expensive experiment to accomplish a differential expression analysis with sufficient power was sequencing five replicates to a depth of 10 million reads aligned to genes per replicate. The most powerful experiment that fits our settings was sequencing 10 replicates to a depth of 40 million reads aligned to genes per replicate. Based on these calculations, the size of our experiment provides adequate power to detect an acceptable proportion of genes differentially expressed under our experimental conditions.

### Gene expression level

**Figure 1:**
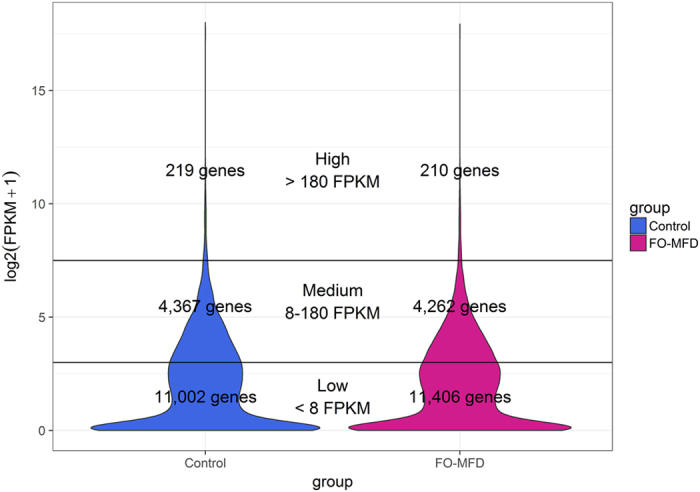
Violin plots displaying the expression intensity distribution of the genes within the milk somatic cell transcriptome. The violin plots are filled in blue (left) and pink (right) for control and fish oil-induced milk fat depression (FO-MFD) samples, respectively. Two transversal black lines separate the three defined groups of expression intensity: low expressed genes (<8 FPKM), middle expressed genes (8–180 FPKM) and highly expressed genes (>180 FPKM).

Gene expression was normalized using the Fragments per Kilobase per Million Mapped Reads (FPKM) method. The total number of expressed genes (genes with at least 0.01 FPKM in one sample^15^) was 17,134, with an average of 14,195.5 ± 644.51 genes per control sample and 14,422.8 ± 380.09 per FO-MFD sample. According to the FPKM value, expressed genes were classified into low expressed genes (<8 FPKM), middle expressed genes (8–180 FPKM) and highly expressed genes (>180 FKM) (Fig. 1). A total of 219 and 210 genes were identified as highly expressed in the control and FO-MFD samples, respectively. These highly expressed genes were considered ‘core’ genes of the MSCs transcriptome, as they constitute approximately 89% of total FPKM. Among the core genes identified in the two analyzed conditions (control and FO-MFD), a total of 197 genes were identified as highly expressed for both conditions, whereas 22 genes were assigned to the group of highly expressed only in the control and 13 in the FO-MFD samples.

**Figure 2:**
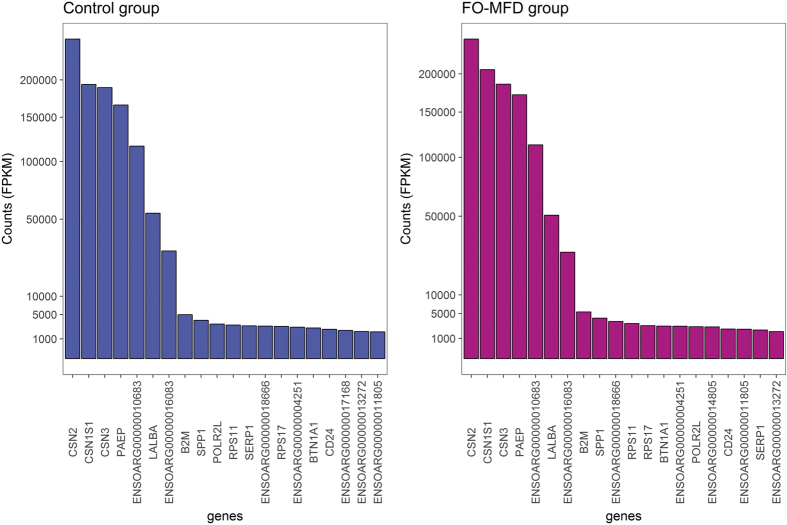
Top 20 expressed genes in the milk somatic cell transcriptome in control and fish oil-induced milk fat depression (FO-MFD) ewes. Graphical representation of the expression levels for the top 20 highly expressed genes in control (blue, left) and FO-MFD (pink, right) ewes are plotted. For each bar graph, *Ovis aries* gene names (Ovine genome assembly Oar_v3.1) are indicated in the X-axis, and FPKM values are represented in the Y-axis.

The top 20 genes expressed in the MSCs in the control and FO-MFD supplemented sheep are presented in Fig. 2. These genes constituted on average 80.92% and 81.54% of total FPKM for the control and the FO-MFD samples, respectively. The profile of the top 20 genes was very similar for both conditions, with a considerably increased expression level (involving greater than 20,000 FPKM) for the genes codifying for caseins (*CSN1S1, ENSOARG00000010683*/*CSN1S2, CSN2, CSN3*), whey proteins (*PAEP* and *LALBA*) and glycosylation-dependent cell adhesion molecule-1 (*ENSOARG00000016083*/*GlyCAM-1*) compared with the remaining genes with <10,000 FPKM (Fig. 2).

To better categorize the core genes, WikiPathways functional enrichment analysis was performed with WebGestalt (Supplementary File S2)^17^. The top three pathways highlighted by the analysis based on the genes identified as highly expressed in both conditions were *cytoplasmic ribosomal proteins* (31 genes; p_adj_ = 7.75e-57), *electron transport chain* (9 genes; p_adj_ = 4.19e-10) and *oxidative stress* (3 genes; p_adj_ = 0.0025). The 22 genes highly expressed in the control group were enriched in the *SREBP signaling pathway* (2 genes; p_adj_ = 0.0005), and the 13 highly expressed in the FO-MFD sheep in the *selenium metabolism and selenoproteins pathway* (2 genes; p_adj_ = 3.53e-05).

### Comparison of transcriptome profiles between control and FO-MFD conditions

**Figure 3:**
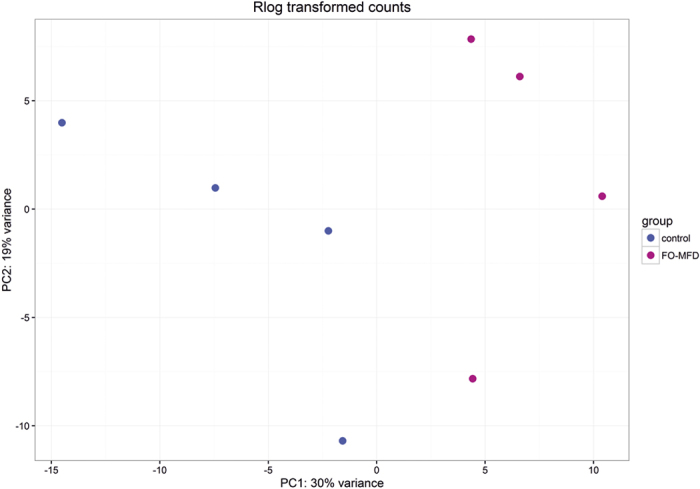
Principal Component Analysis (PCA) plot of milk somatic cell transcriptomes from control and fish oil-induced milk fat depression (FO-MFD) ewes. The control (blue) and FO-MFD (pink) samples are plotted along the first two principal component axes (PC1 and PC2).

Principal component analysis (PCA) performed on the basis of the rlog transformed gene counts revealed a clear difference between the transcriptome profiles of control and FO-MFD ewes (Fig. 3). In the first principal component (PC1), which accumulated 30% of the variance, the control and the FO-MFD gene expression profiles were grouped in distinct clusters. The control samples, which seemed more heterogeneous, fell in the negative direction of the PC1 axis, whereas the FO-MFD samples fall in the positive direction.

PC2 accumulates 19% of the variance. In this component, there were two samples, one control and one FO-MFD sample, which did not cluster with the others likely due to individual variability in the animals.

### Differential genetic expression in MSCs between control and FO-MFD conditions

There were 213 differentially expressed genes (DEGs) in the MSC transcriptomes between the control and the FO-MFD sheep. Of these DEGs, 117 genes were upregulated in FO-MFD, and 96 were downregulated in FO-MFD (Supplementary File S3). We performed Gene Ontology (GO) and WikiPathways functional enrichment analyses in the up- and downregulated differentially expressed genes.

Among the genes upregulated in the FO-MFD condition, the significant (*p*_*adj*_ < 0.05 and a minimum of 5 genes) GO enrichment terms were categorized into 18 functional groups (Supplementary File S4) across the three GO established classes: “biological processes” (3 terms), “molecular function” (6 terms) and “cellular component” (9 terms). The three highest enriched GO terms in each of the categories were: *regulation of protein kinase activity (p*_*adj*_ = 0.0397), *growth factor binding (p*_*adj* _=_ _0.0330) and *plasma membrane part (p*_*adj*_ = 0.0109) in the biological process, molecular function and cellular component categories, respectively. There were two enriched pathways among the FO-MFD upregulated genes in the WikiPathways analysis (Supplementary File S4?): *TGF beta signaling pathway (p*_*adj*_ = 3.57e-05; 5 genes) and *adipogenesis (p*_*adj*_ = 3.57e-05; 5 genes).

Genes downregulated in the FO-MFD condition were significantly clustered (*p*_*adj*_ < 0.05 and a minimum of 5 genes) in 82 GO functional enrichment terms (Supplementary File S5). Forty terms were significantly enriched in relation to the “biological processes” category, and 22 and 20 GO terms belonged to the “molecular function” and “cellular component” categories, respectively. The highest significantly enriched ‘biological processes’ terms (*p*_*adj*_ < 1e-05) were associated with *lipid metabolisms* and *lipid biosynthetic processes* (Supplementary File S5). The highest enriched GO terms in the “molecular function” and “cellular component” categories were *ligase activity, forming carbon-sulfur bonds* (p_adj_ = 1.81e-07) and *cytoplasm* (p_adj_ = 1.54e-07). The WikiPathways analysis of the 96 downregulated genes for the FO-MFD samples identified one significantly enriched pathway (Supplementary File S5): *SREBP signaling (p*_*adj*_ = 8.99e-09; 6 genes).

### Quantitative Real-Time PCR (qRT-PCR) validation

For the validation of the RNA-Seq results, qRT-PCR analysis was performed in a subset of 12 genes: four upregulated (*APP, ATF3, KLF6* and *PPT1*), four downregulated (*ACACA, ACSS1, ACSS2* and *LPIN1*), and four non-differentially expressed genes (*GPAM, GPAT4, SCD* and *SREBF1*). Comparison between fold changes estimated with DESeq2 in the RNA-Seq data and mRNA abundances determined by qRT-PCR exhibited a Pearson correlation coefficient of r = 0.981 (Supplementary File S6). Hence, these 12 genes exhibited similar patterns of mRNA abundance when expression is assessed using these two different technologies.

## Discussion

Diet supplementation with FO is an effective strategy to modulate ewe milk fatty acid composition towards a healthier profile^8^,^10^. However, the associated MFD constrains the dairy sheep industry due to the concomitant reduction of milk fat concentration. During recent years, RNA-Seq has been progressively used in livestock functional genomics as a revolutionary technology to attempt comprehensive transcriptome analyses that can be useful to detect genetic variants or identify potential biomarkers associated with phenotypes of economic interest^18^. In this context, the transcriptome analysis presented herein provides a thorough view of DEGs, biological processes and pathways associated with FO-MFD. This knowledge may clarify the molecular mechanisms underlying this syndrome in dairy sheep.

RNA-Seq stranded sequencing was performed in nine RNA samples extracted from MSCs from four FO-MFD and four control lactating ewes. The number of animals used in the experiment was limited due to ethical considerations that recommend following the principle of animal reduction. However, according to the power estimations, the use of four replicates per condition sequenced to a minimum depth per sample of 30 million paired-end reads is adequate for evaluation of the DEGs between the control and FO-MFD ewes (Supplementary File S1).

MSCs are a representative source of lactating sheep mammary gland^19^. The top-20 expressed genes in the MSCs in this study were consistent with other studies performed on lactating mammary gland, with genes codifying for caseins, whey proteins and *GLYCAM1* at the top of the list^20^,^21^,^22^. Moreover, the distribution of genes according to their expression intensity (Fig. 1) was also very similar to that reported in the previously mentioned studies^20^,^21^,^22^, with a small number of genes contributing to a large fraction of the total RNA. This distribution highlights the low complexity of the mammary gland transcriptome during lactation compared with other developmental stages of this organ^22^.

Results from this study demonstrated that less than 220 genes included within the high gene expression group (>180 FPKM) constitute approximately 89% of total FPKM. These highly expressed genes were considered as ‘core’ genes and were divided into the three following groups: (i) genes identified as highly expressed genes in the two analyzed conditions (197), (ii) genes that were only highly expressed in the FO-MFD (13) and (iii) genes that were only highly expressed in the control condition (22). The most abundant transcripts in MSCs identified in both the control and the FO-MFD sheep were those that codify for major milk proteins. According to the additional functional enrichment analysis performed to better understand the biological role of the other core genes, common genes identified in the two conditions as highly expressed genes were involved in *cytoplasmic ribosomal proteins* and *electron transport chain* pathways. These genes were implicated in the transduction processes and the generation of the required energy (ATP) for the massive protein production that occurs in the lactating mammary gland. However, although core genes from both FO-MFD and control sheep were associated with protein synthesis, some biological pathways were uniquely enriched within one of the specific conditions. In the FO-MFD animals, the *selenium metabolism and selenoproteins pathway* were enriched. Although a report in dairy cows^23^ suggests that dietary supplementation with selenium may contribute to alleviate MFD, results are not conclusive, and caution should be taken before relating the two core genes (*SELENBP1* and *FOS*) implicated in this pathway and MFD mechanisms. The *SREBP signaling* pathway, a term enriched among core genes in control animals, is related to fatty acid synthesis. Two genes within this pathway are involved in transcription regulation (*INSIG1*) and intracellular FA transport (*DBI*)^24^.

A total of 213 genes exhibit significantly different expression levels between FO-MFD and control. Interestingly, among the downregulated DEGs in FO-MFD animals, the *SREBP signaling* pathway was functionally enriched. This observation and the fact that this pathway was also enriched for the core genes of control animals suggest that the lack of stimulation of this *SREBP signaling* pathway may underlie the MFD mechanisms. The sterol regulatory element-binding protein (SREBP) pathway is a route implicated in lipogenesis^25^,^26^, in which the sterol regulatory element-binding transcription factors (SREBFs) and their regulatory SREBF cleavage-activating protein (SCAP) are crucial in the regulation of mRNA expression of enzymes related to *de novo* synthesis of fatty acids and cholesterol^25^,^27^. The *SREBP signaling* pathway has also been proposed to participate in dietary regulation of lipogenic gene expression during lactation, being critical in MFD^14^,^28^. In this analysis, the genes encoding neither SREBFs nor the SCAP protein were identified as differentially expressed. This finding would support the lack of a clear downregulation of the *SREBF1* gene observed via qRT-PCR analysis of candidate genes in the same samples^29^ but contrasts with other studies in sheep and cattle reporting a decreased expression of the *SREBF1* gene associated with MFD^14^,^30^. In any event, our analysis revealed that several lipogenic and cholesterogenic enzymes regulated by SREBFs (*LSS, HMGCS1, ACSS1, MVD, ACACA* and *FDPS*) were downregulated in FO-MFD ewes. This downregulation of the SREBFs target genes without changes in the expression levels of the transcription factors themselves may also suggest an effect at the post-transcriptional instead of at the mRNA level.

**Figure 4:**
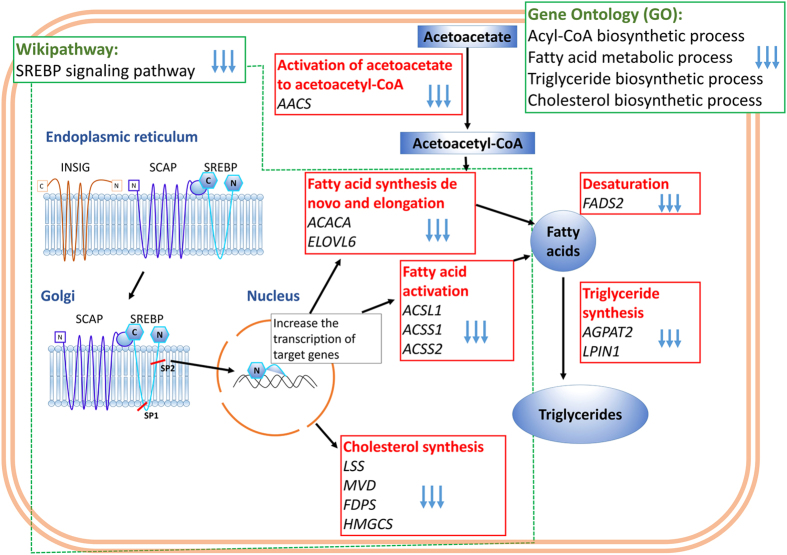
Interrelationships among proteins codified by genes within major downregulated pathways and Gene Ontology (GO) terms in fish oil-induced milk fat depression (FO-MFD) samples. Principal WikiPathways (top-left) and GO-term results (top-right) from the functional enrichment analysis of downregulated genes in the FO-MFD samples are indicated in green boxes. Blue arrows denote downregulation. Red boxes contain significantly downregulated genes (black) and their function (red). Blue boxes indicate potential products of the proteins codified by the downregulated genes.

Consistent with the WikiPathways analysis, the two highest enriched GO terms among the downregulated genes in FO-MFD animals were also related to lipid metabolism. More precisely, the highest enriched terms were *lipid biosynthetic process* and *lipid metabolic process*. However, these GO terms were too general to allow us to draw conclusions regarding metabolic pathways downregulated in the MFD. This finding adds to the complexity of the milk fat synthesis in the mammary gland, with the implication of a complex network of genes^24^. In this regard, in the hierarchical distribution of GO terms, which encompass the most general to the most specific terms, we tried to identify those specific GO terms that are related to more precise biological processes within the mammary gland lipid metabolism. Hence, we identified the following GO terms: *acyl-CoA biosynthetic process (ACSL1, ACSS1, ACSS2, ELOVL6* and *ACACA*), *triglyceride biosynthetic process (ACSL1, PNPLA3, AGPAT2, ELOVL6, ACACA* and *LPIN1*), *fatty acid metabolic process (FADS2, ACSL1, ACSS1, AACS, ELOVL6, ACACA* and *LPIN1*) and *cholesterol biosynthetic process (LSS, HMGCS1, G6PD, MVD* and *FDPS*). This finding would support the influence of these downregulated biological processes in mammary gland milk fat synthesis and their relation with MFD. Milk fat consists essentially of triglycerides (TAGs) arising from uptake from blood circulating FAs or *de novo* synthesis^31^,^32^ plus a small amount of cholesterol, phospholipids and free FAs^32^. Our results revealed the downregulation of key genes involved in both *de novo* synthesis and uptake processes. These genes were associated with the activation of acetoacetate to acetoacetyl-CoA (*AACS*), activation of FA with CoA (*ACSL1, ACSS1, ACSS2*), *de novo* synthesis (*ACACA, ELOVL6*), desaturation (*FADS2*) and synthesis of TAGs (*AGPAT2, LPIN1*), which suggests that the reduction of milk fat content was related to their downregulation. A custom scheme summarizing the principal findings about the downregulated genes in FO-MFD lactating mammary gland is presented in Fig. 4. Some of these genes, specifically *ACSS1, ACACA*, and *LPIN1*, were confirmed by qRT-PCR in a previous study on candidate genes using not only the same RNA samples but also additional samples of mammary gland that were biopsied from the same animals^29^. That study also identified a significant difference in the expression levels of two genes involved in lipid metabolism (*FASN* and *INSIG1*) that in our RNA-Seq analysis showed a *p-value* < 0.05 but did not pass the multiple-adjustment threshold *p*_*adj*_ < 0.05. Furthermore, although the cholesterol content was not analyzed herein, several genes related to its synthesis were downregulated (*LSS, HMGCS1, G6PD, MVD, FDPS*), which might allow one to presume a reduction in its content in milk from FO-MFD animals. Although this hypothesis might be supported by a report in cattle suggesting that plant oils inducing MFD would reduce milk cholesterol levels^33^, caution should be taken when comparing different ruminant species in terms of MFD because ewes, in contrast to cows, are not sensitive to the negative effects of plant oils on milk fat concentration^2^,^6^,^13^.

The WikiPathways analysis of the upregulated genes in the FO-MFD ewes identified as enriched two pathways: *adipogenesis* and *TGF-beta signaling*. The expression of genes involved in the *adipogenesis* pathway in the analyzed MSCs could be due to the close functional interrelation between the mammary epithelial-stroma and the surrounding adipocytes, which is known as mammary fat pad^34^. Regarding the *TGF-beta signaling pathway*, which is implicated in immune response modulation^35^, a report in mice demonstrated that diet supplementation with FO was associated with increased expression of *TGF-beta*^36^, which would merit further research.

Among the results from the GO functional enrichment analysis in the FO-MFD upregulated genes, the large number of genes clustered within GO terms related to the plasma membrane is worthy of mention as it may suggest that dietary FO has an effect on the plasma membrane composition in MSCs. In addition, n-3 PUFA seems to influence lipid raft formation and function in immune cells^37^. Lipid rafts are combinations of cholesterol, glycosphingolipids and protein receptors organized in glycolipoprotein microdomains that are involved in signal transduction through the activation of signaling pathways involving kinases and phosphatases^38^. Within our GO term results, we have functionally enriched the biological process *regulation of protein kinase activity* and some genes clustered in this category that may be related to FO-MFD, such as *APP, DUPS1, FGFR1* or *ATF3*. The interaction of *APP*, which codifies for amyloid precursor protein, with SREBP1 in the Golgi reticulum has been reported in neurons to inhibit the release of mature SREBP1 (a SREBFs) and its nuclear translocation^39^. Hence, we hypothesize that if this interaction also occurred in mammary epithelial cells, the target enzymes of the *SREBP signaling* pathway would be inhibited without downregulation of *SREBP1* gene mRNA levels, which is consistent with our results. Thus, the upregulation of the *APP* gene in the FO-MFD sheep could be a key point in MFD. The *DUSP1* gene codifies for dual specificity phosphatase 1 and is upregulated by dietary PUFAs^40^. The DUSP1 protein is implicated in the inactivation of the mitogen-activated protein kinases (MAPKs) ERK, p38 and JNK^41^. These kinases phosphorylate SREBFs, enhancing their transcriptional activity^40^. Thus, the inhibition of MAPKs through DUPS1 decreases the transcriptional activity of SREBFs and therefore lipogenesis. *FGFR1* codifies for the fibroblast growth factor receptor 1 and binds to circulating fibroblast growth factor 21 (FGF21)^42^, which is a potent regulator of metabolism^43^,^44^. FGFR21 acts as an inhibitor of the SREBP signaling pathway^45^, and its expression is modified under different dietary conditions^46^,^47^,^48^. However, it has been reported that the mammary gland is not one of the target tissues of FGF21^49^. Moreover, to induce FGF21 signaling, FGFR1 must be associated with the co-receptor proteins Klotho or β-Klotho^50^,^51^, codified by *KL* and *KLB*. In our study, *KL* was not differentially expressed, and *KLB* exhibited null expression levels. *ATF3* encodes the activating transcription factor 3, a transcriptional repressor. This gene is upregulated under extracellular and intracellular stress conditions^52^,^53^. Moreover, *ATF3* is a target gene in the regulation of inflammation, immunity and lipid metabolism^54^,^55^,^56^, which are biological processes influenced by n-3 PUFA^37^,^57^,^58^. Finally, within the upregulated genes of the FO-MFD condition, we identified other transcription factors, such as Kruppel-like factors 6 and 11 (*KLF6* and *KLF11*). Contrary to our expectations, *KLF6* seems to promote PPARα^59^, which would enhance SREBP transcriptional activity^60^. However, consistent with our results, *KLF11* was upregulated in a study on the transcriptome adaptation of the bovine mammary gland to diets rich in unsaturated FAs^30^.

## Conclusions

This study presents for the first time in the scientific literature a comprehensive profile of the transcriptomic changes occurring in the mammary gland of lactating sheep suffering from FO-induced MFD. Our results indicate that the reduction in milk fat is associated with the downregulation of key genes involved in the lipogenesis. Moreover, the study suggests that some pathways leading to lipid metabolism downregulation could be related to the upregulation of genes involved in signal transduction and codifying for transcription factors. Overall, these results represent a key application of RNA-Seq technology in ruminant nutrigenomics and provide a great advance in the understanding of functional genomic mechanisms associated with marine lipid-induced MFD in dairy sheep. Our results include a wide list of genes that have not been traditionally analyzed in candidate gene studies in ruminants but may be key regulators in the MFD process and very useful for further research in this field.

## Methods

### Animals, diets and experimental design

Details of experimental procedures are described in Toral *et al*. (2016)^29^. Briefly, eight lactating Assaf ewes at mid lactation (62 ± 2.5 days in milk at the beginning of the assay; parity = 2 ± 0.3) were used. The ewes were individually housed and divided in 2 groups (n = 4) that were assigned to one of 2 dietary treatments: no lipid supplementation (Control) or supplementation with 24 g of fish oil (Afampes 121 DHA; Afamsa, Mos, Spain)/kg of diet dry matter (FO). The basal diet consisted of a total mixed ration based on alfalfa hay (40%) and concentrates (60%). The oil was weekly mixed with the ration, which included molasses to reduce selection of components. All ewes were fed the control diet for a 21-d adaptation period and then both experimental treatments for 40 more days. Samplings for this study were performed at the end of the experiment, when ewes on the FO treatment exhibited a stable decrease in milk fat concentration (monitored daily by infrared spectrophotometric analysis of raw milk samples; ISO 9622:1999). Diets were offered ad libitum, and refusals were removed and weighed each morning. Animals were milked twice daily in a single side milking parlor (DeLaval, Madrid, Spain).

As detailed in the companion study^29^, feed intake was similar in both groups (on average, 2.72 kg/d), but ewes fed FO exhibited a MFD characterized by a significantly reduced milk fat concentration (in %, 5.87 vs. 4.19, SED = 0.262). On the other hand, milk production was increased in FO-MFD compared with control (1.92 vs 1.47 kg/d respectively, SED = 0.124). Milk FA composition was also different depending on the diet, and details about FA profiles in control and FO-MFD sheep are reported in Toral *et al*.^29^.

### Collection of milk samples and RNA extraction

Milk samples for RNA extraction were collected on days 38 and 39 on treatments. As previous explained by Suárez-Vega *et al*.^61^, to maximize the concentration of mammary epithelial cells within MSCs, the collection was performed approx. 60–70 min after milking and 10 minutes after the injection of oxytocin (5 IU/animal; Facilpart, Laboratorios SYVA, León, Spain). Udders were cleaned with water and soap and then disinfected with povidone iodine. Nipples were also washed with RNAseZap (Ambion, Austin, TX). Individual samples were obtained by hand-milking each half of the mammary gland into an RNAse-free 50-mL tube (2 samples/ewe), which was covered with a sterile gauze to filter the milk. All protocols involving animals were approved by the Animal Welfare Committee of the Instituto de Ganadería de Montaña, the Spanish National Research Council (CSIC) and the Junta de Castilla y León (Spain), following proceedings described in Spanish and EU legislations (R.D. 53/2013, and Council Directive 2010/63/EU). All animals used in this study were handled in strict accordance with good clinical practices, and all efforts were made to minimize suffering.

Samples were held in ice and transferred immediately to the laboratory. Total RNA from MSCs was extracted from 50 mL of fresh milk^20^,^61^. Briefly, MSCs were pelleted by centrifugation at 650 × *g* for 10 min at 4 °C in the presence of a final concentration of 0.5 mM of EDTA. The cell pellet was washed with 10 mL of PBS (pH 7.2 and 0.5 mM of EDTA) followed by an additional centrifugation at 650 × *g* for 10 min at 4 °C. Washing and centrifugation procedures were repeated twice using 2 and 1.5 mL of the same PBS solution. Then, total RNA was extracted and purified from the milk cell pellet with 500 μL of TRIzol (Invitrogen, Carlsbad, CA).

### RNA sequencing

The integrity of the RNA was assessed by the Agilent 2100 Bioanalyzer device (Agilent Technologies, Santa Clara, CA, USA). For each animal, samples with the greatest RNA integrity value (RIN) were used in subsequent analyses. The mean RIN of the selected samples was 8.0 ± 0.13 (range 6.8–8.4). Stranded paired-end libraries with fragments of 300 bp were prepared using the TruSeq Stranded Total RNA Library Prep Kit (Illumina, San Diego, CA, USA). The fragments were sequenced on an Illumina Hi-Seq 2000 sequencer (Fasteris SA, Plan-les-Ouates, Switzerland) according to the manufacturer’s instructions at CNAG (Centro Nacional de Análisis Genómico, Barcelona, Spain), generating stranded paired-end reads of 75 bp. Eight samples from four control and four FO-MFD ewes were sequenced to a minimum depth of 30 million paired-end reads. One of the control samples, which did not reach the minimum depth fixed, was re-sequenced.

### Quality control, mapping and quantification

The quality of the raw sequencing data was assessed using FastQC^62^. STAR aligner v2.4.0^63^ was used to map the reads against the ovine genome assembly v.3.1. (Oar_v3.1; ftp://ftp.ensembl.org/pub/release-83/fasta/ovis_aries/).

For the quantification of gene expression, the annotation of the Oar_v3.1 (ftp://ftp.ensembl.org/pub/release-83/gtf/ovis_aries/) sheep assembly was used as reference. The HTSeq-count v0.6.1^64^ was used to count the number of reads mapped to each gene. Cuffnorm and Cuffquant from Cufflinks package^65^ were used to obtain normalized FPKM reads.

### Power calculations

The web-based software Scotty (http://scotty.genetics.utah.edu/) was used to estimate the power of detection of DE genes of the RNA-Seq experiment. For these calculations, we uploaded the quantification file obtained with HTSeq-count v0.6.1^64^ and fixed the following parameters: a cost per replicate of 200 US Dollars (USD), a cost per million reads aligned to genes of 100 USD, an alignment rate of 85%, a maximum of 10 replicates per condition, a read depth between 10 and 40 million, a maximum cost of the experiment of 100,000 USD, a 50% of differential expressed genes detected with a fold change of 2 and a *p-value* of 0.05 and a minimum of 50% of genes with at least 50% of maximum power.

### Differential expression analysis

Differential gene expression analysis between the milk transcriptomes from FO-MFD and control groups was performed using the DESeq2 (v1.12.4)^66^ in the R statistical software program (v3.3.1). First, we used DESeq2 to collapse the technical replicates generated for one of the control samples. Then, the program normalized gene count data correcting for library size and RNA composition bias and estimated the dispersion across biological replicates. Finally, pairwise comparison of expression was made between the FO-MFD and the control group for every gene based on a negative binomial model. Fold changes and their associated p-values were obtained. After this step, DESeq2 performs independent filtering, which removes genes that have low counts with the aim of improving the detection power by making the multiple testing adjustment of the p-values less severe. P-values from the subset of genes that pass independent filtering step are adjusted by Benjamini-Hochberg’s approach to correct for multiple testing. Genes with a *p*_*adj*_ < 0.05 were assigned as differentially expressed. Moreover, DEseq2 was used to perform a principal component analysis (PCA) to cluster samples based on gene expression data.

### Gene functional classification: GO term enrichment analysis

The web-based Gene Set Analysis Toolkit (WebGestalt)^17^ was used to perform a Gene-Ontology (GO) enrichment and WikiPathways analysis. Enriched terms were considered statistically significant when *p*_*adj*_ was <0.05, and a minimum of five genes were grouped for each significant term. GO terms were categorized in three major functional groups: biological process, molecular function and cellular component.

### Quantitative Real-time PCR (qRT-PCR)

To validate the results from the differential expression analysis performed with DESeq2, the mRNA abundances of a subset of 12 genes (4 upregulated, 4 downregulated and 4 non-differentially expressed) were analyzed by qRT-PCR as described in the companion study^29^ using the primer sequences shown in Supplementary File S7.

## Additional Information

**How to cite this article:** Suárez-Vega, A. *et al*. Elucidating fish oil-induced milk fat depression in dairy sheep: Milk somatic cell transcriptome analysis. *Sci. Rep.* **7**, 45905; doi: 10.1038/srep45905 (2017).

**Publisher's note:** Springer Nature remains neutral with regard to jurisdictional claims in published maps and institutional affiliations.

## Supplementary Material

Supplementary Files
