# Supplementary material for: Elucidating fish oil-induced milk fat depression in dairy sheep: Milk somatic cell transcriptome analysis
Source: Sci Rep. 2017 Apr 5;7:45905. doi: 10.1038/srep45905 (PMC5381099; doi:10.1038/srep45905)
Supplement: Supplementary Files [file srep45905-s1.pdf]

Elucidating fish oil-induced milk fat depression in dairy sheep: Milk somatic cell transcriptome analysis.

Aroa Suárez-Vega[1], Pablo G. Toral[2], Beatriz Gutiérrez-Gil[1], Gonzalo Hervás[2], Juan José Arranz[1]* & Pilar Frutos[2]

[1] Departamento de Producción Animal, Facultad de Veterinaria, Universidad de León, Campus de Vegazana s/n, León 24071, Spain

[2] Instituto de Ganadería de Montaña (CSIC-ULE), Finca Marzanas s/n, Grulleros 24346, León, Spain

*Corresponding author: Juan Jose Arranz (email address: jjarrs@unileon.es)

Supplementary Files

Index

Supplementary File S1 3

Supplementary File S2 7

Supplementary File S3 12

Supplementary File S4 18

Supplementary File S5 31

Supplementary File S6..72

Supplementary File S7 74

Supplementary File S1

Title of data: Power calculations results

Description of the data: Results from the power calculation analysis performed with Scotty (http://scotty.genetics.utah.edu/)

Upload: sheep_milk_powercalculator_FO.txt
File upload succeeded.

User Inputs Used in the Analysis

Control columns in pilot data: 5
Test columns in pilot data: 4
Cost per replicate, control: $200
Cost per replicate, test: $200
Cost per million reads: $100
Alignment Rate: 85%
Maximum cost of experiment: $100000
Percentage of genes detected: 50
At p value cutoff: 0.05
For the following true fold change: 2
Maximum percentage of genes with low-powered (biased) measurements: 50

Export To PDF

Summary of Findings

Scotty has tested 90 possible experimental designs.

The following experiments meet your criteria:

> Least expensive: 5 replicates sequenced to a depth of 10 million reads aligned to genes per replicate.
> Most powerful: 10 replicates sequenced to a depth of 40 million reads aligned to genes per replicate.

The number of samples that is required is in part determined by how dispersed your biological replicates are. We measured the dispersion of your replicates:

> Control samples replicate dispersion: 0.20439
> Test samples replicate dispersion: 0.19924

The dispersion metric that Scotty uses is the mean overdispersion from Poisson. Many factor can affect how dispersed replicates are. For a general reference, most of the biological replicate pairs we examined had an overdipsersion between 0.2 and 0.4.

We measured the number of unique genes observed in you data (detected by at least one read in one or the samples) and estimated the number of genes that are expressed:

> Genes observed (Control): 15993
> Genes observed (Test): 16112

Power calculations (the % detected) are based on the number of observed genes.

Excluded Experimental Configurations

This shows the power that will be achieved in each experimental configuration.
Filled in boxes are not allowed for the reasons stated in the key.

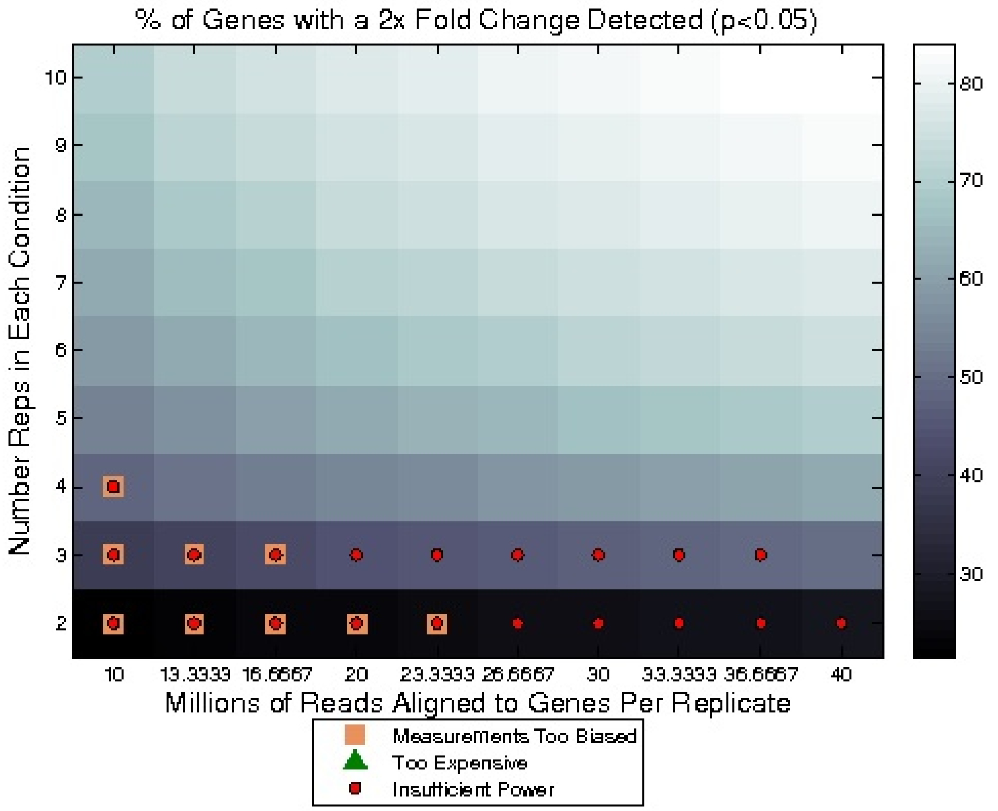

Allowed Experimental Configurations

This shows experimental configurations which are and are not allow under the user defined optimization parameters. White boxes are allowed. Red boxes are not allowed .

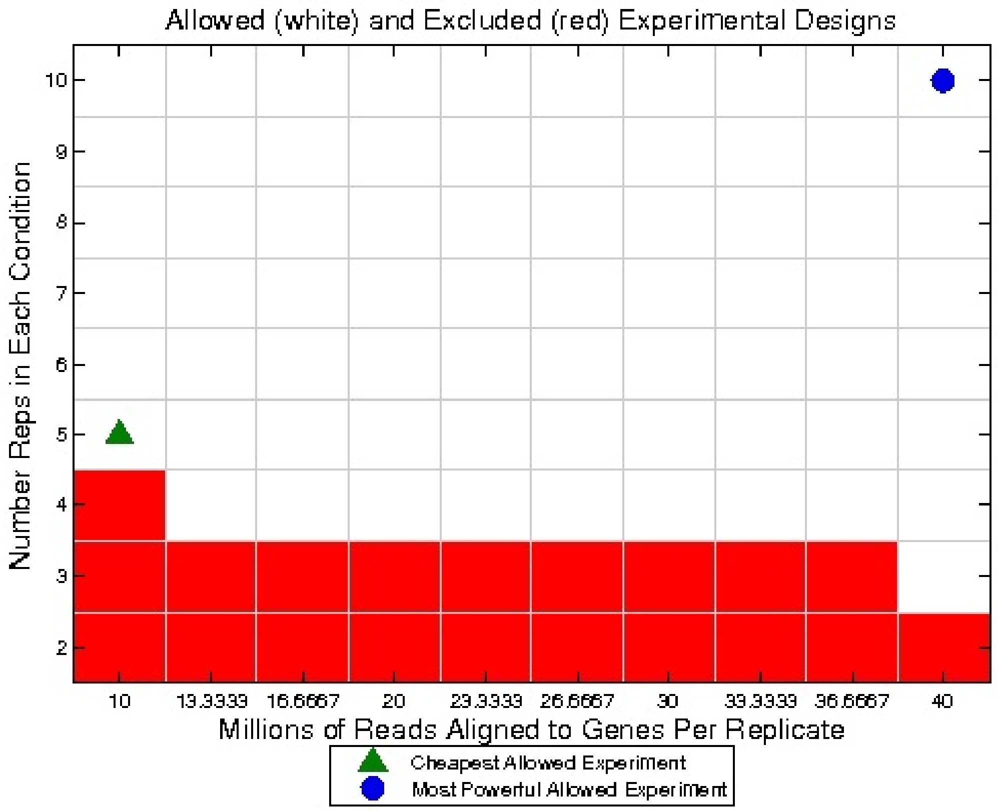

Predicted Statistical Power

The least expensive experiment that meets your criteria will yield the following power:

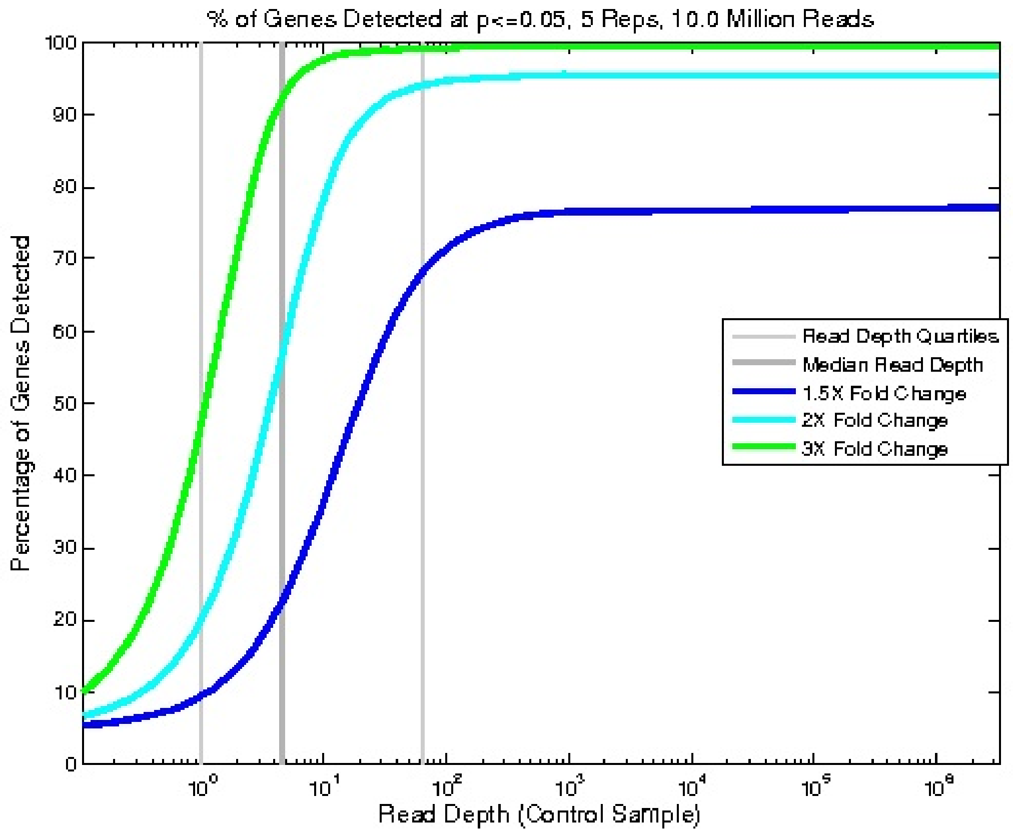

The most powerful experiment that meets your criteria will yield the following power:

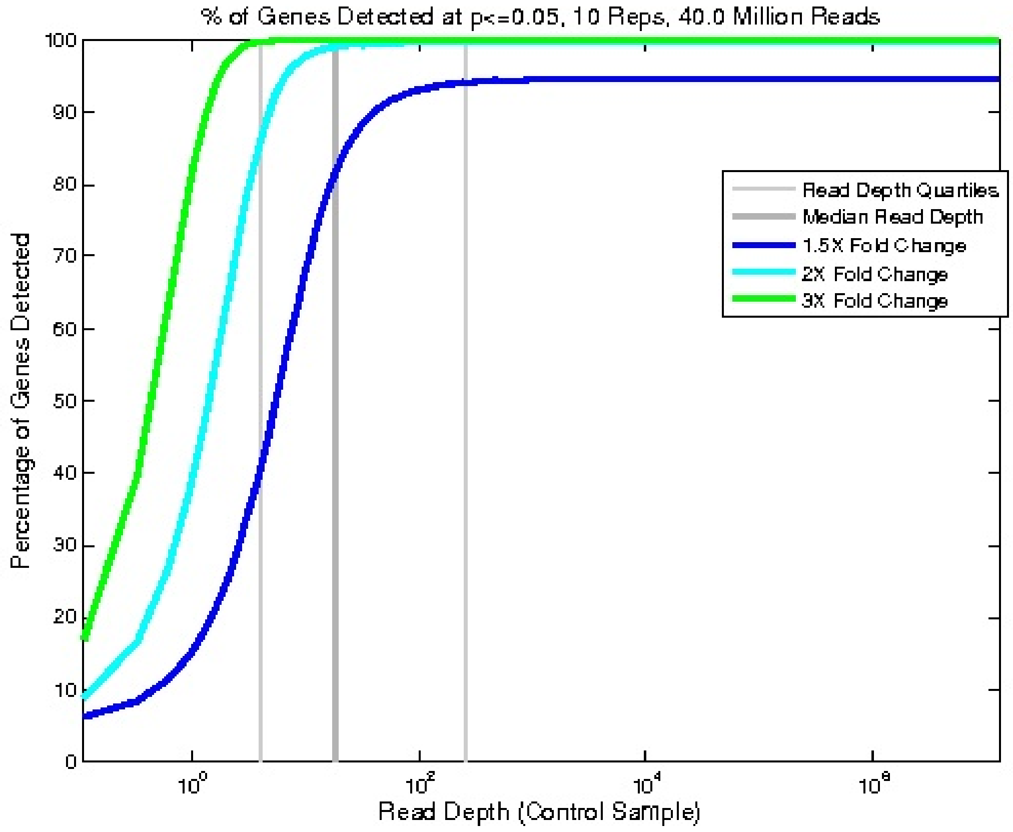

Supplementary File S2

Title of data: Results from the Wikipathway analysis of core genes identified in the transcriptome of the control and the fish oil milk fat depression (FO-MFD) ewes analyzed in this study.

Description of data: This file provides the results from the Wikipathway analysis performed with WebGestalt for the core genes (genes with > 180 FPKM).

Wikipathway enrichment analysis: Core genes (>180 FPKM) identified in both conditions

Database:Wikipathways pathway Name:Cytoplasmic Ribosomal Proteins ID:WP477
C=88; O=31; E=0.24; R=128.75; rawP=2.35e-58; adjP=7.75e-57

Index	UserID	Gene Name
1	RPS8	ribosomal protein S8
2	RPL29	ribosomal protein L29
3	RPS25	ribosomal protein S25
4	RPL8	ribosomal protein L8
5	RPL38	ribosomal protein L38
6	RPS24	ribosomal protein S24
7	RPS5	ribosomal protein S5
8	RPL18A	ribosomal protein L18a
9	RPL27A	ribosomal protein L27a
10	RPS3A	ribosomal protein S3A
11	RPS17	ribosomal protein S17
12	RPL11	ribosomal protein L11
13	RPL7A	ribosomal protein L7a
14	RPL12	ribosomal protein L12
15	RPS28	ribosomal protein S28
16	RPL18	ribosomal protein L18
17	RPL32	ribosomal protein L32
18	RPL28	ribosomal protein L28
19	RPS27A	ribosomal protein S27a
20	RPS11	ribosomal protein S11
21	RPS14	ribosomal protein S14
22	RPS19	ribosomal protein S19
23	RPS6	ribosomal protein S6
24	RPL19	ribosomal protein L19
25	RPL37A	ribosomal protein L37a
26	RPS21	ribosomal protein S21
27	RPS12	ribosomal protein S12
28	RPS3	ribosomal protein S3
29	RPL22	ribosomal protein L22
30	FAU	Finkel-Biskis-Reilly murine sarcoma virus (FBR-MuSV) ubiquitously expressed
31	RPS26	ribosomal protein S26

Database:Wikipathways pathway Name:Electron Transport Chain ID:WP111
C=103; O=9; E=0.28; R=31.94; rawP=1.27e-11; adjP=4.19e-10

Index	UserID	Gene Name
1	COX5B	cytochrome c oxidase subunit Vb
2	SLC25A4	solute carrier family 25 (mitochondrial carrier; adenine nucleotide translocator), member 4

		ATP synthase, H+ transporting, mitochondrial Fo complex, subunit C2
3	ATP5G2	(subunit 9)
4	UQCRB	ubiquinol-cytochrome c reductase binding protein
5	COX6A1	cytochrome c oxidase subunit VIa polypeptide 1
6	UQCR10	ubiquinol-cytochrome c reductase, complex III subunit X
		ATP synthase, H+ transporting, mitochondrial Fo complex, subunit F6
7	ATP5J	
		solute carrier family 25 (mitochondrial carrier; adenine nucleotide translocator), member 6
8	SLC25A6	
9	COX6B1	cytochrome c oxidase subunit VIb polypeptide 1 (ubiquitous)

Database:Wikipathways pathway Name:Oxidative Stress ID:WP408
C=30; O=3; E=0.08; R=36.55; rawP=7.68e-05; adjP=0.0025

Index	UserID	Gene Name
1	GPX3	glutathione peroxidase 3 (plasma)
2	MGST1	microsomal glutathione S-transferase 1
3	XDH	xanthine dehydrogenase

Database:Wikipathways pathway Name:Pentose Phosphate Pathway ID:WP134
C=8; O=2; E=0.02; R=91.37; rawP=0.0002; adjP=0.0066

Index	UserID	Gene Name
1	PGD	phosphogluconate dehydrogenase
2	TKT	transketolase

Database:Wikipathways pathway Name:Diurnally regulated genes with circadian orthologs
C=48; O=3; E=0.13; R=22.84; rawP=0.0003; adjP=0.0099 ID:WP410

Index	UserID	Gene Name
1	UGP2	UDP-glucose pyrophosphorylase 2
2	EIF4G2	eukaryotic translation initiation factor 4 gamma, 2
3	HSPA8	heat shock 70kDa protein 8

Wikipathway enrichment analysis: Core genes (>180 FPKM) identified in control samples

Database:Wikipathways pathway Name:SREBP signalling ID:WP1982	
C=83; O=2; E=0.03; R=61.13; rawP=0.0005; adjP=0.0005	
Gene symbol	Gene Name
INSIG1	insulin induced gene 1
DBI	diazepam binding inhibitor (GABA receptor modulator, acyl-CoA binding protein)

Wikipathway enrichment analysis: Core genes (>180 FPKM) identified in fish oil induced milk fat depression samples

Database:Wikipathways pathway Name:Selenium Metabolism and Selenoproteins
C=49; O=2; E=0.01; R=220.04; rawP=3.53e-05; adjP=3.53e-05 ID:WP28

Index	UserID	Gene Name
1	SELENBP1	selenium binding protein 1
2	FOS	FBJ murine osteosarcoma viral oncogene homolog

Supplementary File S3

Title of data: Differentially expressed genes

Description of data: Results from the differential expression analysis performed with DESeq2.

GENE	baseMean	log2FoldChange	lfcSE	stat	pvalue	padj
PON3	185.2096	1.7536	0.2602	6.7405	1.58E-11	1.8851E-07
LPIN1	1093.2689	-1.7759	0.2836	-6.2626	3.79E-10	2.2602E-06
ANGPTL4	708.1334	1.4698	0.2469	5.9529	2.63E-09	1.0487E-05
ENSOARG00000009821	17.8750	1.8552	0.3252	5.7047	1.17E-08	3.4789E-05
CYR61	1083.3347	1.6414	0.2951	5.5625	2.66E-08	6.3521E-05
SERTM1	32.7592	-1.7838	0.3240	-5.5051	3.69E-08	7.3419E-05
POSTN	134.0736	1.6803	0.3089	5.4397	5.34E-08	9.1011E-05
FGFR1	150.9002	1.6334	0.3017	5.4150	6.13E-08	9.1498E-05
RETSAT	2836.9367	1.5383	0.2886	5.3295	9.85E-08	1.3062E-04
FAM189A2	114.1224	1.2870	0.2460	5.2310	1.69E-07	1.9837E-04
RPA1	665.6275	-0.7172	0.1375	-5.2161	1.83E-07	1.9837E-04
GNA14	14.0298	1.6791	0.3265	5.1426	2.71E-07	2.6955E-04
FOSB	884.1845	1.3401	0.2616	5.1223	3.02E-07	2.7727E-04
ENSOARG00000000673	25.3490	1.6442	0.3242	5.0722	3.93E-07	3.3537E-04
ACSS2	18052.6036	-1.0196	0.2052	-4.9683	6.75E-07	4.6036E-04
BPIFB1	876.0911	1.4373	0.2914	4.9330	8.10E-07	4.6036E-04
CLDN8	191.8132	1.4204	0.2865	4.9584	7.11E-07	4.6036E-04
FADS2	686.0614	-1.4887	0.3015	-4.9368	7.94E-07	4.6036E-04
HSPB8	72.9944	-1.2301	0.2477	-4.9660	6.84E-07	4.6036E-04
PNPLA3	681.0781	-1.2048	0.2412	-4.9954	5.87E-07	4.6036E-04
PTPRU	16.3313	1.5787	0.3188	4.9515	7.36E-07	4.6036E-04
TYR	71.5188	-1.3348	0.2729	-4.8914	1.00E-06	5.4344E-04
ARL6IP5	132.1098	0.8772	0.1801	4.8700	1.12E-06	5.7939E-04
ACACA	6134.4792	-0.8756	0.1823	-4.8039	1.56E-06	7.1460E-04
F5	189.0251	1.2008	0.2493	4.8169	1.46E-06	7.1460E-04
RAD21	1034.8278	-0.6895	0.1433	-4.8109	1.50E-06	7.1460E-04
LRIG3	56.0817	1.3479	0.2820	4.7801	1.75E-06	7.7482E-04
PDE8A	112.7326	-0.8650	0.1839	-4.7024	2.57E-06	1.0585E-03
ENSOARG00000014201	2011.1837	-0.8225	0.1748	-4.7052	2.54E-06	1.0585E-03
C9orf152	202.8163	-1.0690	0.2301	-4.6458	3.39E-06	1.3482E-03
ENSOARG00000013560	88.7621	1.3478	0.2906	4.6378	3.52E-06	1.3560E-03
ACSL1	6122.7314	-0.8726	0.1892	-4.6115	4.00E-06	1.3934E-03
BMP1	288.5709	0.9080	0.1973	4.6014	4.20E-06	1.3934E-03
FCGRT	109.6419	1.1539	0.2508	4.6012	4.20E-06	1.3934E-03
SEPT10	206.7076	-0.7714	0.1675	-4.6061	4.10E-06	1.3934E-03
ENSOARG00000008223	153.3780	-0.8070	0.1752	-4.6048	4.13E-06	1.3934E-03
PCYT2	777.5871	-0.9428	0.2072	-4.5506	5.35E-06	1.7261E-03
TCTN3	34.5642	1.3986	0.3083	4.5362	5.73E-06	1.7998E-03
ASS1	470.5632	-1.1488	0.2536	-4.5306	5.88E-06	1.8010E-03
CAMK4	39.6789	-1.3816	0.3071	-4.4980	6.86E-06	2.0473E-03
ARHGAP18	385.8750	-0.6982	0.1559	-4.4774	7.55E-06	2.1999E-03
ENSOARG00000020086	9.9299	1.4472	0.3237	4.4706	7.80E-06	2.2179E-03
CTSF	190.5410	0.8272	0.1854	4.4620	8.12E-06	2.2544E-03
FGD2	93.7153	-1.2102	0.2721	-4.4477	8.68E-06	2.3555E-03
PRUNE2	83.2192	-1.3994	0.3162	-4.4256	9.62E-06	2.4597E-03
RASEF	965.6428	-0.8470	0.1917	-4.4196	9.89E-06	2.4597E-03
STAR	72.4680	1.1491	0.2600	4.4205	9.85E-06	2.4597E-03
TMEM168	132.8630	1.0799	0.2438	4.4294	9.45E-06	2.4597E-03
FST	62.1479	-1.4137	0.3214	-4.3986	1.09E-05	2.6014E-03

GCA	47.4011	1.4327	0.3257	4.3990	1.09E-05	2.6014E-03
ENSOARG00000002759	979.8547	-0.8169	0.1860	-4.3914	1.13E-05	2.6363E-03
CCDC129	150.0980	1.0989	0.2519	4.3630	1.28E-05	2.8570E-03
MEGF10	100.8496	1.3805	0.3165	4.3614	1.29E-05	2.8570E-03
RUSC1	518.5335	-1.0264	0.2350	-4.3679	1.25E-05	2.8570E-03
CYP7B1	26.7945	1.3841	0.3197	4.3291	1.50E-05	3.2496E-03
GPNMB	132.2383	1.3142	0.3043	4.3183	1.57E-05	3.2830E-03
SLC9A4	8.0033	-1.3943	0.3231	-4.3152	1.59E-05	3.2830E-03
WFDC2	162.6382	-0.9613	0.2225	-4.3196	1.56E-05	3.2830E-03
LSS	378.3107	-0.9733	0.2263	-4.3018	1.69E-05	3.4285E-03
ENSOARG00000006401	68.2395	-1.3399	0.3121	-4.2929	1.76E-05	3.5089E-03
RASGEF1A	62.6137	1.1717	0.2750	4.2607	2.04E-05	3.8624E-03
SLC22A16	2072.1138	0.6366	0.1493	4.2637	2.01E-05	3.8624E-03
TNFRSF21	3906.9108	0.8824	0.2068	4.2674	1.98E-05	3.8624E-03
SLC6A20	50.5732	-1.3608	0.3205	-4.2460	2.18E-05	4.0604E-03
ALDOA	3379.4164	-0.5389	0.1275	-4.2280	2.36E-05	4.2231E-03
CYP39A1	36.3200	1.1652	0.2752	4.2332	2.30E-05	4.2231E-03
ENSOARG00000014790	12662.0357	0.8776	0.2076	4.2269	2.37E-05	4.2231E-03
DUSP1	862.6284	1.2014	0.2849	4.2166	2.48E-05	4.3539E-03
ACSS3	1547.0526	-0.7079	0.1683	-4.2073	2.58E-05	4.4713E-03
APP	1426.4592	0.9769	0.2326	4.1992	2.68E-05	4.5681E-03
DAB1	53.5785	-1.1307	0.2695	-4.1957	2.72E-05	4.5750E-03
CYBA	278.3109	0.7220	0.1728	4.1774	2.95E-05	4.8231E-03
FAM107A	183.7396	-0.8969	0.2147	-4.1782	2.94E-05	4.8231E-03
NOTCH3	28.6374	1.3338	0.3209	4.1564	3.23E-05	5.1459E-03
ENSOARG00000012720	560.1028	-0.5636	0.1356	-4.1572	3.22E-05	5.1459E-03
SPRY1	65.4834	1.0449	0.2524	4.1391	3.49E-05	5.4789E-03
LGR4	567.8745	1.0854	0.2632	4.1233	3.74E-05	5.7924E-03
EGR2	229.0143	1.0118	0.2464	4.1062	4.02E-05	6.1578E-03
ATF4	12381.6462	-0.6080	0.1485	-4.0943	4.23E-05	6.1654E-03
C8orf4	190.7617	1.2292	0.3002	4.0953	4.22E-05	6.1654E-03
SEMA4F	12.4524	1.3416	0.3271	4.1013	4.11E-05	6.1654E-03
ENSOARG00000019966	275.0441	-0.7615	0.1858	-4.0991	4.15E-05	6.1654E-03
JUN	1942.3351	1.0576	0.2591	4.0812	4.48E-05	6.4458E-03
GPX8	114.6021	0.9943	0.2441	4.0742	4.62E-05	6.5630E-03
CDC42EP4	132.9175	-0.6981	0.1733	-4.0278	5.63E-05	7.9071E-03
MPZL1	228.2428	0.7006	0.1748	4.0073	6.14E-05	8.4284E-03
SLC7A6	130.7969	0.6917	0.1726	4.0085	6.11E-05	8.4284E-03
MVD	590.6917	-1.1079	0.2773	-3.9946	6.48E-05	8.6924E-03
PTGFRN	25.5813	1.2182	0.3049	3.9951	6.47E-05	8.6924E-03
HMGCS1	909.8700	-1.0589	0.2657	-3.9855	6.73E-05	8.9333E-03
ENSOARG00000001694	356.5119	-0.8385	0.2110	-3.9732	7.09E-05	9.3043E-03
KCTD14	271.1270	-0.7828	0.1972	-3.9692	7.21E-05	9.3465E-03
SLC22A5	267.9179	0.8222	0.2073	3.9669	7.28E-05	9.3465E-03
SLC9A3R1	625.2940	-0.4904	0.1241	-3.9536	7.70E-05	9.7788E-03
ENSOARG00000004438	1831.9871	-0.6733	0.1712	-3.9321	8.42E-05	1.0583E-02
CCNL1	348.8069	0.9409	0.2404	3.9133	9.10E-05	1.0980E-02
EFHD1	1954.1184	-0.7410	0.1893	-3.9140	9.08E-05	1.0980E-02
PDE4DIP	278.3239	-0.8048	0.2055	-3.9153	9.03E-05	1.0980E-02
STARD4	237.2062	-0.9520	0.2431	-3.9162	8.99E-05	1.0980E-02

AACS	1792.7716	-0.7483	0.1915	-3.9086	9.28E-05	1.1085E-02
G6PD	1521.8771	-0.7367	0.1888	-3.9031	9.50E-05	1.1228E-02
PCK2	658.2056	-0.7252	0.1861	-3.8968	9.75E-05	1.1410E-02
CD320	229.0468	0.9777	0.2518	3.8827	0.000103	1.1939E-02
NFE2L3	74.2900	0.9849	0.2538	3.8811	0.000104	1.1939E-02
ENSOARG00000005716	8.6673	-1.2585	0.3273	-3.8451	0.000121	1.3704E-02
NFKBIZ	500.9851	1.1750	0.3060	3.8402	0.000123	1.3717E-02
ENSOARG00000003744	38.2008	1.1927	0.3106	3.8402	0.000123	1.3717E-02
ANKRD33B	14.6254	1.2306	0.3218	3.8235	0.000132	1.4231E-02
AZGP1	182.2682	-1.1050	0.2888	-3.8262	0.00013	1.4231E-02
RASAL2	291.9732	0.7824	0.2047	3.8221	0.000132	1.4231E-02
ENSOARG00000006465	530.8772	-0.7480	0.1954	-3.8282	0.000129	1.4231E-02
APBA1	83.6541	0.9622	0.2528	3.8067	0.000141	1.5013E-02
FDPS	84.7551	-0.9861	0.2597	-3.7975	0.000146	1.5443E-02
ENSOARG00000002516	59.8393	1.0930	0.2880	3.7951	0.000148	1.5458E-02
ATF3	2118.1668	0.8493	0.2239	3.7929	0.000149	1.5460E-02
AMOTL2	204.8165	1.1285	0.2982	3.7843	0.000154	1.5864E-02
ZSWIM4	150.7157	1.0165	0.2698	3.7681	0.000164	1.6786E-02
FCGR2B	413.4834	0.8738	0.2321	3.7652	0.000166	1.6842E-02
DSG3	14.0730	1.2239	0.3255	3.7598	0.00017	1.7065E-02
ATP6V0D1	761.1362	-0.5936	0.1580	-3.7572	0.000172	1.7095E-02
LIFR	925.1202	1.0347	0.2763	3.7450	0.00018	1.7798E-02
EPB41L4B	356.9668	-0.6482	0.1734	-3.7373	0.000186	1.8016E-02
POC5	65.1690	0.8147	0.2181	3.7358	0.000187	1.8016E-02
PPM1K	292.5310	-0.6988	0.1869	-3.7394	0.000184	1.8016E-02
C5orf42	18.5950	1.2019	0.3228	3.7234	0.000197	1.8353E-02
CRELD1	144.7966	0.8085	0.2171	3.7248	0.000195	1.8353E-02
NXPE3	36.7188	-1.0995	0.2951	-3.7255	0.000195	1.8353E-02
SLC31A2	175.1625	0.7976	0.2142	3.7232	0.000197	1.8353E-02
DDX1	2394.4510	-0.5682	0.1528	-3.7188	0.0002	1.8527E-02
MAML2	22.7117	1.1089	0.2985	3.7148	0.000203	1.8610E-02
SLC5A9	395.0999	0.9566	0.2576	3.7138	0.000204	1.8610E-02
ABCC10	230.4426	0.9309	0.2515	3.7010	0.000215	1.9276E-02
KCNJ2	43.3502	1.1531	0.3115	3.7020	0.000214	1.9276E-02
PAFAH1B3	172.8721	-0.6362	0.1736	-3.6659	0.000246	2.1809E-02
ENSOARG00000016817	1644.3051	-0.3793	0.1035	-3.6658	0.000247	2.1809E-02
ENSOARG00000008097	22.6634	1.1660	0.3190	3.6554	0.000257	2.2546E-02
ERRFI1	397.4800	1.0430	0.2857	3.6504	0.000262	2.2670E-02
HAUS5	96.2272	0.8247	0.2259	3.6502	0.000262	2.2670E-02
RNF41	362.4668	-0.5394	0.1479	-3.6472	0.000265	2.2771E-02
ASAP2	187.0518	-0.5631	0.1548	-3.6377	0.000275	2.3463E-02
ECHDC3	290.9920	-0.7616	0.2097	-3.6327	0.00028	2.3750E-02
ABCA3	190.7273	0.7002	0.1939	3.6107	0.000305	2.5676E-02
SLC40A1	52.1972	1.1213	0.3110	3.6058	0.000311	2.5984E-02
ENSOARG00000017439	242.3915	0.6898	0.1921	3.5910	0.000329	2.7313E-02
CHMP4C	35.6019	-1.0232	0.2856	-3.5827	0.00034	2.7876E-02
YWHAH	307.7494	-0.5623	0.1570	-3.5821	0.000341	2.7876E-02
EXOG	39.6638	0.9744	0.2722	3.5793	0.000344	2.7979E-02
PPP1R10	520.3119	0.7761	0.2170	3.5771	0.000347	2.8031E-02
PM20D1	89.3934	1.0704	0.3001	3.5671	0.000361	2.8727E-02

ENSOARG00000011060	197.1126	-0.6730	0.1886	-3.5686	0.000359	2.8727E-02
CSF2RB	21339.2576	0.9818	0.2760	3.5573	0.000375	2.9437E-02
SCARA3	299.6070	0.6500	0.1827	3.5581	0.000374	2.9437E-02
KLF6	724.6061	0.9609	0.2712	3.5435	0.000395	3.0815E-02
KRT24	20.9149	1.1566	0.3271	3.5361	0.000406	3.1161E-02
MVP	1117.9218	-0.5085	0.1437	-3.5378	0.000403	3.1161E-02
SMAP2	333.8486	-0.6033	0.1706	-3.5354	0.000407	3.1161E-02
BAG1	3025.5713	-0.4864	0.1379	-3.5269	0.000421	3.1797E-02
ENSOARG00000020248	245.3497	-0.6418	0.1820	-3.5267	0.000421	3.1797E-02
FHOD3	153.8925	0.8828	0.2507	3.5208	0.00043	3.2312E-02
RND1	1746.8302	1.0465	0.2978	3.5146	0.00044	3.2863E-02
CIT	455.5092	-0.9560	0.2725	-3.5084	0.000451	3.3226E-02
ENSOARG00000004224	101.3710	1.0458	0.2981	3.5084	0.000451	3.3226E-02
HS3ST1	15.7542	1.1275	0.3215	3.5064	0.000454	3.3272E-02
CCNT2	263.8788	0.6893	0.1967	3.5045	0.000458	3.3312E-02
ENSOARG00000016449	752.1664	-0.4593	0.1312	-3.5015	0.000463	3.3473E-02
KLF11	27.5965	1.0940	0.3142	3.4817	0.000498	3.5841E-02
AGPAT2	47.1691	-0.9965	0.2872	-3.4701	0.00052	3.5927E-02
CDIP1	8.7285	-1.0823	0.3118	-3.4708	0.000519	3.5927E-02
CLIC2	57.6686	-0.9063	0.2607	-3.4761	0.000509	3.5927E-02
FCGBP	1500.5892	1.1321	0.3259	3.4735	0.000514	3.5927E-02
LSM12	365.9629	-0.6460	0.1859	-3.4748	0.000511	3.5927E-02
MBNL3	115.0017	-0.8876	0.2558	-3.4700	0.000521	3.5927E-02
SDSL	658.0451	-0.8249	0.2372	-3.4780	0.000505	3.5927E-02
PPT1	1116.8058	0.7641	0.2204	3.4677	0.000525	3.6026E-02
MCL1	2830.9557	0.6436	0.1858	3.4648	0.000531	3.6095E-02
NRSN2	10.4407	-1.1337	0.3273	-3.4641	0.000532	3.6095E-02
ACSS1	1550.3885	-0.6847	0.1980	-3.4574	0.000545	3.6794E-02
ABCA2	433.4834	-0.9455	0.2737	-3.4551	0.00055	3.6903E-02
ENSOARG00000010767	47.4258	-0.8595	0.2491	-3.4511	0.000558	3.7245E-02
TMEM8B	29.1192	0.9770	0.2840	3.4405	0.000581	3.8513E-02
PRLR	1603.8042	0.7154	0.2083	3.4343	0.000594	3.8970E-02
SCHIP1	20.4811	1.1218	0.3265	3.4358	0.000591	3.8970E-02
EBPL	348.0731	1.0465	0.3050	3.4306	0.000602	3.9290E-02
FAM129B	1821.6593	-0.4587	0.1339	-3.4260	0.000613	3.9722E-02
MYO18A	648.7937	-0.5849	0.1708	-3.4247	0.000615	3.9722E-02
IGFBP7	28.8540	1.0970	0.3219	3.4081	0.000654	4.1997E-02
SUCLA2	363.4077	-0.5284	0.1554	-3.3998	0.000674	4.3052E-02
GADD45G	163.0742	1.0755	0.3166	3.3974	0.00068	4.3205E-02
CCNL2	327.6362	0.8133	0.2397	3.3935	0.00069	4.3390E-02
TTC14	215.8345	0.6668	0.1965	3.3933	0.00069	4.3390E-02
CTSK	108.0908	1.0083	0.2979	3.3840	0.000714	4.3650E-02
ELOVL6	120.6791	-0.9609	0.2839	-3.3853	0.000711	4.3650E-02
LTBP1	109.3335	1.0646	0.3143	3.3871	0.000706	4.3650E-02
MACC1	104.4489	0.8152	0.2409	3.3832	0.000717	4.3650E-02
SOSTDC1	10.8042	1.0776	0.3182	3.3859	0.000709	4.3650E-02
ULK1	275.1106	-0.5500	0.1624	-3.3866	0.000708	4.3650E-02
GAREM	279.7420	-0.5578	0.1651	-3.3774	0.000732	4.4129E-02
ZBED5	49.5679	0.8617	0.2551	3.3786	0.000729	4.4129E-02
SLC1A3	55.5617	0.8904	0.2640	3.3734	0.000743	4.4554E-02

PBXIP1	1039.4781	0.6702	0.1995	3.3593	0.000781	4.6654E-02
KDM1B	423.8099	-0.6234	0.1858	-3.3559	0.000791	4.6763E-02
OSBPL1A	1490.0470	-0.5081	0.1514	-3.3569	0.000788	4.6763E-02
PAK4	207.0514	-0.5666	0.1689	-3.3541	0.000796	4.6834E-02
NET1	653.3658	0.5274	0.1574	3.3501	0.000808	4.7290E-02
CRBN	303.8180	-0.5027	0.1502	-3.3475	0.000816	4.7499E-02
CCNG2	83.8098	0.8270	0.2474	3.3423	0.000831	4.7918E-02
SLC11A1	28.6446	1.0842	0.3243	3.3434	0.000828	4.7918E-02
CLGN	171.7773	1.0465	0.3135	3.3385	0.000842	4.8331E-02
ITGB6	458.4795	0.7996	0.2396	3.3373	0.000846	4.8331E-02
NELL2	18.0654	-1.0876	0.3267	-3.3289	0.000872	4.9461E-02
PNRC1	269.1266	0.7244	0.2177	3.3278	0.000875	4.9461E-02
TMEM258	114.8227	-0.7839	0.2356	-3.3269	0.000878	4.9461E-02
BHLHE41	20.5828	1.0220	0.3075	3.3234	0.000889	4.9841E-02

Supplementary File S4

Title of data: Functional enrichment analysis results of genes upregulated in the fish oil-induced milk fat depression (FO-MFD) condition.

Description of data: PDF providing the WebGestalt outputs from Gene-ontology (*GO_analysis*) and Wikipathway (*Wikipathway_analysis*) functional enrichment analyses performed with the differentially expressed genes upregulated in the FO-MFD condition.

Database:biological process Name:regulation of protein kinase activity
C=603; O=13; E=3.96; R=3.29; rawP=0.0002; adjP=0.0397 ID:GO:0045859

Index	UserID	Gene Name
1	CCNT2	cyclin T2
2	SLC11A1	solute carrier family 11 , member 1
3	CYR61	cysteine-rich, angiogenic inducer, 61
4	PRLR	prolactin receptor
5	GADD45G	growth arrest and DNA-damage-inducible, gamma
6	ATF3	activating transcription factor 3
7	CCNL1	cyclin L1
8	CCNL2	cyclin L2
9	ERRFI1	ERBB receptor feedback inhibitor 1
10	APP	amyloid beta precursor protein
11	FGFR1	fibroblast growth factor receptor 1
12	DUSP1	dual specificity phosphatase 1
13	SPRY1	sprouty homolog 1, antagonist of FGF signaling (Drosophila)

Database:biological process Name:regulation of transferase activity ID:GO:0051338
C=653; O=13; E=4.28; R=3.03; rawP=0.0003; adjP=0.0397

Index	UserID	Gene Name
1	CCNT2	cyclin T2
2	SLC11A1	solute carrier family 11 , member 1
3	CYR61	cysteine-rich, angiogenic inducer, 61
4	PRLR	prolactin receptor
5	GADD45G	growth arrest and DNA-damage-inducible, gamma
6	ATF3	activating transcription factor 3
7	CCNL1	cyclin L1
8	CCNL2	cyclin L2
9	ERRFI1	ERBB receptor feedback inhibitor 1
10	APP	amyloid beta precursor protein
11	FGFR1	fibroblast growth factor receptor 1
12	DUSP1	dual specificity phosphatase 1
13	SPRY1	sprouty homolog 1, antagonist of FGF signaling (Drosophila)

Database:biological process Name:regulation of kinase activity ID:GO:0043549
C=632; O=13; E=4.15; R=3.13; rawP=0.0002; adjP=0.0397

Index	UserID	Gene Name
1	CCNT2	cyclin T2
2	SLC11A1	solute carrier family 11 , member 1
3	CYR61	cysteine-rich, angiogenic inducer, 61
4	PRLR	prolactin receptor
5	GADD45G	growth arrest and DNA-damage-inducible, gamma
6	ATF3	activating transcription factor 3
7	CCNL1	cyclin L1
8	CCNL2	cyclin L2
9	ERRFI1	ERBB receptor feedback inhibitor 1
10	APP	amyloid beta precursor protein
11	FGFR1	fibroblast growth factor receptor 1
12	DUSP1	dual specificity phosphatase 1

13	SPRY1	sprouty homolog 1, antagonist of FGF signaling (Drosophila)

Database:molecular function Name:growth factor binding ID:GO:0019838
C=106; O=5; E=0.65; R=7.67; rawP=0.0005; adjP=0.0330

Index	UserID	Gene Name
1	LTBP1	latent transforming growth factor beta binding protein 1
2	CYR61	cysteine-rich, angiogenic inducer, 61
3	LIFR	leukemia inhibitory factor receptor alpha
4	FGFR1	fibroblast growth factor receptor 1
5	IGFBP7	insulin-like growth factor binding protein 7

Database:molecular function Name:heparin binding ID:GO:0008201
C=131; O=5; E=0.81; R=6.21; rawP=0.0013; adjP=0.0379

Index	UserID	Gene Name
1	POSTN	periostin, osteoblast specific factor
2	GPNMB	glycoprotein (transmembrane) nmb
3	CYR61	cysteine-rich, angiogenic inducer, 61
4	APP	amyloid beta precursor protein
5	FGFR1	fibroblast growth factor receptor 1

Database:molecular function Name:identical protein binding ID:GO:0042802
C=863; O=13; E=5.31; R=2.45; rawP=0.0023; adjP=0.0379

Index	UserID	Gene Name
1	AMOTL2	angiomotin like 2
2	JUN	jun proto-oncogene
3	GCA	grancalcin, EF-hand calcium binding protein
4	SLC11A1	solute carrier family 11 , member 1
5	KCNJ2	potassium inwardly-rectifying channel, subfamily J, member 2
6	PON3	paraoxonase 3
7	BHLHE41	basic helix-loop-helix family, member e41
8	SCHIP1	schwannomin interacting protein 1
9	PRLR	prolactin receptor
10	ATF3	activating transcription factor 3
11	CLDN8	claudin 8
12	APP	amyloid beta precursor protein
13	FGFR1	fibroblast growth factor receptor 1

Database:molecular function Name:protein complex binding ID:GO:0032403
C=295; O=7; E=1.81; R=3.86; rawP=0.0023; adjP=0.0379

Index	UserID	Gene Name
1	FCGR2B	Fc fragment of IgG, low affinity IIb, receptor (CD32)
2	GPNMB	glycoprotein (transmembrane) nmb
3	APBA1	amyloid beta precursor protein-binding, family A, member 1
4	CYR61	cysteine-rich, angiogenic inducer, 61
5	ITGB6	integrin, beta 6
6	FCGRT	Fc fragment of IgG, receptor, transporter, alpha
7	GNA14	guanine nucleotide binding protein (G protein), alpha 14

Database:molecular function Name:active transmembrane transporter activity
C=309; O=7; E=1.90; R=3.68; rawP=0.0029; adjP=0.0383 ID:GO:0022804

Index	UserID	Gene Name
1	SLC1A3	solute carrier family 1, member 3
2	ABCA3	ATP-binding cassette, sub-family A (ABC1), member 3
3	SLC11A1	solute carrier family 11 , member 1
4	SLC22A16	solute carrier family 22 , member 16
5	ABCC10	ATP-binding cassette, sub-family C (CFTR/MRP), member 10
6	SLC7A6	solute carrier family 7 , member 6
7	SLC22A5	solute carrier family 22 , member 5

Database:molecular function Name:glycosaminoglycan binding ID:GO:0005539
C=174; O=5; E=1.07; R=4.67; rawP=0.0044; adjP=0.0484

Index	UserID	Gene Name
1	POSTN	periostin, osteoblast specific factor
2	GPNMB	glycoprotein (transmembrane) nmb
3	CYR61	cysteine-rich, angiogenic inducer, 61
4	APP	amyloid beta precursor protein
5	FGFR1	fibroblast growth factor receptor 1

Database:cellular component Name:plasma membrane part ID:GO:0044459
C=1918; O=23; E=11.36; R=2.02; rawP=0.0007; adjP=0.0109

Index	UserID	Gene Name
1	AMOTL2	angiomotin like 2
2	SLC40A1	solute carrier family 40 , member 1
3	MPZL1	myelin protein zero-like 1
4	SLC11A1	solute carrier family 11 , member 1
5	RASAL2	RAS protein activator like 2
6	KCNJ2	potassium inwardly-rectifying channel, subfamily J, member 2
7	PTPRU	protein tyrosine phosphatase, receptor type, U
8	SLC1A3	solute carrier family 1, member 3
9	GPNMB	glycoprotein (transmembrane) nmb
10	MEGF10	multiple EGF-like-domains 10
11	APP	amyloid beta precursor protein
12	SLC7A6	solute carrier family 7 , member 6
13	FGFR1	fibroblast growth factor receptor 1
14	GNA14	guanine nucleotide binding protein (G protein), alpha 14
15	CSF2RB	colony stimulating factor 2 receptor, beta, low-affinity
16	SEMA4F	Semaphorin-4F
17	CYBA	cytochrome b-245, alpha polypeptide
18	FCGRT	Fc fragment of IgG, receptor, transporter, alpha
19	ERRFI1	ERBB receptor feedback inhibitor 1
20	ITGB6	integrin, beta 6
21	SLC31A2	solute carrier family 31 (copper transporters), member 2
22	LIFR	leukemia inhibitory factor receptor alpha
23	SLC22A5	solute carrier family 22 , member 5

Database:cellular component	Name:cell periphery	ID:GO:0071944
C=4377; O=41; E=25.92; R=1.58; rawP=0.0007; adjP=0.0109

Index	UserID	Gene Name
1	ABCA3	ATP-binding cassette, sub-family A (ABC1), member 3
2	MPZL1	myelin protein zero-like 1
3	RASAL2	RAS protein activator like 2
4	LGR4	leucine-rich repeat containing G protein-coupled receptor 4
5	SLC22A16	solute carrier family 22 , member 16
6	TNFRSF21	tumor necrosis factor receptor superfamily, member 21
7	ABCC10	ATP-binding cassette, sub-family C (CFTR/MRP), member 10
8	SLC1A3	solute carrier family 1, member 3
9	APBA1	amyloid beta precursor protein-binding, family A, member 1
10	F5	coagulation factor V (proaccelerin, labile factor)
11	APP	amyloid beta precursor protein
12	CLDN8	claudin 8
13	MEGF10	multiple EGF-like-domains 10
14	SLC7A6	solute carrier family 7 , member 6
15	CD320	CD320 molecule
16	RND1	Rho family GTPase 1
17	CSF2RB	colony stimulating factor 2 receptor, beta, low-affinity
18	SEMA4F	Semaphorin-4F
19	PRLR	prolactin receptor
20	FCGRT	Fc fragment of IgG, receptor, transporter, alpha
21	NOTCH3	notch 3
22	ERRFI1	ERBB receptor feedback inhibitor 1
23	ITGB6	integrin, beta 6
24	DSG3	desmoglein 3
25	SLC22A5	solute carrier family 22 , member 5
26	AMOTL2	angiomotin like 2
27	FCGR2B	Fc fragment of IgG, low affinity IIb, receptor (CD32)
28	SLC40A1	solute carrier family 40 , member 1
29	GCA	grancalcin, EF-hand calcium binding protein
30	SLC5A9	solute carrier family 5 , member 9
31	SLC11A1	solute carrier family 11 , member 1
32	KCNJ2	potassium inwardly-rectifying channel, subfamily J, member 2
33	PTPRU	protein tyrosine phosphatase, receptor type, U
34	GPNMB	glycoprotein (transmembrane) nmb
35	GNA14	guanine nucleotide binding protein (G protein), alpha 14
36	FGFR1	fibroblast growth factor receptor 1
37	CYBA	cytochrome b-245, alpha polypeptide
38	TMEM8B	transmembrane protein 8B
39	SLC31A2	solute carrier family 31 (copper transporters), member 2
40	LIFR	leukemia inhibitory factor receptor alpha
41	SPRY1	sprouty homolog 1, antagonist of FGF signaling (Drosophila)

Database:cellular component	Name:plasma membrane	ID:GO:0005886
C=4289; O=41; E=25.40; R=1.61; rawP=0.0005; adjP=0.0109

Index	UserID	Gene Name
1	ABCA3	ATP-binding cassette, sub-family A (ABC1), member 3
2	MPZL1	myelin protein zero-like 1
3	RASAL2	RAS protein activator like 2
4	LGR4	leucine-rich repeat containing G protein-coupled receptor 4
5	SLC22A16	solute carrier family 22 , member 16
6	TNFRSF21	tumor necrosis factor receptor superfamily, member 21
7	ABCC10	ATP-binding cassette, sub-family C (CFTR/MRP), member 10
8	SLC1A3	solute carrier family 1, member 3
9	APBA1	amyloid beta precursor protein-binding, family A, member 1
10	F5	coagulation factor V (proaccelerin, labile factor)
11	APP	amyloid beta precursor protein
12	CLDN8	claudin 8
13	MEGF10	multiple EGF-like-domains 10
14	SLC7A6	solute carrier family 7 , member 6
15	CD320	CD320 molecule
16	RND1	Rho family GTPase 1
17	CSF2RB	colony stimulating factor 2 receptor, beta, low-affinity
18	SEMA4F	Semaphorin-4F
19	PRLR	prolactin receptor
20	FCGRT	Fc fragment of IgG, receptor, transporter, alpha
21	NOTCH3	notch 3
22	ERRFI1	ERBB receptor feedback inhibitor 1
23	ITGB6	integrin, beta 6
24	DSG3	desmoglein 3
25	SLC22A5	solute carrier family 22 , member 5
26	AMOTL2	angiomotin like 2
27	FCGR2B	Fc fragment of IgG, low affinity IIb, receptor (CD32)
28	SLC40A1	solute carrier family 40 , member 1
29	GCA	grancalcin, EF-hand calcium binding protein
30	SLC5A9	solute carrier family 5 , member 9
31	SLC11A1	solute carrier family 11 , member 1
32	KCNJ2	potassium inwardly-rectifying channel, subfamily J, member 2
33	PTPRU	protein tyrosine phosphatase, receptor type, U
34	GPNMB	glycoprotein (transmembrane) nmb
35	GNA14	guanine nucleotide binding protein (G protein), alpha 14
36	FGFR1	fibroblast growth factor receptor 1
37	CYBA	cytochrome b-245, alpha polypeptide
38	TMEM8B	transmembrane protein 8B
39	SLC31A2	solute carrier family 31 (copper transporters), member 2
40	LIFR	leukemia inhibitory factor receptor alpha
41	SPRY1	sprouty homolog 1, antagonist of FGF signaling (Drosophila)

Database:cellular component Name:membrane part ID:GO:0044425
C=6154; O=54; E=36.45; R=1.48; rawP=0.0003; adjP=0.0109

Index	UserID	Gene Name
1	TCTN3	tectonic family member 3
2	ABCA3	ATP-binding cassette, sub-family A (ABC1), member 3
3	MPZL1	myelin protein zero-like 1
4	FAM189A2	family with sequence similarity 189, member A2

5	RASAL2	RAS protein activator like 2
6	LGR4	leucine-rich repeat containing G protein-coupled receptor 4
7	CLGN	calmegin
8	HS3ST1	heparan sulfate (glucosamine) 3-O-sulfotransferase 1
9	SLC22A16	solute carrier family 22 , member 16
10	TNFRSF21	tumor necrosis factor receptor superfamily, member 21
11	ABCC10	ATP-binding cassette, sub-family C (CFTR/MRP), member 10
12	SLC1A3	solute carrier family 1, member 3
13	CRELD1	cysteine-rich with EGF-like domains 1
14	APP	amyloid beta precursor protein
15	CYP39A1	cytochrome P450, family 39, subfamily A, polypeptide 1
16	MEGF10	multiple EGF-like-domains 10
17	CLDN8	claudin 8
18	GPX8	glutathione peroxidase 8 (putative)
19	SLC7A6	solute carrier family 7 , member 6
20	CD320	CD320 molecule
21	CSF2RB	colony stimulating factor 2 receptor, beta, low-affinity
22	SEMA4F	Semaphorin-4F
23	PRLR	prolactin receptor
24	FCGRT	Fc fragment of IgG, receptor, transporter, alpha
25	NOTCH3	notch 3
26	ERRFI1	ERBB receptor feedback inhibitor 1
27	SCARA3	scavenger receptor class A, member 3
28	ITGB6	integrin, beta 6
29	DSG3	desmoglein 3
30	SLC22A5	solute carrier family 22 , member 5
31	AMOTL2	angiomotin like 2
32	FCGR2B	Fc fragment of IgG, low affinity IIb, receptor (CD32)
33	SLC40A1	solute carrier family 40 , member 1
34	SLC5A9	solute carrier family 5 , member 9
35	SLC11A1	solute carrier family 11 , member 1
36	EBPL	emopamil binding protein-like
37	KCNJ2	potassium inwardly-rectifying channel, subfamily J, member 2
38	PTPRU	protein tyrosine phosphatase, receptor type, U
39	CYP7B1	cytochrome P450, family 7, subfamily B, polypeptide 1
40	PPT1	palmitoyl-protein thioesterase 1
41	MCL1	myeloid cell leukemia sequence 1 (BCL2-related)
42	GPNMB	glycoprotein (transmembrane) nmb
43	LRIG3	leucine-rich repeats and immunoglobulin-like domains 3
44	PTGFRN	prostaglandin F2 receptor negative regulator
45	GNA14	guanine nucleotide binding protein (G protein), alpha 14
46	FGFR1	fibroblast growth factor receptor 1
47	RETSAT	retinol saturase (all-trans-retinol 13,14-reductase)
48	TMEM168	transmembrane protein 168
49	CYBA	cytochrome b-245, alpha polypeptide
50	ARL6IP5	ADP-ribosylation-like factor 6 interacting protein 5
51	C5orf42	chromosome 5 open reading frame 42
52	TMEM8B	transmembrane protein 8B
53	SLC31A2	solute carrier family 31 (copper transporters), member 2
54	LIFR	leukemia inhibitory factor receptor alpha

Database:cellular component Name:intrinsic to membrane ID:GO:0031224
C=5437; O=47; E=32.20; R=1.46; rawP=0.0014; adjP=0.0174

Index	UserID	Gene Name
1	TCTN3	tectonic family member 3
2	ABCA3	ATP-binding cassette, sub-family A (ABC1), member 3
3	MPZL1	myelin protein zero-like 1
4	FAM189A2	family with sequence similarity 189, member A2
5	RASAL2	RAS protein activator like 2
6	LGR4	leucine-rich repeat containing G protein-coupled receptor 4
7	CLGN	calmegin
8	HS3ST1	heparan sulfate (glucosamine) 3-O-sulfotransferase 1
9	SLC22A16	solute carrier family 22 , member 16
10	TNFRSF21	tumor necrosis factor receptor superfamily, member 21
11	ABCC10	ATP-binding cassette, sub-family C (CFTR/MRP), member 10
12	SLC1A3	solute carrier family 1, member 3
13	CRELD1	cysteine-rich with EGF-like domains 1
14	APP	amyloid beta precursor protein
15	MEGF10	multiple EGF-like-domains 10
16	CLDN8	claudin 8
17	GPX8	glutathione peroxidase 8 (putative)
18	SLC7A6	solute carrier family 7 , member 6
19	CD320	CD320 molecule
20	CSF2RB	colony stimulating factor 2 receptor, beta, low-affinity
21	SEMA4F	Semaphorin-4F
22	PRLR	prolactin receptor
23	FCGRT	Fc fragment of IgG, receptor, transporter, alpha
24	NOTCH3	notch 3
25	SCARA3	scavenger receptor class A, member 3
26	ITGB6	integrin, beta 6
27	DSG3	desmoglein 3
28	SLC22A5	solute carrier family 22 , member 5
29	FCGR2B	Fc fragment of IgG, low affinity IIb, receptor (CD32)
30	SLC40A1	solute carrier family 40 , member 1
31	SLC5A9	solute carrier family 5 , member 9
32	SLC11A1	solute carrier family 11 , member 1
33	EBPL	emopamil binding protein-like
34	KCNJ2	potassium inwardly-rectifying channel, subfamily J, member 2
35	PTPRU	protein tyrosine phosphatase, receptor type, U
36	MCL1	myeloid cell leukemia sequence 1 (BCL2-related)
37	GPNMB	glycoprotein (transmembrane) nmb
38	LRIG3	leucine-rich repeats and immunoglobulin-like domains 3
39	PTGFRN	prostaglandin F2 receptor negative regulator
40	FGFR1	fibroblast growth factor receptor 1
41	TMEM168	transmembrane protein 168
42	CYBA	cytochrome b-245, alpha polypeptide
43	ARL6IP5	ADP-ribosylation-like factor 6 interacting protein 5
44	C5orf42	chromosome 5 open reading frame 42
45	TMEM8B	transmembrane protein 8B

46	SLC31A2	solute carrier family 31 (copper transporters), member 2
47	LIFR	leukemia inhibitory factor receptor alpha

Database:cellular component Name:integral to membrane ID:GO:0016021
C=5321; O=46; E=31.51; R=1.46; rawP=0.0017; adjP=0.0176

Index	UserID	Gene Name
1	TCTN3	tectonic family member 3
2	ABCA3	ATP-binding cassette, sub-family A (ABC1), member 3
3	MPZL1	myelin protein zero-like 1
4	FAM189A2	family with sequence similarity 189, member A2
5	LGR4	leucine-rich repeat containing G protein-coupled receptor 4
6	CLGN	calmegin
7	HS3ST1	heparan sulfate (glucosamine) 3-O-sulfotransferase 1
8	SLC22A16	solute carrier family 22 , member 16
9	TNFRSF21	tumor necrosis factor receptor superfamily, member 21
10	ABCC10	ATP-binding cassette, sub-family C (CFTR/MRP), member 10
11	SLC1A3	solute carrier family 1, member 3
12	CRELD1	cysteine-rich with EGF-like domains 1
13	APP	amyloid beta precursor protein
14	MEGF10	multiple EGF-like-domains 10
15	CLDN8	claudin 8
16	GPX8	glutathione peroxidase 8 (putative)
17	SLC7A6	solute carrier family 7 , member 6
18	CD320	CD320 molecule
19	CSF2RB	colony stimulating factor 2 receptor, beta, low-affinity
20	SEMA4F	Semaphorin-4F
21	PRLR	prolactin receptor
22	FCGRT	Fc fragment of IgG, receptor, transporter, alpha
23	NOTCH3	notch 3
24	SCARA3	scavenger receptor class A, member 3
25	ITGB6	integrin, beta 6
26	DSG3	desmoglein 3
27	SLC22A5	solute carrier family 22 , member 5
28	FCGR2B	Fc fragment of IgG, low affinity IIb, receptor (CD32)
29	SLC40A1	solute carrier family 40 , member 1
30	SLC5A9	solute carrier family 5 , member 9
31	SLC11A1	solute carrier family 11 , member 1
32	EBPL	emopamil binding protein-like
33	KCNJ2	potassium inwardly-rectifying channel, subfamily J, member 2
34	PTPRU	protein tyrosine phosphatase, receptor type, U
35	MCL1	myeloid cell leukemia sequence 1 (BCL2-related)
36	GPNMB	glycoprotein (transmembrane) nmb
37	LRIG3	leucine-rich repeats and immunoglobulin-like domains 3
38	PTGFRN	prostaglandin F2 receptor negative regulator
39	FGFR1	fibroblast growth factor receptor 1
40	TMEM168	transmembrane protein 168
41	CYBA	cytochrome b-245, alpha polypeptide
42	ARL6IP5	ADP-ribosylation-like factor 6 interacting protein 5
43	C5orf42	chromosome 5 open reading frame 42

44	TMEM8B	transmembrane protein 8B
45	SLC31A2	solute carrier family 31 (copper transporters), member 2
46	LIFR	leukemia inhibitory factor receptor alpha

Database:cellular component Name:intrinsic to plasma membrane ID:GO:0031226
C=1264; O=16; E=7.49; R=2.14; rawP=0.0030; adjP=0.0266

Index	UserID	Gene Name
1	SLC40A1	solute carrier family 40 , member 1
2	MPZL1	myelin protein zero-like 1
3	SLC11A1	solute carrier family 11 , member 1
4	RASAL2	RAS protein activator like 2
5	KCNJ2	potassium inwardly-rectifying channel, subfamily J, member 2
6	PTPRU	protein tyrosine phosphatase, receptor type, U
7	GPNMB	glycoprotein (transmembrane) nmb
8	APP	amyloid beta precursor protein
9	SLC7A6	solute carrier family 7 , member 6
10	FGFR1	fibroblast growth factor receptor 1
11	CSF2RB	colony stimulating factor 2 receptor, beta, low-affinity
12	CYBA	cytochrome b-245, alpha polypeptide
13	SEMA4F	Semaphorin-4F
14	SLC31A2	solute carrier family 31 (copper transporters), member 2
15	ITGB6	integrin, beta 6
16	LIFR	leukemia inhibitory factor receptor alpha

Database:cellular component Name:membrane ID:GO:0016020
C=7811; O=60; E=46.26; R=1.30; rawP=0.0040; adjP=0.0310

Index	UserID	Gene Name
1	TCTN3	tectonic family member 3
2	ABCA3	ATP-binding cassette, sub-family A (ABC1), member 3
3	MPZL1	myelin protein zero-like 1
4	FAM189A2	family with sequence similarity 189, member A2
5	RASAL2	RAS protein activator like 2
6	LGR4	leucine-rich repeat containing G protein-coupled receptor 4
7	CLGN	calmegin
8	HS3ST1	heparan sulfate (glucosamine) 3-O-sulfotransferase 1
9	SLC22A16	solute carrier family 22 , member 16
10	TNFRSF21	tumor necrosis factor receptor superfamily, member 21
11	ABCC10	ATP-binding cassette, sub-family C (CFTR/MRP), member 10
12	SLC1A3	solute carrier family 1, member 3
13	APBA1	amyloid beta precursor protein-binding, family A, member 1
14	CRELD1	cysteine-rich with EGF-like domains 1
15	F5	coagulation factor V (proaccelerin, labile factor)
16	APP	amyloid beta precursor protein
17	CYP39A1	cytochrome P450, family 39, subfamily A, polypeptide 1
18	MEGF10	multiple EGF-like-domains 10
19	CLDN8	claudin 8
20	GPX8	glutathione peroxidase 8 (putative)
21	SLC7A6	solute carrier family 7 , member 6
22	CD320	CD320 molecule

23	RND1	Rho family GTPase 1
24	CSF2RB	colony stimulating factor 2 receptor, beta, low-affinity
25	SEMA4F	Semaphorin-4F
26	PRLR	prolactin receptor
27	FCGRT	Fc fragment of IgG, receptor, transporter, alpha
28	NOTCH3	notch 3
29	ERRFI1	ERBB receptor feedback inhibitor 1
30	SCARA3	scavenger receptor class A, member 3
31	ITGB6	integrin, beta 6
32	DSG3	desmoglein 3
33	SLC22A5	solute carrier family 22 , member 5
34	AMOTL2	angiomotin like 2
35	FCGR2B	Fc fragment of IgG, low affinity IIb, receptor (CD32)
36	SLC40A1	solute carrier family 40 , member 1
37	GCA	grancalcin, EF-hand calcium binding protein
38	SLC5A9	solute carrier family 5 , member 9
39	SLC11A1	solute carrier family 11 , member 1
40	EBPL	emopamil binding protein-like
41	KCNJ2	potassium inwardly-rectifying channel, subfamily J, member 2
42	PTPRU	protein tyrosine phosphatase, receptor type, U
43	CYP7B1	cytochrome P450, family 7, subfamily B, polypeptide 1
44	PPT1	palmitoyl-protein thioesterase 1
45	MCL1	myeloid cell leukemia sequence 1 (BCL2-related)
46	EXOG	endo/exonuclease (5'-3'), endonuclease G-like
47	GPNMB	glycoprotein (transmembrane) nmb
48	LRIG3	leucine-rich repeats and immunoglobulin-like domains 3
49	PTGFRN	prostaglandin F2 receptor negative regulator
50	GNA14	guanine nucleotide binding protein (G protein), alpha 14
51	FGFR1	fibroblast growth factor receptor 1
52	RETSAT	retinol saturase (all-trans-retinol 13,14-reductase)
53	TMEM168	transmembrane protein 168
54	CYBA	cytochrome b-245, alpha polypeptide
55	ARL6IP5	ADP-ribosylation-like factor 6 interacting protein 5
56	C5orf42	chromosome 5 open reading frame 42
57	TMEM8B	transmembrane protein 8B
58	SLC31A2	solute carrier family 31 (copper transporters), member 2
59	LIFR	leukemia inhibitory factor receptor alpha
60	SPRY1	sprouty homolog 1, antagonist of FGF signaling (Drosophila)

Database:cellular component Name:integral to plasma membrane ID:GO:0005887
C=1220; O=15; E=7.23; R=2.08; rawP=0.0053; adjP=0.0365

Index	UserID	Gene Name
1	CSF2RB	colony stimulating factor 2 receptor, beta, low-affinity
2	SLC40A1	solute carrier family 40 , member 1
3	SEMA4F	Semaphorin-4F
4	MPZL1	myelin protein zero-like 1
5	CYBA	cytochrome b-245, alpha polypeptide
6	SLC11A1	solute carrier family 11 , member 1
7	KCNJ2	potassium inwardly-rectifying channel, subfamily J, member 2

8	PTPRU	protein tyrosine phosphatase, receptor type, U
9	GPNMB	glycoprotein (transmembrane) nmb
10	ITGB6	integrin, beta 6
11	SLC31A2	solute carrier family 31 (copper transporters), member 2
12	APP	amyloid beta precursor protein
13	LIFR	leukemia inhibitory factor receptor alpha
14	SLC7A6	solute carrier family 7 , member 6
15	FGFR1	fibroblast growth factor receptor 1

Database:Wikipathways pathway Name:TGF beta Signaling Pathway ID:WP366
C=148; O=5; E=0.37; R=13.62; rawP=3.57e-05; adjP=3.57e-05

Index	UserID	Gene Name
1	JUN	jun proto-oncogene
2	KLF11	Kruppel-like factor 11
3	ATF3	activating transcription factor 3
4	KLF6	Kruppel-like factor 6
5	FOSB	FBJ murine osteosarcoma viral oncogene homolog B

Database:Wikipathways pathway Name:Adipogenesis ID:WP236
C=130; O=5; E=0.32; R=15.50; rawP=1.91e-05; adjP=3.57e-05

Index	UserID	Gene Name
1	KLF6	Kruppel-like factor 6
2	LIFR	leukemia inhibitory factor receptor alpha
3	EGR2	early growth response 2
4	PRLR	prolactin receptor
5	BMP1	bone morphogenetic protein 1

Supplementary File S5

Title of data: Functional enrichment analysis results of genes downregulated in the fish oil-induced milk fat depression (FO-MFD) condition.

Description of data: PDF providing the WebGestalt outputs from Gene-ontology (*GO_analysis*) and Wikipathway (*Wikipathway_analysis*) functional enrichment analyses performed with the differentially expressed genes identified as downregulated in the FO-MFD condition.

Database:biological process Name:lipid biosynthetic process ID:GO:0008610
C=544; O=15; E=2.64; R=5.68; rawP=3.96e-08; adjP=8.43e-06

Index	UserID	Gene Name
1	FADS2	fatty acid desaturase 2
2	LSS	lanosterol synthase (2,3-oxidosqualene-lanosterol cyclase)
3	HMGCS1	3-hydroxy-3-methylglutaryl-CoA synthase 1 (soluble)
4	ACSL1	acyl-CoA synthetase long-chain family member 1
5	AGPAT2	1-acylglycerol-3-phosphate O-acyltransferase 2
6	ACSS2	acyl-CoA synthetase short-chain family member 2
7	ELOVL6	ELOVL fatty acid elongase 6
8	G6PD	glucose-6-phosphate dehydrogenase
9	MVD	mevalonate (diphospho) decarboxylase
10	FDPS	farnesyl diphosphate synthase
11	PCYT2	phosphate cytidylyltransferase 2, ethanolamine
12	PNPLA3	patatin-like phospholipase domain containing 3
13	ACSS1	acyl-CoA synthetase short-chain family member 1
14	LPIN1	lipin 1
15	ACACA	acetyl-CoA carboxylase alpha

Database:biological process Name:lipid metabolic process ID:GO:0006629
C=1141; O=21; E=5.54; R=3.79; rawP=5.64e-08; adjP=8.43e-06

Index	UserID	Gene Name
1	FADS2	fatty acid desaturase 2
2	ACSL1	acyl-CoA synthetase long-chain family member 1
3	AACS	acetoacetyl-CoA synthetase
4	ELOVL6	ELOVL fatty acid elongase 6
5	MVD	mevalonate (diphospho) decarboxylase
6	LPIN1	lipin 1
7	OSBPL1A	oxysterol binding protein-like 1A
8	LSS	lanosterol synthase (2,3-oxidosqualene-lanosterol cyclase)
9	AZGP1	alpha-2-glycoprotein 1, zinc-binding
10	HMGCS1	3-hydroxy-3-methylglutaryl-CoA synthase 1 (soluble)
11	PAFAH1B3	platelet-activating factor acetylhydrolase 1b, catalytic subunit 3
12	AGPAT2	1-acylglycerol-3-phosphate O-acyltransferase 2
13	ACSS2	acyl-CoA synthetase short-chain family member 2
14	G6PD	glucose-6-phosphate dehydrogenase
15	FDPS	farnesyl diphosphate synthase
16	ABCA2	ATP-binding cassette, sub-family A (ABC1), member 2
17	PCYT2	phosphate cytidylyltransferase 2, ethanolamine
18	PNPLA3	patatin-like phospholipase domain containing 3
19	YWHAH	tyrosine 3-monooxygenase
20	ACSS1	acyl-CoA synthetase short-chain family member 1
21	ACACA	acetyl-CoA carboxylase alpha

Database:biological process Name:triglyceride biosynthetic process ID:GO:0019432
C=56; O=6; E=0.27; R=22.08; rawP=2.82e-07; adjP=1.30e-05

Index	UserID	Gene Name
1	ACSL1	acyl-CoA synthetase long-chain family member 1
2	PNPLA3	patatin-like phospholipase domain containing 3

3	AGPAT2	1-acylglycerol-3-phosphate O-acyltransferase 2
4	ELOVL6	ELOVL fatty acid elongase 6
5	ACACA	acetyl-CoA carboxylase alpha
6	LPIN1	lipin 1

Database:biological process Name:thioester metabolic process ID:GO:0035383
C=93; O=7; E=0.45; R=15.51; rawP=3.19e-07; adjP=1.30e-05

Index	UserID	Gene Name
1	SUCLA2	succinate-CoA ligase, ADP-forming, beta subunit
2	ACSL1	acyl-CoA synthetase long-chain family member 1
3	ACSS1	acyl-CoA synthetase short-chain family member 1
4	ACSS2	acyl-CoA synthetase short-chain family member 2
5	ELOVL6	ELOVL fatty acid elongase 6
6	MVD	mevalonate (diphospho) decarboxylase
7	ACACA	acetyl-CoA carboxylase alpha

Database:biological process Name:single-organism biosynthetic process ID:GO:0044711
C=437; O=13; E=2.12; R=6.13; rawP=1.51e-07; adjP=1.30e-05

Index	UserID	Gene Name
1	SUCLA2	succinate-CoA ligase, ADP-forming, beta subunit
2	FADS2	fatty acid desaturase 2
3	LSS	lanosterol synthase (2,3-oxidosqualene-lanosterol cyclase)
4	HMGCS1	3-hydroxy-3-methylglutaryl-CoA synthase 1 (soluble)
5	ELOVL6	ELOVL fatty acid elongase 6
6	G6PD	glucose-6-phosphate dehydrogenase
7	MVD	mevalonate (diphospho) decarboxylase
8	FDPS	farnesyl diphosphate synthase
9	ASS1	argininosuccinate synthase 1
10	TYR	tyrosinase (oculocutaneous albinism IA)
11	ACSS1	acyl-CoA synthetase short-chain family member 1
12	LPIN1	lipin 1
13	ACACA	acetyl-CoA carboxylase alpha

Database:biological process Name:acylglycerol biosynthetic process ID:GO:0046463
C=58; O=6; E=0.28; R=21.32; rawP=3.49e-07; adjP=1.30e-05

Index	UserID	Gene Name
1	ACSL1	acyl-CoA synthetase long-chain family member 1
2	PNPLA3	patatin-like phospholipase domain containing 3
3	AGPAT2	1-acylglycerol-3-phosphate O-acyltransferase 2
4	ELOVL6	ELOVL fatty acid elongase 6
5	ACACA	acetyl-CoA carboxylase alpha
6	LPIN1	lipin 1

Database:biological process Name:acyl-CoA metabolic process ID:GO:0006637
C=93; O=7; E=0.45; R=15.51; rawP=3.19e-07; adjP=1.30e-05

Index	UserID	Gene Name

1	SUCLA2	succinate-CoA ligase, ADP-forming, beta subunit
2	ACSL1	acyl-CoA synthetase long-chain family member 1
3	ACSS1	acyl-CoA synthetase short-chain family member 1
4	ACSS2	acyl-CoA synthetase short-chain family member 2
5	ELOVL6	ELOVL fatty acid elongase 6
6	MVD	mevalonate (diphospho) decarboxylase
7	ACACA	acetyl-CoA carboxylase alpha

Database:biological process Name:neutral lipid biosynthetic process ID:GO:0046460
C=58; O=6; E=0.28; R=21.32; rawP=3.49e-07; adjP=1.30e-05

Index	UserID	Gene Name
1	ACSL1	acyl-CoA synthetase long-chain family member 1
2	PNPLA3	patatin-like phospholipase domain containing 3
3	AGPAT2	1-acylglycerol-3-phosphate O-acyltransferase 2
4	ELOVL6	ELOVL fatty acid elongase 6
5	ACACA	acetyl-CoA carboxylase alpha
6	LPIN1	lipin 1

Database:biological process Name:thioester biosynthetic process ID:GO:0035384
C=36; O=5; E=0.17; R=28.62; rawP=7.83e-07; adjP=2.28e-05

Index	UserID	Gene Name
1	ACSL1	acyl-CoA synthetase long-chain family member 1
2	ACSS1	acyl-CoA synthetase short-chain family member 1
3	ACSS2	acyl-CoA synthetase short-chain family member 2
4	ELOVL6	ELOVL fatty acid elongase 6
5	ACACA	acetyl-CoA carboxylase alpha

Database:biological process Name:acyl-CoA biosynthetic process ID:GO:0071616
C=36; O=5; E=0.17; R=28.62; rawP=7.83e-07; adjP=2.28e-05

Index	UserID	Gene Name
1	ACSL1	acyl-CoA synthetase long-chain family member 1
2	ACSS1	acyl-CoA synthetase short-chain family member 1
3	ACSS2	acyl-CoA synthetase short-chain family member 2
4	ELOVL6	ELOVL fatty acid elongase 6
5	ACACA	acetyl-CoA carboxylase alpha

Database:biological process Name:small molecule biosynthetic process ID:GO:0044283
C=425; O=12; E=2.06; R=5.82; rawP=8.39e-07; adjP=2.28e-05

Index	UserID	Gene Name
1	SUCLA2	succinate-CoA ligase, ADP-forming, beta subunit
2	FADS2	fatty acid desaturase 2
3	LSS	lanosterol synthase (2,3-oxidosqualene-lanosterol cyclase)
4	HMGCS1	3-hydroxy-3-methylglutaryl-CoA synthase 1 (soluble)
5	ELOVL6	ELOVL fatty acid elongase 6
6	G6PD	glucose-6-phosphate dehydrogenase
7	MVD	mevalonate (diphospho) decarboxylase
8	FDPS	farnesyl diphosphate synthase

9	ASS1	argininosuccinate synthase 1
10	ACSS1	acyl-CoA synthetase short-chain family member 1
11	LPIN1	lipin 1
12	ACACA	acetyl-CoA carboxylase alpha

Database:biological process Name:cholesterol biosynthetic process ID:GO:0006695
C=44; O=5; E=0.21; R=23.42; rawP=2.19e-06; adjP=5.04e-05

Index	UserID	Gene Name
1	LSS	lanosterol synthase (2,3-oxidosqualene-lanosterol cyclase)
2	HMGCS1	3-hydroxy-3-methylglutaryl-CoA synthase 1 (soluble)
3	G6PD	glucose-6-phosphate dehydrogenase
4	MVD	mevalonate (diphospho) decarboxylase
5	FDPS	farnesyl diphosphate synthase

Database:biological process Name:small molecule metabolic process ID:GO:0044281
C=2515; O=29; E=12.21; R=2.38; rawP=2.16e-06; adjP=5.04e-05

Index	UserID	Gene Name
1	SUCLA2	succinate-CoA ligase, ADP-forming, beta subunit
2	FADS2	fatty acid desaturase 2
3	SDSL	serine dehydratase-like
4	ATF4	activating transcription factor 4 (tax-responsive enhancer element B67)
5	ACSL1	acyl-CoA synthetase long-chain family member 1
6	AACS	acetoacetyl-CoA synthetase
7	ELOVL6	ELOVL fatty acid elongase 6
8	MVD	mevalonate (diphospho) decarboxylase
9	FGD2	FYVE, RhoGEF and PH domain containing 2
10	ASS1	argininosuccinate synthase 1
11	TYR	tyrosinase (oculocutaneous albinism IA)
12	PDE8A	phosphodiesterase 8A
13	SMAP2	small ArfGAP2
14	LPIN1	lipin 1
15	OSBPL1A	oxysterol binding protein-like 1A
16	LSS	lanosterol synthase (2,3-oxidosqualene-lanosterol cyclase)
17	HMGCS1	3-hydroxy-3-methylglutaryl-CoA synthase 1 (soluble)
18	ASAP2	ArfGAP with SH3 domain, ankyrin repeat and PH domain 2
19	AGPAT2	1-acylglycerol-3-phosphate O-acyltransferase 2
20	ACSS2	acyl-CoA synthetase short-chain family member 2
21	G6PD	glucose-6-phosphate dehydrogenase
22	FDPS	farnesyl diphosphate synthase
23	ALDOA	aldolase A, fructose-bisphosphate
24	ABCA2	ATP-binding cassette, sub-family A (ABC1), member 2
25	PCYT2	phosphate cytidylyltransferase 2, ethanolamine
26	PCK2	phosphoenolpyruvate carboxykinase 2 (mitochondrial)
27	PNPLA3	patatin-like phospholipase domain containing 3
28	ACSS1	acyl-CoA synthetase short-chain family member 1
29	ACACA	acetyl-CoA carboxylase alpha

Database:biological process Name:sterol biosynthetic process ID:GO:0016126
C=50; O=5; E=0.24; R=20.61; rawP=4.17e-06; adjP=8.91e-05

Index	UserID	Gene Name
1	LSS	lanosterol synthase (2,3-oxidosqualene-lanosterol cyclase)
2	HMGCS1	3-hydroxy-3-methylglutaryl-CoA synthase 1 (soluble)
3	G6PD	glucose-6-phosphate dehydrogenase
4	MVD	mevalonate (diphospho) decarboxylase
5	FDPS	farnesyl diphosphate synthase

Database:biological process Name:acetyl-CoA metabolic process ID:GO:0006084
C=52; O=5; E=0.25; R=19.81; rawP=5.08e-06; adjP=0.0001

Index	UserID	Gene Name
1	SUCLA2	succinate-CoA ligase, ADP-forming, beta subunit
2	ACSS1	acyl-CoA synthetase short-chain family member 1
3	ACSS2	acyl-CoA synthetase short-chain family member 2
4	MVD	mevalonate (diphospho) decarboxylase
5	ACACA	acetyl-CoA carboxylase alpha

Database:biological process Name:coenzyme metabolic process ID:GO:0006732
C=215; O=8; E=1.04; R=7.67; rawP=9.18e-06; adjP=0.0002

Index	UserID	Gene Name
1	SUCLA2	succinate-CoA ligase, ADP-forming, beta subunit
2	ACSL1	acyl-CoA synthetase long-chain family member 1
3	ACSS2	acyl-CoA synthetase short-chain family member 2
4	ELOVL6	ELOVL fatty acid elongase 6
5	G6PD	glucose-6-phosphate dehydrogenase
6	MVD	mevalonate (diphospho) decarboxylase
7	ACSS1	acyl-CoA synthetase short-chain family member 1
8	ACACA	acetyl-CoA carboxylase alpha

Database:biological process Name:triglyceride metabolic process ID:GO:0006641
C=104; O=6; E=0.50; R=11.89; rawP=1.10e-05; adjP=0.0002

Index	UserID	Gene Name
1	ACSL1	acyl-CoA synthetase long-chain family member 1
2	PNPLA3	patatin-like phospholipase domain containing 3
3	AGPAT2	1-acylglycerol-3-phosphate O-acyltransferase 2
4	ELOVL6	ELOVL fatty acid elongase 6
5	ACACA	acetyl-CoA carboxylase alpha
6	LPIN1	lipin 1

Database:biological process Name:acylglycerol metabolic process ID:GO:0006639
C=109; O=6; E=0.53; R=11.34; rawP=1.44e-05; adjP=0.0002

Index	UserID	Gene Name
1	ACSL1	acyl-CoA synthetase long-chain family member 1
2	PNPLA3	patatin-like phospholipase domain containing 3
3	AGPAT2	1-acylglycerol-3-phosphate O-acyltransferase 2
4	ELOVL6	ELOVL fatty acid elongase 6

5	ACACA	acetyl-CoA carboxylase alpha
6	LPIN1	lipin 1

Database:biological process Name:neutral lipid metabolic process ID:GO:0006638
C=110; O=6; E=0.53; R=11.24; rawP=1.52e-05; adjP=0.0002

Index	UserID	Gene Name
1	ACSL1	acyl-CoA synthetase long-chain family member 1
2	PNPLA3	patatin-like phospholipase domain containing 3
3	AGPAT2	1-acylglycerol-3-phosphate O-acyltransferase 2
4	ELOVL6	ELOVL fatty acid elongase 6
5	ACACA	acetyl-CoA carboxylase alpha
6	LPIN1	lipin 1

Database:biological process Name:carboxylic acid metabolic process ID:GO:0019752
C=849; O=15; E=4.12; R=3.64; rawP=1.08e-05; adjP=0.0002

Index	UserID	Gene Name
1	SUCLA2	succinate-CoA ligase, ADP-forming, beta subunit
2	FADS2	fatty acid desaturase 2
3	SDSL	serine dehydratase-like
4	ATF4	activating transcription factor 4 (tax-responsive enhancer element B67)
5	ACSL1	acyl-CoA synthetase long-chain family member 1
6	ACSS2	acyl-CoA synthetase short-chain family member 2
7	AACS	acetoacetyl-CoA synthetase
8	ELOVL6	ELOVL fatty acid elongase 6
9	G6PD	glucose-6-phosphate dehydrogenase
10	ASS1	argininosuccinate synthase 1
11	TYR	tyrosinase (oculocutaneous albinism IA)
12	PCK2	phosphoenolpyruvate carboxykinase 2 (mitochondrial)
13	ACSS1	acyl-CoA synthetase short-chain family member 1
14	LPIN1	lipin 1
15	ACACA	acetyl-CoA carboxylase alpha

Database:biological process Name:cholesterol metabolic process ID:GO:0008203
C=115; O=6; E=0.56; R=10.75; rawP=1.95e-05; adjP=0.0003

Index	UserID	Gene Name
1	LSS	lanosterol synthase (2,3-oxidosqualene-lanosterol cyclase)
2	HMGCS1	3-hydroxy-3-methylglutaryl-CoA synthase 1 (soluble)
3	G6PD	glucose-6-phosphate dehydrogenase
4	MVD	mevalonate (diphospho) decarboxylase
5	FDPS	farnesyl diphosphate synthase
6	OSBPL1A	oxysterol binding protein-like 1A

Database:biological process Name:alcohol metabolic process ID:GO:0006066
C=321; O=9; E=1.56; R=5.78; rawP=2.37e-05; adjP=0.0003

Index	UserID	Gene Name
1	LSS	lanosterol synthase (2,3-oxidosqualene-lanosterol cyclase)

2	HMGCS1	3-hydroxy-3-methylglutaryl-CoA synthase 1 (soluble)
3	ACSS2	acyl-CoA synthetase short-chain family member 2
4	G6PD	glucose-6-phosphate dehydrogenase
5	MVD	mevalonate (diphospho) decarboxylase
6	FDPS	farnesyl diphosphate synthase
7	ACSS1	acyl-CoA synthetase short-chain family member 1
8	LPIN1	lipin 1
9	OSBPL1A	oxysterol binding protein-like 1A

Database:biological process Name:sterol metabolic process ID:GO:0016125
C=122; O=6; E=0.59; R=10.13; rawP=2.73e-05; adjP=0.0004

Index	UserID	Gene Name
1	LSS	lanosterol synthase (2,3-oxidosqualene-lanosterol cyclase)
2	HMGCS1	3-hydroxy-3-methylglutaryl-CoA synthase 1 (soluble)
3	G6PD	glucose-6-phosphate dehydrogenase
4	MVD	mevalonate (diphospho) decarboxylase
5	FDPS	farnesyl diphosphate synthase
6	OSBPL1A	oxysterol binding protein-like 1A

Database:biological process Name:cofactor metabolic process ID:GO:0051186
C=257; O=8; E=1.25; R=6.41; rawP=3.33e-05; adjP=0.0004

Index	UserID	Gene Name
1	SUCLA2	succinate-CoA ligase, ADP-forming, beta subunit
2	ACSL1	acyl-CoA synthetase long-chain family member 1
3	ACSS2	acyl-CoA synthetase short-chain family member 2
4	ELOVL6	ELOVL fatty acid elongase 6
5	G6PD	glucose-6-phosphate dehydrogenase
6	MVD	mevalonate (diphospho) decarboxylase
7	ACSS1	acyl-CoA synthetase short-chain family member 1
8	ACACA	acetyl-CoA carboxylase alpha

Database:biological process Name:cofactor biosynthetic process ID:GO:0051188
C=133; O=6; E=0.65; R=9.30; rawP=4.45e-05; adjP=0.0005

Index	UserID	Gene Name
1	SUCLA2	succinate-CoA ligase, ADP-forming, beta subunit
2	ACSL1	acyl-CoA synthetase long-chain family member 1
3	ACSS1	acyl-CoA synthetase short-chain family member 1
4	ACSS2	acyl-CoA synthetase short-chain family member 2
5	ELOVL6	ELOVL fatty acid elongase 6
6	ACACA	acetyl-CoA carboxylase alpha

Database:biological process Name:oxoacid metabolic process ID:GO:0043436
C=956; O=15; E=4.64; R=3.23; rawP=4.37e-05; adjP=0.0005

Index	UserID	Gene Name
1	SUCLA2	succinate-CoA ligase, ADP-forming, beta subunit
2	FADS2	fatty acid desaturase 2
3	SDSL	serine dehydratase-like

4	ATF4	activating transcription factor 4 (tax-responsive enhancer element B67)
5	ACSL1	acyl-CoA synthetase long-chain family member 1
6	ACSS2	acyl-CoA synthetase short-chain family member 2
7	AACS	acetoacetyl-CoA synthetase
8	ELOVL6	ELOVL fatty acid elongase 6
9	G6PD	glucose-6-phosphate dehydrogenase
10	ASS1	argininosuccinate synthase 1
11	TYR	tyrosinase (oculocutaneous albinism IA)
12	PCK2	phosphoenolpyruvate carboxykinase 2 (mitochondrial)
13	ACSS1	acyl-CoA synthetase short-chain family member 1
14	LPIN1	lipin 1
15	ACACA	acetyl-CoA carboxylase alpha

Database:biological process Name:alcohol biosynthetic process ID:GO:0046165
C=133; O=6; E=0.65; R=9.30; rawP=4.45e-05; adjP=0.0005

Index	UserID	Gene Name
1	LSS	lanosterol synthase (2,3-oxidosqualene-lanosterol cyclase)
2	HMGCS1	3-hydroxy-3-methylglutaryl-CoA synthase 1 (soluble)
3	G6PD	glucose-6-phosphate dehydrogenase
4	MVD	mevalonate (diphospho) decarboxylase
5	FDPS	farnesyl diphosphate synthase
6	LPIN1	lipin 1

Database:biological process Name:phosphorus metabolic process ID:GO:0006793
C=2501; O=26; E=12.14; R=2.14; rawP=6.22e-05; adjP=0.0006

Index	UserID	Gene Name
1	SUCLA2	succinate-CoA ligase, ADP-forming, beta subunit
2	ATF4	activating transcription factor 4 (tax-responsive enhancer element B67)
3	ACSL1	acyl-CoA synthetase long-chain family member 1
4	ELOVL6	ELOVL fatty acid elongase 6
5	MVD	mevalonate (diphospho) decarboxylase
6	FGD2	FYVE, RhoGEF and PH domain containing 2
7	CAMK4	calcium/calmodulin-dependent protein kinase IV
8	PDE8A	phosphodiesterase 8A
9	ULK1	unc-51-like kinase 1 (C. elegans)
10	SMAP2	small ArfGAP2
11	LPIN1	lipin 1
12	ASAP2	ArfGAP with SH3 domain, ankyrin repeat and PH domain 2
13	AGPAT2	1-acylglycerol-3-phosphate O-acyltransferase 2
14	SLC9A3R1	solute carrier family 9, subfamily A , member 3 regulator 1
15	ACSS2	acyl-CoA synthetase short-chain family member 2
16	G6PD	glucose-6-phosphate dehydrogenase
17	FDPS	farnesyl diphosphate synthase
18	ALDOA	aldolase A, fructose-bisphosphate
19	ABCA2	ATP-binding cassette, sub-family A (ABC1), member 2
20	DAB1	disabled homolog 1 (Drosophila)
21	PCYT2	phosphate cytidylyltransferase 2, ethanolamine
22	PNPLA3	patatin-like phospholipase domain containing 3

23	ACSS1	acyl-CoA synthetase short-chain family member 1
24	ATP6V0D1	ATPase, H+ transporting, lysosomal 38kDa, V0 subunit d1
25	PPM1K	protein phosphatase, Mg2+/Mn2+ dependent, 1K
26	ACACA	acetyl-CoA carboxylase alpha

Database:biological process Name:organic acid metabolic process ID:GO:0006082
C=974; O=15; E=4.73; R=3.17; rawP=5.41e-05; adjP=0.0006

Index	UserID	Gene Name
1	SUCLA2	succinate-CoA ligase, ADP-forming, beta subunit
2	FADS2	fatty acid desaturase 2
3	SDSL	serine dehydratase-like
4	ATF4	activating transcription factor 4 (tax-responsive enhancer element B67)
5	ACSL1	acyl-CoA synthetase long-chain family member 1
6	ACSS2	acyl-CoA synthetase short-chain family member 2
7	AACS	acetoacetyl-CoA synthetase
8	ELOVL6	ELOVL fatty acid elongase 6
9	G6PD	glucose-6-phosphate dehydrogenase
10	ASS1	argininosuccinate synthase 1
11	TYR	tyrosinase (oculocutaneous albinism IA)
12	PCK2	phosphoenolpyruvate carboxykinase 2 (mitochondrial)
13	ACSS1	acyl-CoA synthetase short-chain family member 1
14	LPIN1	lipin 1
15	ACACA	acetyl-CoA carboxylase alpha

Database:biological process Name:response to drug ID:GO:0042493
C=371; O=9; E=1.80; R=5.00; rawP=7.32e-05; adjP=0.0007

Index	UserID	Gene Name
1	HMGCS1	3-hydroxy-3-methylglutaryl-CoA synthase 1 (soluble)
2	ACSL1	acyl-CoA synthetase long-chain family member 1
3	AACS	acetoacetyl-CoA synthetase
4	MVD	mevalonate (diphospho) decarboxylase
5	ABCA2	ATP-binding cassette, sub-family A (ABC1), member 2
6	ASS1	argininosuccinate synthase 1
7	MVP	major vault protein
8	ACACA	acetyl-CoA carboxylase alpha
9	BAG1	BCL2-associated athanogene

Database:biological process Name:cellular lipid metabolic process ID:GO:0044255
C=813; O=13; E=3.95; R=3.29; rawP=0.0001; adjP=0.0009

Index	UserID	Gene Name
1	FADS2	fatty acid desaturase 2
2	HMGCS1	3-hydroxy-3-methylglutaryl-CoA synthase 1 (soluble)
3	ACSL1	acyl-CoA synthetase long-chain family member 1
4	AGPAT2	1-acylglycerol-3-phosphate O-acyltransferase 2
5	AACS	acetoacetyl-CoA synthetase
6	ELOVL6	ELOVL fatty acid elongase 6
7	MVD	mevalonate (diphospho) decarboxylase

8	FDPS	farnesyl diphosphate synthase
9	PCYT2	phosphate cytidylyltransferase 2, ethanolamine
10	PNPLA3	patatin-like phospholipase domain containing 3
11	ACSS1	acyl-CoA synthetase short-chain family member 1
12	LPIN1	lipin 1
13	ACACA	acetyl-CoA carboxylase alpha

Database:biological process Name:glycerolipid biosynthetic process ID:GO:0045017
C=231; O=7; E=1.12; R=6.24; rawP=0.0001; adjP=0.0009

Index	UserID	Gene Name
1	PCYT2	phosphate cytidylyltransferase 2, ethanolamine
2	ACSL1	acyl-CoA synthetase long-chain family member 1
3	PNPLA3	patatin-like phospholipase domain containing 3
4	AGPAT2	1-acylglycerol-3-phosphate O-acyltransferase 2
5	ELOVL6	ELOVL fatty acid elongase 6
6	ACACA	acetyl-CoA carboxylase alpha
7	LPIN1	lipin 1

Database:biological process Name:coenzyme biosynthetic process ID:GO:0009108
C=105; O=5; E=0.51; R=9.81; rawP=0.0002; adjP=0.0018

Index	UserID	Gene Name
1	ACSL1	acyl-CoA synthetase long-chain family member 1
2	ACSS1	acyl-CoA synthetase short-chain family member 1
3	ACSS2	acyl-CoA synthetase short-chain family member 2
4	ELOVL6	ELOVL fatty acid elongase 6
5	ACACA	acetyl-CoA carboxylase alpha

Database:biological process Name:fatty acid metabolic process ID:GO:0006631
C=282; O=7; E=1.37; R=5.11; rawP=0.0004; adjP=0.0034

Index	UserID	Gene Name
1	FADS2	fatty acid desaturase 2
2	ACSL1	acyl-CoA synthetase long-chain family member 1
3	ACSS1	acyl-CoA synthetase short-chain family member 1
4	AACS	acetoacetyl-CoA synthetase
5	ELOVL6	ELOVL fatty acid elongase 6
6	ACACA	acetyl-CoA carboxylase alpha
7	LPIN1	lipin 1

Database:biological process Name:steroid metabolic process ID:GO:0008202
C=281; O=7; E=1.36; R=5.13; rawP=0.0004; adjP=0.0034

Index	UserID	Gene Name
1	LSS	lanosterol synthase (2,3-oxidosqualene-lanosterol cyclase)
2	HMGCS1	3-hydroxy-3-methylglutaryl-CoA synthase 1 (soluble)
3	YWHAH	tyrosine 3-monooxygenase
4	G6PD	glucose-6-phosphate dehydrogenase
5	MVD	mevalonate (diphospho) decarboxylase

6	FDPS	farnesyl diphosphate synthase
7	OSBPL1A	oxysterol binding protein-like 1A

Database:biological process Name:steroid biosynthetic process ID:GO:0006694
C=143; O=5; E=0.69; R=7.20; rawP=0.0006; adjP=0.0047

Index	UserID	Gene Name
1	LSS	lanosterol synthase (2,3-oxidosqualene-lanosterol cyclase)
2	HMGCS1	3-hydroxy-3-methylglutaryl-CoA synthase 1 (soluble)
3	G6PD	glucose-6-phosphate dehydrogenase
4	MVD	mevalonate (diphospho) decarboxylase
5	FDPS	farnesyl diphosphate synthase

Database:biological process Name:phospholipid biosynthetic process ID:GO:0008654
C=216; O=6; E=1.05; R=5.72; rawP=0.0006; adjP=0.0047

Index	UserID	Gene Name
1	PCYT2	phosphate cytidylyltransferase 2, ethanolamine
2	PNPLA3	patatin-like phospholipase domain containing 3
3	AGPAT2	1-acylglycerol-3-phosphate O-acyltransferase 2
4	MVD	mevalonate (diphospho) decarboxylase
5	FDPS	farnesyl diphosphate synthase
6	LPIN1	lipin 1

Database:biological process Name:regulation of cell morphogenesis ID:GO:0022604
C=300; O=7; E=1.46; R=4.81; rawP=0.0006; adjP=0.0047

Index	UserID	Gene Name
1	DAB1	disabled homolog 1 (Drosophila)
2	ULK1	unc-51-like kinase 1 (C. elegans)
3	YWHAH	tyrosine 3-monooxygenase
4	CIT	citron (rho-interacting, serine/threonine kinase 21)
5	ALDOA	aldolase A, fructose-bisphosphate
6	CDC42EP4	CDC42 effector protein (Rho GTPase binding) 4
7	FGD2	FYVE, RhoGEF and PH domain containing 2

Database:biological process Name:monocarboxylic acid metabolic process ID:GO:0032787
C=406; O=8; E=1.97; R=4.06; rawP=0.0008; adjP=0.0060

Index	UserID	Gene Name
1	FADS2	fatty acid desaturase 2
2	ACSL1	acyl-CoA synthetase long-chain family member 1
3	ACSS2	acyl-CoA synthetase short-chain family member 2
4	AACS	acetoacetyl-CoA synthetase
5	ELOVL6	ELOVL fatty acid elongase 6
6	ACSS1	acyl-CoA synthetase short-chain family member 1
7	LPIN1	lipin 1
8	ACACA	acetyl-CoA carboxylase alpha

Database:biological process Name:glycerolipid metabolic process ID:GO:0046486

C=315; O=7; E=1.53; R=4.58; rawP=0.0008; adjP=0.0060

Index	UserID	Gene Name
1	PCYT2	phosphate cytidylyltransferase 2, ethanolamine
2	ACSL1	acyl-CoA synthetase long-chain family member 1
3	PNPLA3	patatin-like phospholipase domain containing 3
4	AGPAT2	1-acylglycerol-3-phosphate O-acyltransferase 2
5	ELOVL6	ELOVL fatty acid elongase 6
6	ACACA	acetyl-CoA carboxylase alpha
7	LPIN1	lipin 1

Database:molecular function Name:ligase activity, forming carbon-sulfur bonds
C=29; O=6; E=0.13; R=45.17; rawP=3.24e-09; adjP=1.81e-07 ID:GO:0016877

Index	UserID	Gene Name
1	SUCLA2	succinate-CoA ligase, ADP-forming, beta subunit
2	ACSS3	acyl-CoA synthetase short-chain family member 3
3	ACSL1	acyl-CoA synthetase long-chain family member 1
4	ACSS1	acyl-CoA synthetase short-chain family member 1
5	ACSS2	acyl-CoA synthetase short-chain family member 2
6	AACS	acetoacetyl-CoA synthetase

Database:molecular function Name:acid-thiol ligase activity ID:GO:0016878
C=20; O=5; E=0.09; R=54.59; rawP=2.56e-08; adjP=7.17e-07

Index	UserID	Gene Name
1	SUCLA2	succinate-CoA ligase, ADP-forming, beta subunit
2	ACSS3	acyl-CoA synthetase short-chain family member 3
3	ACSS1	acyl-CoA synthetase short-chain family member 1
4	ACSS2	acyl-CoA synthetase short-chain family member 2
5	AACS	acetoacetyl-CoA synthetase

Database:molecular function Name:catalytic activity ID:GO:0003824
C=5371; O=41; E=24.60; R=1.67; rawP=5.15e-05; adjP=0.0010

Index	UserID	Gene Name
1	SDSL	serine dehydratase-like
2	ACSL1	acyl-CoA synthetase long-chain family member 1
3	CIT	citron (rho-interacting, serine/threonine kinase 21)
4	CAMK4	calcium/calmodulin-dependent protein kinase IV
5	ASS1	argininosuccinate synthase 1
6	TYR	tyrosinase (oculocutaneous albinism IA)
7	ULK1	unc-51-like kinase 1 (C. elegans)
8	PAK4	p21 protein (Cdc42/Rac)-activated kinase 4
9	LPIN1	lipin 1
10	ECHDC3	enoyl CoA hydratase domain containing 3
11	HMGCS1	3-hydroxy-3-methylglutaryl-CoA synthase 1 (soluble)
12	ACSS2	acyl-CoA synthetase short-chain family member 2
13	RNF41	ring finger protein 41
14	PRUNE2	prune homolog 2 (Drosophila)
15	ABCA2	ATP-binding cassette, sub-family A (ABC1), member 2

16	PCYT2	phosphate cytidylyltransferase 2, ethanolamine
17	PCK2	phosphoenolpyruvate carboxykinase 2 (mitochondrial)
18	PNPLA3	patatin-like phospholipase domain containing 3
19	ATP6V0D1	ATPase, H+ transporting, lysosomal 38kDa, V0 subunit d1
20	DDX1	DEAD (Asp-Glu-Ala-Asp) box helicase 1
21	PPM1K	protein phosphatase, Mg2+/Mn2+ dependent, 1K
22	ACACA	acetyl-CoA carboxylase alpha
23	SUCLA2	succinate-CoA ligase, ADP-forming, beta subunit
24	FADS2	fatty acid desaturase 2
25	AACS	acetoacetyl-CoA synthetase
26	ELOVL6	ELOVL fatty acid elongase 6
27	MVD	mevalonate (diphospho) decarboxylase
28	CRBN	cereblon
29	PDE8A	phosphodiesterase 8A
30	KDM1B	lysine (K)-specific demethylase 1B
31	LSS	lanosterol synthase (2,3-oxidosqualene-lanosterol cyclase)
32	ACSS3	acyl-CoA synthetase short-chain family member 3
33	AZGP1	alpha-2-glycoprotein 1, zinc-binding
34	PAFAH1B3	platelet-activating factor acetylhydrolase 1b, catalytic subunit 3
35	AGPAT2	1-acylglycerol-3-phosphate O-acyltransferase 2
36	G6PD	glucose-6-phosphate dehydrogenase
37	FDPS	farnesyl diphosphate synthase
38	ALDOA	aldolase A, fructose-bisphosphate
39	MYO18A	myosin XVIIIA
40	HSPB8	heat shock 22kDa protein 8
41	ACSS1	acyl-CoA synthetase short-chain family member 1

Database:molecular function Name:ligase activity ID:GO:0016874
C=483; O=9; E=2.21; R=4.07; rawP=0.0003; adjP=0.0042

Index	UserID	Gene Name
1	SUCLA2	succinate-CoA ligase, ADP-forming, beta subunit
2	ACSS3	acyl-CoA synthetase short-chain family member 3
3	ACSL1	acyl-CoA synthetase long-chain family member 1
4	ACSS2	acyl-CoA synthetase short-chain family member 2
5	AACS	acetoacetyl-CoA synthetase
6	RNF41	ring finger protein 41
7	ASS1	argininosuccinate synthase 1
8	ACSS1	acyl-CoA synthetase short-chain family member 1
9	ACACA	acetyl-CoA carboxylase alpha

Database:molecular function Name:purine nucleotide binding ID:GO:0017076
C=1871; O=18; E=8.57; R=2.10; rawP=0.0015; adjP=0.0056

Index	UserID	Gene Name
1	SUCLA2	succinate-CoA ligase, ADP-forming, beta subunit
2	ACSL1	acyl-CoA synthetase long-chain family member 1
3	AACS	acetoacetyl-CoA synthetase
4	CIT	citron (rho-interacting, serine/threonine kinase 21)
5	MVD	mevalonate (diphospho) decarboxylase

6	RASEF	RAS and EF-hand domain containing
7	CAMK4	calcium/calmodulin-dependent protein kinase IV
8	ASS1	argininosuccinate synthase 1
9	ULK1	unc-51-like kinase 1 (C. elegans)
10	PAK4	p21 protein (Cdc42/Rac)-activated kinase 4
11	ACSS3	acyl-CoA synthetase short-chain family member 3
12	ACSS2	acyl-CoA synthetase short-chain family member 2
13	MYO18A	myosin XVIIIA
14	ABCA2	ATP-binding cassette, sub-family A (ABC1), member 2
15	PCK2	phosphoenolpyruvate carboxykinase 2 (mitochondrial)
16	ACSS1	acyl-CoA synthetase short-chain family member 1
17	DDX1	DEAD (Asp-Glu-Ala-Asp) box helicase 1
18	ACACA	acetyl-CoA carboxylase alpha

Database:molecular function Name:purine ribonucleoside binding ID:GO:0032550
C=1838; O=18; E=8.42; R=2.14; rawP=0.0012; adjP=0.0056

Index	UserID	Gene Name
1	SUCLA2	succinate-CoA ligase, ADP-forming, beta subunit
2	ACSL1	acyl-CoA synthetase long-chain family member 1
3	AACS	acetoacetyl-CoA synthetase
4	CIT	citron (rho-interacting, serine/threonine kinase 21)
5	MVD	mevalonate (diphospho) decarboxylase
6	RASEF	RAS and EF-hand domain containing
7	CAMK4	calcium/calmodulin-dependent protein kinase IV
8	ASS1	argininosuccinate synthase 1
9	ULK1	unc-51-like kinase 1 (C. elegans)
10	PAK4	p21 protein (Cdc42/Rac)-activated kinase 4
11	ACSS3	acyl-CoA synthetase short-chain family member 3
12	ACSS2	acyl-CoA synthetase short-chain family member 2
13	MYO18A	myosin XVIIIA
14	ABCA2	ATP-binding cassette, sub-family A (ABC1), member 2
15	PCK2	phosphoenolpyruvate carboxykinase 2 (mitochondrial)
16	ACSS1	acyl-CoA synthetase short-chain family member 1
17	DDX1	DEAD (Asp-Glu-Ala-Asp) box helicase 1
18	ACACA	acetyl-CoA carboxylase alpha

Database:molecular function Name:ribonucleoside binding ID:GO:0032549
C=1842; O=18; E=8.44; R=2.13; rawP=0.0013; adjP=0.0056

Index	UserID	Gene Name
1	SUCLA2	succinate-CoA ligase, ADP-forming, beta subunit
2	ACSL1	acyl-CoA synthetase long-chain family member 1
3	AACS	acetoacetyl-CoA synthetase
4	CIT	citron (rho-interacting, serine/threonine kinase 21)
5	MVD	mevalonate (diphospho) decarboxylase
6	RASEF	RAS and EF-hand domain containing
7	CAMK4	calcium/calmodulin-dependent protein kinase IV
8	ASS1	argininosuccinate synthase 1
9	ULK1	unc-51-like kinase 1 (C. elegans)

10	PAK4	p21 protein (Cdc42/Rac)-activated kinase 4
11	ACSS3	acyl-CoA synthetase short-chain family member 3
12	ACSS2	acyl-CoA synthetase short-chain family member 2
13	MYO18A	myosin XVIIIA
14	ABCA2	ATP-binding cassette, sub-family A (ABC1), member 2
15	PCK2	phosphoenolpyruvate carboxykinase 2 (mitochondrial)
16	ACSS1	acyl-CoA synthetase short-chain family member 1
17	DDX1	DEAD (Asp-Glu-Ala-Asp) box helicase 1
18	ACACA	acetyl-CoA carboxylase alpha

Database:molecular function Name:purine ribonucleotide binding ID:GO:0032555
C=1864; O=18; E=8.54; R=2.11; rawP=0.0015; adjP=0.0056

Index	UserID	Gene Name
1	SUCLA2	succinate-CoA ligase, ADP-forming, beta subunit
2	ACSL1	acyl-CoA synthetase long-chain family member 1
3	AACS	acetoacetyl-CoA synthetase
4	CIT	citron (rho-interacting, serine/threonine kinase 21)
5	MVD	mevalonate (diphospho) decarboxylase
6	RASEF	RAS and EF-hand domain containing
7	CAMK4	calcium/calmodulin-dependent protein kinase IV
8	ASS1	argininosuccinate synthase 1
9	ULK1	unc-51-like kinase 1 (C. elegans)
10	PAK4	p21 protein (Cdc42/Rac)-activated kinase 4
11	ACSS3	acyl-CoA synthetase short-chain family member 3
12	ACSS2	acyl-CoA synthetase short-chain family member 2
13	MYO18A	myosin XVIIIA
14	ABCA2	ATP-binding cassette, sub-family A (ABC1), member 2
15	PCK2	phosphoenolpyruvate carboxykinase 2 (mitochondrial)
16	ACSS1	acyl-CoA synthetase short-chain family member 1
17	DDX1	DEAD (Asp-Glu-Ala-Asp) box helicase 1
18	ACACA	acetyl-CoA carboxylase alpha

Database:molecular function Name:purine ribonucleoside triphosphate binding
C=1829; O=18; E=8.38; R=2.15; rawP=0.0012; adjP=0.0056 ID:GO:0035639

Index	UserID	Gene Name
1	SUCLA2	succinate-CoA ligase, ADP-forming, beta subunit
2	ACSL1	acyl-CoA synthetase long-chain family member 1
3	AACS	acetoacetyl-CoA synthetase
4	CIT	citron (rho-interacting, serine/threonine kinase 21)
5	MVD	mevalonate (diphospho) decarboxylase
6	RASEF	RAS and EF-hand domain containing
7	CAMK4	calcium/calmodulin-dependent protein kinase IV
8	ASS1	argininosuccinate synthase 1
9	ULK1	unc-51-like kinase 1 (C. elegans)
10	PAK4	p21 protein (Cdc42/Rac)-activated kinase 4
11	ACSS3	acyl-CoA synthetase short-chain family member 3
12	ACSS2	acyl-CoA synthetase short-chain family member 2
13	MYO18A	myosin XVIIIA

14	ABCA2	ATP-binding cassette, sub-family A (ABC1), member 2
15	PCK2	phosphoenolpyruvate carboxykinase 2 (mitochondrial)
16	ACSS1	acyl-CoA synthetase short-chain family member 1
17	DDX1	DEAD (Asp-Glu-Ala-Asp) box helicase 1
18	ACACA	acetyl-CoA carboxylase alpha

Database:molecular function Name:anion binding ID:GO:0043168
C=2402; O=22; E=11.00; R=2.00; rawP=0.0008; adjP=0.0056

Index	UserID	Gene Name
1	SUCLA2	succinate-CoA ligase, ADP-forming, beta subunit
2	SDSL	serine dehydratase-like
3	ACSL1	acyl-CoA synthetase long-chain family member 1
4	AACS	acetoacetyl-CoA synthetase
5	CIT	citron (rho-interacting, serine/threonine kinase 21)
6	MVD	mevalonate (diphospho) decarboxylase
7	RASEF	RAS and EF-hand domain containing
8	CAMK4	calcium/calmodulin-dependent protein kinase IV
9	ASS1	argininosuccinate synthase 1
10	ULK1	unc-51-like kinase 1 (C. elegans)
11	KDM1B	lysine (K)-specific demethylase 1B
12	PAK4	p21 protein (Cdc42/Rac)-activated kinase 4
13	ACSS3	acyl-CoA synthetase short-chain family member 3
14	AZGP1	alpha-2-glycoprotein 1, zinc-binding
15	ACSS2	acyl-CoA synthetase short-chain family member 2
16	MYO18A	myosin XVIIIA
17	ABCA2	ATP-binding cassette, sub-family A (ABC1), member 2
18	FST	follistatin
19	PCK2	phosphoenolpyruvate carboxykinase 2 (mitochondrial)
20	ACSS1	acyl-CoA synthetase short-chain family member 1
21	DDX1	DEAD (Asp-Glu-Ala-Asp) box helicase 1
22	ACACA	acetyl-CoA carboxylase alpha

Database:molecular function Name:adenyl nucleotide binding ID:GO:0030554
C=1516; O=16; E=6.94; R=2.30; rawP=0.0011; adjP=0.0056

Index	UserID	Gene Name
1	SUCLA2	succinate-CoA ligase, ADP-forming, beta subunit
2	ACSL1	acyl-CoA synthetase long-chain family member 1
3	AACS	acetoacetyl-CoA synthetase
4	CIT	citron (rho-interacting, serine/threonine kinase 21)
5	MVD	mevalonate (diphospho) decarboxylase
6	CAMK4	calcium/calmodulin-dependent protein kinase IV
7	ASS1	argininosuccinate synthase 1
8	ULK1	unc-51-like kinase 1 (C. elegans)
9	PAK4	p21 protein (Cdc42/Rac)-activated kinase 4
10	ACSS3	acyl-CoA synthetase short-chain family member 3
11	ACSS2	acyl-CoA synthetase short-chain family member 2
12	MYO18A	myosin XVIIIA
13	ABCA2	ATP-binding cassette, sub-family A (ABC1), member 2

14	ACSS1	acyl-CoA synthetase short-chain family member 1
15	DDX1	DEAD (Asp-Glu-Ala-Asp) box helicase 1
16	ACACA	acetyl-CoA carboxylase alpha

Database:molecular function Name:nucleoside binding ID:GO:0001882
C=1852; O=18; E=8.48; R=2.12; rawP=0.0014; adjP=0.0056

Index	UserID	Gene Name
1	SUCLA2	succinate-CoA ligase, ADP-forming, beta subunit
2	ACSL1	acyl-CoA synthetase long-chain family member 1
3	AACS	acetoacetyl-CoA synthetase
4	CIT	citron (rho-interacting, serine/threonine kinase 21)
5	MVD	mevalonate (diphospho) decarboxylase
6	RASEF	RAS and EF-hand domain containing
7	CAMK4	calcium/calmodulin-dependent protein kinase IV
8	ASS1	argininosuccinate synthase 1
9	ULK1	unc-51-like kinase 1 (C. elegans)
10	PAK4	p21 protein (Cdc42/Rac)-activated kinase 4
11	ACSS3	acyl-CoA synthetase short-chain family member 3
12	ACSS2	acyl-CoA synthetase short-chain family member 2
13	MYO18A	myosin XVIIIA
14	ABCA2	ATP-binding cassette, sub-family A (ABC1), member 2
15	PCK2	phosphoenolpyruvate carboxykinase 2 (mitochondrial)
16	ACSS1	acyl-CoA synthetase short-chain family member 1
17	DDX1	DEAD (Asp-Glu-Ala-Asp) box helicase 1
18	ACACA	acetyl-CoA carboxylase alpha

Database:molecular function Name:purine nucleoside binding ID:GO:0001883
C=1841; O=18; E=8.43; R=2.13; rawP=0.0013; adjP=0.0056

Index	UserID	Gene Name
1	SUCLA2	succinate-CoA ligase, ADP-forming, beta subunit
2	ACSL1	acyl-CoA synthetase long-chain family member 1
3	AACS	acetoacetyl-CoA synthetase
4	CIT	citron (rho-interacting, serine/threonine kinase 21)
5	MVD	mevalonate (diphospho) decarboxylase
6	RASEF	RAS and EF-hand domain containing
7	CAMK4	calcium/calmodulin-dependent protein kinase IV
8	ASS1	argininosuccinate synthase 1
9	ULK1	unc-51-like kinase 1 (C. elegans)
10	PAK4	p21 protein (Cdc42/Rac)-activated kinase 4
11	ACSS3	acyl-CoA synthetase short-chain family member 3
12	ACSS2	acyl-CoA synthetase short-chain family member 2
13	MYO18A	myosin XVIIIA
14	ABCA2	ATP-binding cassette, sub-family A (ABC1), member 2
15	PCK2	phosphoenolpyruvate carboxykinase 2 (mitochondrial)
16	ACSS1	acyl-CoA synthetase short-chain family member 1
17	DDX1	DEAD (Asp-Glu-Ala-Asp) box helicase 1
18	ACACA	acetyl-CoA carboxylase alpha

Database:molecular function Name:ATP binding ID:GO:0005524
C=1482; O=16; E=6.79; R=2.36; rawP=0.0009; adjP=0.0056

Index	UserID	Gene Name
1	SUCLA2	succinate-CoA ligase, ADP-forming, beta subunit
2	ACSL1	acyl-CoA synthetase long-chain family member 1
3	AACS	acetoacetyl-CoA synthetase
4	CIT	citron (rho-interacting, serine/threonine kinase 21)
5	MVD	mevalonate (diphospho) decarboxylase
6	CAMK4	calcium/calmodulin-dependent protein kinase IV
7	ASS1	argininosuccinate synthase 1
8	ULK1	unc-51-like kinase 1 (C. elegans)
9	PAK4	p21 protein (Cdc42/Rac)-activated kinase 4
10	ACSS3	acyl-CoA synthetase short-chain family member 3
11	ACSS2	acyl-CoA synthetase short-chain family member 2
12	MYO18A	myosin XVIIIA
13	ABCA2	ATP-binding cassette, sub-family A (ABC1), member 2
14	ACSS1	acyl-CoA synthetase short-chain family member 1
15	DDX1	DEAD (Asp-Glu-Ala-Asp) box helicase 1
16	ACACA	acetyl-CoA carboxylase alpha

Database:molecular function Name:ribonucleotide binding ID:GO:0032553
C=1879; O=18; E=8.61; R=2.09; rawP=0.0016; adjP=0.0056

Index	UserID	Gene Name
1	SUCLA2	succinate-CoA ligase, ADP-forming, beta subunit
2	ACSL1	acyl-CoA synthetase long-chain family member 1
3	AACS	acetoacetyl-CoA synthetase
4	CIT	citron (rho-interacting, serine/threonine kinase 21)
5	MVD	mevalonate (diphospho) decarboxylase
6	RASEF	RAS and EF-hand domain containing
7	CAMK4	calcium/calmodulin-dependent protein kinase IV
8	ASS1	argininosuccinate synthase 1
9	ULK1	unc-51-like kinase 1 (C. elegans)
10	PAK4	p21 protein (Cdc42/Rac)-activated kinase 4
11	ACSS3	acyl-CoA synthetase short-chain family member 3
12	ACSS2	acyl-CoA synthetase short-chain family member 2
13	MYO18A	myosin XVIIIA
14	ABCA2	ATP-binding cassette, sub-family A (ABC1), member 2
15	PCK2	phosphoenolpyruvate carboxykinase 2 (mitochondrial)
16	ACSS1	acyl-CoA synthetase short-chain family member 1
17	DDX1	DEAD (Asp-Glu-Ala-Asp) box helicase 1
18	ACACA	acetyl-CoA carboxylase alpha

Database:molecular function Name:adenyl ribonucleotide binding ID:GO:0032559
C=1512; O=16; E=6.92; R=2.31; rawP=0.0011; adjP=0.0056

Index	UserID	Gene Name
1	SUCLA2	succinate-CoA ligase, ADP-forming, beta subunit
2	ACSL1	acyl-CoA synthetase long-chain family member 1
3	AACS	acetoacetyl-CoA synthetase

4	CIT	citron (rho-interacting, serine/threonine kinase 21)
5	MVD	mevalonate (diphospho) decarboxylase
6	CAMK4	calcium/calmodulin-dependent protein kinase IV
7	ASS1	argininosuccinate synthase 1
8	ULK1	unc-51-like kinase 1 (C. elegans)
9	PAK4	p21 protein (Cdc42/Rac)-activated kinase 4
10	ACSS3	acyl-CoA synthetase short-chain family member 3
11	ACSS2	acyl-CoA synthetase short-chain family member 2
12	MYO18A	myosin XVIIIA
13	ABCA2	ATP-binding cassette, sub-family A (ABC1), member 2
14	ACSS1	acyl-CoA synthetase short-chain family member 1
15	DDX1	DEAD (Asp-Glu-Ala-Asp) box helicase 1
16	ACACA	acetyl-CoA carboxylase alpha

Database:molecular function Name:small molecule binding ID:GO:0036094
C=2630; O=22; E=12.05; R=1.83; rawP=0.0026; adjP=0.0086

Index	UserID	Gene Name
1	SUCLA2	succinate-CoA ligase, ADP-forming, beta subunit
2	SDSL	serine dehydratase-like
3	ACSL1	acyl-CoA synthetase long-chain family member 1
4	AACS	acetoacetyl-CoA synthetase
5	CIT	citron (rho-interacting, serine/threonine kinase 21)
6	MVD	mevalonate (diphospho) decarboxylase
7	RASEF	RAS and EF-hand domain containing
8	CAMK4	calcium/calmodulin-dependent protein kinase IV
9	ASS1	argininosuccinate synthase 1
10	ULK1	unc-51-like kinase 1 (C. elegans)
11	KDM1B	lysine (K)-specific demethylase 1B
12	PAK4	p21 protein (Cdc42/Rac)-activated kinase 4
13	ACSS3	acyl-CoA synthetase short-chain family member 3
14	HMGCS1	3-hydroxy-3-methylglutaryl-CoA synthase 1 (soluble)
15	ACSS2	acyl-CoA synthetase short-chain family member 2
16	G6PD	glucose-6-phosphate dehydrogenase
17	MYO18A	myosin XVIIIA
18	ABCA2	ATP-binding cassette, sub-family A (ABC1), member 2
19	PCK2	phosphoenolpyruvate carboxykinase 2 (mitochondrial)
20	ACSS1	acyl-CoA synthetase short-chain family member 1
21	DDX1	DEAD (Asp-Glu-Ala-Asp) box helicase 1
22	ACACA	acetyl-CoA carboxylase alpha

Database:molecular function Name:transferase activity, transferring acyl groups
C=233; O=5; E=1.07; R=4.69; rawP=0.0043; adjP=0.0134 ID:GO:0016746

Index	UserID	Gene Name
1	HMGCS1	3-hydroxy-3-methylglutaryl-CoA synthase 1 (soluble)
2	PNPLA3	patatin-like phospholipase domain containing 3
3	AGPAT2	1-acylglycerol-3-phosphate O-acyltransferase 2
4	PAFAH1B3	platelet-activating factor acetylhydrolase 1b, catalytic subunit 3
5	ELOVL6	ELOVL fatty acid elongase 6

Database:molecular function Name:nucleoside phosphate binding ID:GO:1901265
C=2437; O=20; E=11.16; R=1.79; rawP=0.0053; adjP=0.0148

Index	UserID	Gene Name
1	SUCLA2	succinate-CoA ligase, ADP-forming, beta subunit
2	ACSL1	acyl-CoA synthetase long-chain family member 1
3	AACS	acetoacetyl-CoA synthetase
4	CIT	citron (rho-interacting, serine/threonine kinase 21)
5	MVD	mevalonate (diphospho) decarboxylase
6	RASEF	RAS and EF-hand domain containing
7	CAMK4	calcium/calmodulin-dependent protein kinase IV
8	ASS1	argininosuccinate synthase 1
9	ULK1	unc-51-like kinase 1 (C. elegans)
10	KDM1B	lysine (K)-specific demethylase 1B
11	PAK4	p21 protein (Cdc42/Rac)-activated kinase 4
12	ACSS3	acyl-CoA synthetase short-chain family member 3
13	ACSS2	acyl-CoA synthetase short-chain family member 2
14	G6PD	glucose-6-phosphate dehydrogenase
15	MYO18A	myosin XVIIIA
16	ABCA2	ATP-binding cassette, sub-family A (ABC1), member 2
17	PCK2	phosphoenolpyruvate carboxykinase 2 (mitochondrial)
18	ACSS1	acyl-CoA synthetase short-chain family member 1
19	DDX1	DEAD (Asp-Glu-Ala-Asp) box helicase 1
20	ACACA	acetyl-CoA carboxylase alpha

Database:molecular function Name:nucleotide binding ID:GO:0000166
C=2436; O=20; E=11.16; R=1.79; rawP=0.0053; adjP=0.0148

Index	UserID	Gene Name
1	SUCLA2	succinate-CoA ligase, ADP-forming, beta subunit
2	ACSL1	acyl-CoA synthetase long-chain family member 1
3	AACS	acetoacetyl-CoA synthetase
4	CIT	citron (rho-interacting, serine/threonine kinase 21)
5	MVD	mevalonate (diphospho) decarboxylase
6	RASEF	RAS and EF-hand domain containing
7	CAMK4	calcium/calmodulin-dependent protein kinase IV
8	ASS1	argininosuccinate synthase 1
9	ULK1	unc-51-like kinase 1 (C. elegans)
10	KDM1B	lysine (K)-specific demethylase 1B
11	PAK4	p21 protein (Cdc42/Rac)-activated kinase 4
12	ACSS3	acyl-CoA synthetase short-chain family member 3
13	ACSS2	acyl-CoA synthetase short-chain family member 2
14	G6PD	glucose-6-phosphate dehydrogenase
15	MYO18A	myosin XVIIIA
16	ABCA2	ATP-binding cassette, sub-family A (ABC1), member 2
17	PCK2	phosphoenolpyruvate carboxykinase 2 (mitochondrial)
18	ACSS1	acyl-CoA synthetase short-chain family member 1
19	DDX1	DEAD (Asp-Glu-Ala-Asp) box helicase 1
20	ACACA	acetyl-CoA carboxylase alpha

Database:molecular function Name:ATPase activity, coupled ID:GO:0042623
C=277; O=5; E=1.27; R=3.94; rawP=0.0088; adjP=0.0235

Index	UserID	Gene Name
1	ATP6V0D1	ATPase, H+ transporting, lysosomal 38kDa, V0 subunit d1
2	DDX1	DEAD (Asp-Glu-Ala-Asp) box helicase 1
3	MYO18A	myosin XVIIIA
4	CRBN	cereblon
5	ABCA2	ATP-binding cassette, sub-family A (ABC1), member 2

Database:molecular function Name:ion binding ID:GO:0043167
C=5820; O=36; E=26.66; R=1.35; rawP=0.0155; adjP=0.0395

Index	UserID	Gene Name
1	SDSL	serine dehydratase-like
2	ACSL1	acyl-CoA synthetase long-chain family member 1
3	CIT	citron (rho-interacting, serine/threonine kinase 21)
4	FGD2	FYVE, RhoGEF and PH domain containing 2
5	CAMK4	calcium/calmodulin-dependent protein kinase IV
6	ASS1	argininosuccinate synthase 1
7	TYR	tyrosinase (oculocutaneous albinism IA)
8	ULK1	unc-51-like kinase 1 (C. elegans)
9	PAK4	p21 protein (Cdc42/Rac)-activated kinase 4
10	NELL2	NEL-like 2 (chicken)
11	ASAP2	ArfGAP with SH3 domain, ankyrin repeat and PH domain 2
12	ACSS2	acyl-CoA synthetase short-chain family member 2
13	RNF41	ring finger protein 41
14	PRUNE2	prune homolog 2 (Drosophila)
15	ABCA2	ATP-binding cassette, sub-family A (ABC1), member 2
16	PCK2	phosphoenolpyruvate carboxykinase 2 (mitochondrial)
17	DDX1	DEAD (Asp-Glu-Ala-Asp) box helicase 1
18	PPM1K	protein phosphatase, Mg2+/Mn2+ dependent, 1K
19	ACACA	acetyl-CoA carboxylase alpha
20	SUCLA2	succinate-CoA ligase, ADP-forming, beta subunit
21	FADS2	fatty acid desaturase 2
22	RASEF	RAS and EF-hand domain containing
23	MVD	mevalonate (diphospho) decarboxylase
24	AACS	acetoacetyl-CoA synthetase
25	EFHD1	EF-hand domain family, member D1
26	MBNL3	muscleblind-like splicing regulator 3
27	PDE8A	phosphodiesterase 8A
28	KDM1B	lysine (K)-specific demethylase 1B
29	SMAP2	small ArfGAP2
30	RPA1	replication protein A1, 70kDa
31	ACSS3	acyl-CoA synthetase short-chain family member 3
32	AZGP1	alpha-2-glycoprotein 1, zinc-binding
33	FDPS	farnesyl diphosphate synthase
34	MYO18A	myosin XVIIIA
35	FST	follistatin

36	ACSS1	acyl-CoA synthetase short-chain family member 1

Database:cellular component Name:cytoplasm ID:GO:0005737
C=9130; O=63; E=39.08; R=1.61; rawP=2.66e-09; adjP=1.54e-07

Index	UserID	Gene Name
1	SDSL	serine dehydratase-like
2	ACSL1	acyl-CoA synthetase long-chain family member 1
3	CIT	citron (rho-interacting, serine/threonine kinase 21)
4	FAM129B	family with sequence similarity 129, member B
5	FGD2	FYVE, RhoGEF and PH domain containing 2
6	CAMK4	calcium/calmodulin-dependent protein kinase IV
7	ASS1	argininosuccinate synthase 1
8	TYR	tyrosinase (oculocutaneous albinism IA)
9	ULK1	unc-51-like kinase 1 (C. elegans)
10	PAK4	p21 protein (Cdc42/Rac)-activated kinase 4
11	CLIC2	chloride intracellular channel 2
12	LPIN1	lipin 1
13	CDC42EP4	CDC42 effector protein (Rho GTPase binding) 4
14	ECHDC3	enoyl CoA hydratase domain containing 3
15	HMGCS1	3-hydroxy-3-methylglutaryl-CoA synthase 1 (soluble)
16	ASAP2	ArfGAP with SH3 domain, ankyrin repeat and PH domain 2
17	SLC9A3R1	solute carrier family 9, subfamily A , member 3 regulator 1
18	ARHGAP18	Rho GTPase activating protein 18
19	ACSS2	acyl-CoA synthetase short-chain family member 2
20	RNF41	ring finger protein 41
21	PRUNE2	prune homolog 2 (Drosophila)
22	ABCA2	ATP-binding cassette, sub-family A (ABC1), member 2
23	PCYT2	phosphate cytidylyltransferase 2, ethanolamine
24	EPB41L4B	erythrocyte membrane protein band 4.1 like 4B
25	PCK2	phosphoenolpyruvate carboxykinase 2 (mitochondrial)
26	PNPLA3	patatin-like phospholipase domain containing 3
27	MVP	major vault protein
28	ATP6V0D1	ATPase, H+ transporting, lysosomal 38kDa, V0 subunit d1
29	PPM1K	protein phosphatase, Mg2+/Mn2+ dependent, 1K
30	DDX1	DEAD (Asp-Glu-Ala-Asp) box helicase 1
31	ACACA	acetyl-CoA carboxylase alpha
32	SUCLA2	succinate-CoA ligase, ADP-forming, beta subunit
33	PDE4DIP	phosphodiesterase 4D interacting protein
34	FADS2	fatty acid desaturase 2
35	ATF4	activating transcription factor 4 (tax-responsive enhancer element B67)
36	AACS	acetoacetyl-CoA synthetase
37	ELOVL6	ELOVL fatty acid elongase 6
38	RASEF	RAS and EF-hand domain containing
39	MVD	mevalonate (diphospho) decarboxylase
40	EFHD1	EF-hand domain family, member D1
41	MBNL3	muscleblind-like splicing regulator 3
42	CRBN	cereblon
43	PDE8A	phosphodiesterase 8A
44	SMAP2	small ArfGAP2

45	RPA1	replication protein A1, 70kDa
46	OSBPL1A	oxysterol binding protein-like 1A
47	LSS	lanosterol synthase (2,3-oxidosqualene-lanosterol cyclase)
48	ACSS3	acyl-CoA synthetase short-chain family member 3
49	PAFAH1B3	platelet-activating factor acetylhydrolase 1b, catalytic subunit 3
50	AGPAT2	1-acylglycerol-3-phosphate O-acyltransferase 2
51	G6PD	glucose-6-phosphate dehydrogenase
52	FDPS	farnesyl diphosphate synthase
53	ALDOA	aldolase A, fructose-bisphosphate
54	MYO18A	myosin XVIIIA
55	HSPB8	heat shock 22kDa protein 8
56	DAB1	disabled homolog 1 (Drosophila)
57	FST	follistatin
58	YWHAH	tyrosine 3-monooxygenase
59	ACSS1	acyl-CoA synthetase short-chain family member 1
60	NRSN2	neurensin 2
61	RUSC1	RUN and SH3 domain containing 1
62	BAG1	BCL2-associated athanogene
63	CHMP4C	charged multivesicular body protein 4C

Database:cellular component Name:cytoplasmic part ID:GO:0044444
C=6772; O=53; E=28.99; R=1.83; rawP=1.17e-08; adjP=3.39e-07

Index	UserID	Gene Name
1	SDSL	serine dehydratase-like
2	ACSL1	acyl-CoA synthetase long-chain family member 1
3	CIT	citron (rho-interacting, serine/threonine kinase 21)
4	FAM129B	family with sequence similarity 129, member B
5	FGD2	FYVE, RhoGEF and PH domain containing 2
6	CAMK4	calcium/calmodulin-dependent protein kinase IV
7	ASS1	argininosuccinate synthase 1
8	TYR	tyrosinase (oculocutaneous albinism IA)
9	ULK1	unc-51-like kinase 1 (C. elegans)
10	PAK4	p21 protein (Cdc42/Rac)-activated kinase 4
11	LPIN1	lipin 1
12	ECHDC3	enoyl CoA hydratase domain containing 3
13	HMGCS1	3-hydroxy-3-methylglutaryl-CoA synthase 1 (soluble)
14	ASAP2	ArfGAP with SH3 domain, ankyrin repeat and PH domain 2
15	SLC9A3R1	solute carrier family 9, subfamily A , member 3 regulator 1
16	ARHGAP18	Rho GTPase activating protein 18
17	ACSS2	acyl-CoA synthetase short-chain family member 2
18	RNF41	ring finger protein 41
19	PRUNE2	prune homolog 2 (Drosophila)
20	ABCA2	ATP-binding cassette, sub-family A (ABC1), member 2
21	PCYT2	phosphate cytidylyltransferase 2, ethanolamine
22	PCK2	phosphoenolpyruvate carboxykinase 2 (mitochondrial)
23	PNPLA3	patatin-like phospholipase domain containing 3
24	ATP6V0D1	ATPase, H+ transporting, lysosomal 38kDa, V0 subunit d1
25	DDX1	DEAD (Asp-Glu-Ala-Asp) box helicase 1
26	PPM1K	protein phosphatase, Mg2+/Mn2+ dependent, 1K
27	ACACA	acetyl-CoA carboxylase alpha

28	SUCLA2	succinate-CoA ligase, ADP-forming, beta subunit
29	PDE4DIP	phosphodiesterase 4D interacting protein
30	FADS2	fatty acid desaturase 2
31	ATF4	activating transcription factor 4 (tax-responsive enhancer element B67)
32	AACS	acetoacetyl-CoA synthetase
33	ELOVL6	ELOVL fatty acid elongase 6
34	RASEF	RAS and EF-hand domain containing
35	MVD	mevalonate (diphospho) decarboxylase
36	EFHD1	EF-hand domain family, member D1
37	MBNL3	muscleblind-like splicing regulator 3
38	PDE8A	phosphodiesterase 8A
39	OSBPL1A	oxysterol binding protein-like 1A
40	LSS	lanosterol synthase (2,3-oxidosqualene-lanosterol cyclase)
41	ACSS3	acyl-CoA synthetase short-chain family member 3
42	PAFAH1B3	platelet-activating factor acetylhydrolase 1b, catalytic subunit 3
43	AGPAT2	1-acylglycerol-3-phosphate O-acyltransferase 2
44	G6PD	glucose-6-phosphate dehydrogenase
45	FDPS	farnesyl diphosphate synthase
46	ALDOA	aldolase A, fructose-bisphosphate
47	MYO18A	myosin XVIIIA
48	DAB1	disabled homolog 1 (Drosophila)
49	ACSS1	acyl-CoA synthetase short-chain family member 1
50	NRSN2	neurensin 2
51	RUSC1	RUN and SH3 domain containing 1
52	BAG1	BCL2-associated athanogene
53	CHMP4C	charged multivesicular body protein 4C

Database:cellular component Name:intracellular part ID:GO:0044424
C=12237; O=66; E=52.38; R=1.26; rawP=9.34e-05; adjP=0.0018

Index	UserID	Gene Name
1	SDSL	serine dehydratase-like
2	ACSL1	acyl-CoA synthetase long-chain family member 1
3	FAM129B	family with sequence similarity 129, member B
4	FGD2	FYVE, RhoGEF and PH domain containing 2
5	ASS1	argininosuccinate synthase 1
6	TYR	tyrosinase (oculocutaneous albinism IA)
7	CLIC2	chloride intracellular channel 2
8	LPIN1	lipin 1
9	CDC42EP4	CDC42 effector protein (Rho GTPase binding) 4
10	ASAP2	ArfGAP with SH3 domain, ankyrin repeat and PH domain 2
11	ARHGAP18	Rho GTPase activating protein 18
12	ACSS2	acyl-CoA synthetase short-chain family member 2
13	RNF41	ring finger protein 41
14	FAM107A	family with sequence similarity 107, member A
15	ABCA2	ATP-binding cassette, sub-family A (ABC1), member 2
16	EPB41L4B	erythrocyte membrane protein band 4.1 like 4B
17	PPM1K	protein phosphatase, Mg2+/Mn2+ dependent, 1K
18	DDX1	DEAD (Asp-Glu-Ala-Asp) box helicase 1
19	ACACA	acetyl-CoA carboxylase alpha

20	ATF4	activating transcription factor 4 (tax-responsive enhancer element B67)
21	RASEF	RAS and EF-hand domain containing
22	MVD	mevalonate (diphospho) decarboxylase
23	KDM1B	lysine (K)-specific demethylase 1B
24	OSBPL1A	oxysterol binding protein-like 1A
25	LSS	lanosterol synthase (2,3-oxidosqualene-lanosterol cyclase)
26	PAFAH1B3	platelet-activating factor acetylhydrolase 1b, catalytic subunit 3
27	AGPAT2	1-acylglycerol-3-phosphate O-acyltransferase 2
28	G6PD	glucose-6-phosphate dehydrogenase
29	ALDOA	aldolase A, fructose-bisphosphate
30	MYO18A	myosin XVIIIA
31	HSPB8	heat shock 22kDa protein 8
32	FST	follistatin
33	BAG1	BCL2-associated athanogene
34	CHMP4C	charged multivesicular body protein 4C
35	CIT	citron (rho-interacting, serine/threonine kinase 21)
36	CAMK4	calcium/calmodulin-dependent protein kinase IV
37	ULK1	unc-51-like kinase 1 (C. elegans)
38	PAK4	p21 protein (Cdc42/Rac)-activated kinase 4
39	ECHDC3	enoyl CoA hydratase domain containing 3
40	HMGCS1	3-hydroxy-3-methylglutaryl-CoA synthase 1 (soluble)
41	SLC9A3R1	solute carrier family 9, subfamily A , member 3 regulator 1
42	PRUNE2	prune homolog 2 (Drosophila)
43	PCYT2	phosphate cytidylyltransferase 2, ethanolamine
44	MVP	major vault protein
45	PNPLA3	patatin-like phospholipase domain containing 3
46	PCK2	phosphoenolpyruvate carboxykinase 2 (mitochondrial)
47	ATP6V0D1	ATPase, H+ transporting, lysosomal 38kDa, V0 subunit d1
48	SUCLA2	succinate-CoA ligase, ADP-forming, beta subunit
49	FADS2	fatty acid desaturase 2
50	PDE4DIP	phosphodiesterase 4D interacting protein
51	ELOVL6	ELOVL fatty acid elongase 6
52	AACS	acetoacetyl-CoA synthetase
53	EFHD1	EF-hand domain family, member D1
54	CRBN	cereblon
55	MBNL3	muscleblind-like splicing regulator 3
56	PDE8A	phosphodiesterase 8A
57	SMAP2	small ArfGAP2
58	RPA1	replication protein A1, 70kDa
59	ACSS3	acyl-CoA synthetase short-chain family member 3
60	FDPS	farnesyl diphosphate synthase
61	DAB1	disabled homolog 1 (Drosophila)
62	ACSS1	acyl-CoA synthetase short-chain family member 1
63	YWHAH	tyrosine 3-monooxygenase
64	NRSN2	neurensin 2
65	RUSC1	RUN and SH3 domain containing 1
66	RAD21	RAD21 homolog (S. pombe)

Database:cellular component Name:intracellular membrane-bounded organelle

C=9587; O=56; E=41.04; R=1.36; rawP=0.0002; adjP=0.0023 ID:GO:0043231

Index	UserID	Gene Name
1	SDSL	serine dehydratase-like
2	ACSL1	acyl-CoA synthetase long-chain family member 1
3	CIT	citron (rho-interacting, serine/threonine kinase 21)
4	FAM129B	family with sequence similarity 129, member B
5	FGD2	FYVE, RhoGEF and PH domain containing 2
6	CAMK4	calcium/calmodulin-dependent protein kinase IV
7	ASS1	argininosuccinate synthase 1
8	TYR	tyrosinase (oculocutaneous albinism IA)
9	ULK1	unc-51-like kinase 1 (C. elegans)
10	PAK4	p21 protein (Cdc42/Rac)-activated kinase 4
11	CLIC2	chloride intracellular channel 2
12	LPIN1	lipin 1
13	ECHDC3	enoyl CoA hydratase domain containing 3
14	HMGCS1	3-hydroxy-3-methylglutaryl-CoA synthase 1 (soluble)
15	ASAP2	ArfGAP with SH3 domain, ankyrin repeat and PH domain 2
16	SLC9A3R1	solute carrier family 9, subfamily A , member 3 regulator 1
17	ACSS2	acyl-CoA synthetase short-chain family member 2
18	PRUNE2	prune homolog 2 (Drosophila)
19	FAM107A	family with sequence similarity 107, member A
20	ABCA2	ATP-binding cassette, sub-family A (ABC1), member 2
21	PCYT2	phosphate cytidylyltransferase 2, ethanolamine
22	PCK2	phosphoenolpyruvate carboxykinase 2 (mitochondrial)
23	PNPLA3	patatin-like phospholipase domain containing 3
24	MVP	major vault protein
25	ATP6V0D1	ATPase, H+ transporting, lysosomal 38kDa, V0 subunit d1
26	DDX1	DEAD (Asp-Glu-Ala-Asp) box helicase 1
27	PPM1K	protein phosphatase, Mg2+/Mn2+ dependent, 1K
28	ACACA	acetyl-CoA carboxylase alpha
29	SUCLA2	succinate-CoA ligase, ADP-forming, beta subunit
30	PDE4DIP	phosphodiesterase 4D interacting protein
31	FADS2	fatty acid desaturase 2
32	ATF4	activating transcription factor 4 (tax-responsive enhancer element B67)
33	ELOVL6	ELOVL fatty acid elongase 6
34	MVD	mevalonate (diphospho) decarboxylase
35	EFHD1	EF-hand domain family, member D1
36	MBNL3	muscleblind-like splicing regulator 3
37	CRBN	cereblon
38	KDM1B	lysine (K)-specific demethylase 1B
39	SMAP2	small ArfGAP2
40	RPA1	replication protein A1, 70kDa
41	OSBPL1A	oxysterol binding protein-like 1A
42	LSS	lanosterol synthase (2,3-oxidosqualene-lanosterol cyclase)
43	ACSS3	acyl-CoA synthetase short-chain family member 3
44	AGPAT2	1-acylglycerol-3-phosphate O-acyltransferase 2
45	G6PD	glucose-6-phosphate dehydrogenase
46	FDPS	farnesyl diphosphate synthase
47	ALDOA	aldolase A, fructose-bisphosphate
48	MYO18A	myosin XVIIIA

49	HSPB8	heat shock 22kDa protein 8
50	FST	follistatin
51	ACSS1	acyl-CoA synthetase short-chain family member 1
52	NRSN2	neurensin 2
53	RUSC1	RUN and SH3 domain containing 1
54	BAG1	BCL2-associated athanogene
55	RAD21	RAD21 homolog (S. pombe)
56	CHMP4C	charged multivesicular body protein 4C

Database:cellular component Name:membrane-bounded organelle ID:GO:0043227
C=9598; O=56; E=41.09; R=1.36; rawP=0.0002; adjP=0.0023

Index	UserID	Gene Name
1	SDSL	serine dehydratase-like
2	ACSL1	acyl-CoA synthetase long-chain family member 1
3	CIT	citron (rho-interacting, serine/threonine kinase 21)
4	FAM129B	family with sequence similarity 129, member B
5	FGD2	FYVE, RhoGEF and PH domain containing 2
6	CAMK4	calcium/calmodulin-dependent protein kinase IV
7	ASS1	argininosuccinate synthase 1
8	TYR	tyrosinase (oculocutaneous albinism IA)
9	ULK1	unc-51-like kinase 1 (C. elegans)
10	PAK4	p21 protein (Cdc42/Rac)-activated kinase 4
11	CLIC2	chloride intracellular channel 2
12	LPIN1	lipin 1
13	ECHDC3	enoyl CoA hydratase domain containing 3
14	HMGCS1	3-hydroxy-3-methylglutaryl-CoA synthase 1 (soluble)
15	ASAP2	ArfGAP with SH3 domain, ankyrin repeat and PH domain 2
16	SLC9A3R1	solute carrier family 9, subfamily A , member 3 regulator 1
17	ACSS2	acyl-CoA synthetase short-chain family member 2
18	PRUNE2	prune homolog 2 (Drosophila)
19	FAM107A	family with sequence similarity 107, member A
20	ABCA2	ATP-binding cassette, sub-family A (ABC1), member 2
21	PCYT2	phosphate cytidylyltransferase 2, ethanolamine
22	PCK2	phosphoenolpyruvate carboxykinase 2 (mitochondrial)
23	PNPLA3	patatin-like phospholipase domain containing 3
24	MVP	major vault protein
25	ATP6V0D1	ATPase, H+ transporting, lysosomal 38kDa, V0 subunit d1
26	DDX1	DEAD (Asp-Glu-Ala-Asp) box helicase 1
27	PPM1K	protein phosphatase, Mg2+/Mn2+ dependent, 1K
28	ACACA	acetyl-CoA carboxylase alpha
29	SUCLA2	succinate-CoA ligase, ADP-forming, beta subunit
30	PDE4DIP	phosphodiesterase 4D interacting protein
31	FADS2	fatty acid desaturase 2
32	ATF4	activating transcription factor 4 (tax-responsive enhancer element B67)
33	ELOVL6	ELOVL fatty acid elongase 6
34	MVD	mevalonate (diphospho) decarboxylase
35	EFHD1	EF-hand domain family, member D1
36	MBNL3	muscleblind-like splicing regulator 3
37	CRBN	cereblon

38	KDM1B	lysine (K)-specific demethylase 1B
39	SMAP2	small ArfGAP2
40	RPA1	replication protein A1, 70kDa
41	OSBPL1A	oxysterol binding protein-like 1A
42	LSS	lanosterol synthase (2,3-oxidosqualene-lanosterol cyclase)
43	ACSS3	acyl-CoA synthetase short-chain family member 3
44	AGPAT2	1-acylglycerol-3-phosphate O-acyltransferase 2
45	G6PD	glucose-6-phosphate dehydrogenase
46	FDPS	farnesyl diphosphate synthase
47	ALDOA	aldolase A, fructose-bisphosphate
48	MYO18A	myosin XVIIIA
49	HSPB8	heat shock 22kDa protein 8
50	FST	follistatin
51	ACSS1	acyl-CoA synthetase short-chain family member 1
52	NRSN2	neurensin 2
53	RUSC1	RUN and SH3 domain containing 1
54	BAG1	BCL2-associated athanogene
55	RAD21	RAD21 homolog (S. pombe)
56	CHMP4C	charged multivesicular body protein 4C

Database:cellular component Name:intracellular ID:GO:0005622
C=12564; O=66; E=53.78; R=1.23; rawP=0.0003; adjP=0.0029

Index	UserID	Gene Name
1	SDSL	serine dehydratase-like
2	ACSL1	acyl-CoA synthetase long-chain family member 1
3	FAM129B	family with sequence similarity 129, member B
4	FGD2	FYVE, RhoGEF and PH domain containing 2
5	ASS1	argininosuccinate synthase 1
6	TYR	tyrosinase (oculocutaneous albinism IA)
7	CLIC2	chloride intracellular channel 2
8	LPIN1	lipin 1
9	CDC42EP4	CDC42 effector protein (Rho GTPase binding) 4
10	ASAP2	ArfGAP with SH3 domain, ankyrin repeat and PH domain 2
11	ARHGAP18	Rho GTPase activating protein 18
12	ACSS2	acyl-CoA synthetase short-chain family member 2
13	RNF41	ring finger protein 41
14	FAM107A	family with sequence similarity 107, member A
15	ABCA2	ATP-binding cassette, sub-family A (ABC1), member 2
16	EPB41L4B	erythrocyte membrane protein band 4.1 like 4B
17	PPM1K	protein phosphatase, Mg2+/Mn2+ dependent, 1K
18	DDX1	DEAD (Asp-Glu-Ala-Asp) box helicase 1
19	ACACA	acetyl-CoA carboxylase alpha
20	ATF4	activating transcription factor 4 (tax-responsive enhancer element B67)
21	RASEF	RAS and EF-hand domain containing
22	MVD	mevalonate (diphospho) decarboxylase
23	KDM1B	lysine (K)-specific demethylase 1B
24	OSBPL1A	oxysterol binding protein-like 1A
25	LSS	lanosterol synthase (2,3-oxidosqualene-lanosterol cyclase)
26	PAFAH1B3	platelet-activating factor acetylhydrolase 1b, catalytic subunit 3
27	AGPAT2	1-acylglycerol-3-phosphate O-acyltransferase 2

28	G6PD	glucose-6-phosphate dehydrogenase
29	ALDOA	aldolase A, fructose-bisphosphate
30	MYO18A	myosin XVIIIA
31	HSPB8	heat shock 22kDa protein 8
32	FST	follistatin
33	BAG1	BCL2-associated athanogene
34	CHMP4C	charged multivesicular body protein 4C
35	CIT	citron (rho-interacting, serine/threonine kinase 21)
36	CAMK4	calcium/calmodulin-dependent protein kinase IV
37	ULK1	unc-51-like kinase 1 (C. elegans)
38	PAK4	p21 protein (Cdc42/Rac)-activated kinase 4
39	ECHDC3	enoyl CoA hydratase domain containing 3
40	HMGCS1	3-hydroxy-3-methylglutaryl-CoA synthase 1 (soluble)
41	SLC9A3R1	solute carrier family 9, subfamily A , member 3 regulator 1
42	PRUNE2	prune homolog 2 (Drosophila)
43	PCYT2	phosphate cytidylyltransferase 2, ethanolamine
44	MVP	major vault protein
45	PNPLA3	patatin-like phospholipase domain containing 3
46	PCK2	phosphoenolpyruvate carboxykinase 2 (mitochondrial)
47	ATP6V0D1	ATPase, H+ transporting, lysosomal 38kDa, V0 subunit d1
48	SUCLA2	succinate-CoA ligase, ADP-forming, beta subunit
49	FADS2	fatty acid desaturase 2
50	PDE4DIP	phosphodiesterase 4D interacting protein
51	ELOVL6	ELOVL fatty acid elongase 6
52	AACS	acetoacetyl-CoA synthetase
53	EFHD1	EF-hand domain family, member D1
54	CRBN	cereblon
55	MBNL3	muscleblind-like splicing regulator 3
56	PDE8A	phosphodiesterase 8A
57	SMAP2	small ArfGAP2
58	RPA1	replication protein A1, 70kDa
59	ACSS3	acyl-CoA synthetase short-chain family member 3
60	FDPS	farnesyl diphosphate synthase
61	DAB1	disabled homolog 1 (Drosophila)
62	ACSS1	acyl-CoA synthetase short-chain family member 1
63	YWHAH	tyrosine 3-monooxygenase
64	NRSN2	neurensin 2
65	RUSC1	RUN and SH3 domain containing 1
66	RAD21	RAD21 homolog (S. pombe)

Database:cellular component Name:intracellular organelle ID:GO:0043229
C=10636; O=58; E=45.53; R=1.27; rawP=0.0013; adjP=0.0102

Index	UserID	Gene Name
1	SDSL	serine dehydratase-like
2	ACSL1	acyl-CoA synthetase long-chain family member 1
3	CIT	citron (rho-interacting, serine/threonine kinase 21)
4	FAM129B	family with sequence similarity 129, member B
5	FGD2	FYVE, RhoGEF and PH domain containing 2
6	CAMK4	calcium/calmodulin-dependent protein kinase IV

7	ASS1	argininosuccinate synthase 1
8	TYR	tyrosinase (oculocutaneous albinism IA)
9	ULK1	unc-51-like kinase 1 (C. elegans)
10	PAK4	p21 protein (Cdc42/Rac)-activated kinase 4
11	CLIC2	chloride intracellular channel 2
12	LPIN1	lipin 1
13	CDC42EP4	CDC42 effector protein (Rho GTPase binding) 4
14	ECHDC3	enoyl CoA hydratase domain containing 3
15	HMGCS1	3-hydroxy-3-methylglutaryl-CoA synthase 1 (soluble)
16	ASAP2	ArfGAP with SH3 domain, ankyrin repeat and PH domain 2
17	SLC9A3R1	solute carrier family 9, subfamily A , member 3 regulator 1
18	ACSS2	acyl-CoA synthetase short-chain family member 2
19	PRUNE2	prune homolog 2 (Drosophila)
20	FAM107A	family with sequence similarity 107, member A
21	ABCA2	ATP-binding cassette, sub-family A (ABC1), member 2
22	PCYT2	phosphate cytidylyltransferase 2, ethanolamine
23	EPB41L4B	erythrocyte membrane protein band 4.1 like 4B
24	PCK2	phosphoenolpyruvate carboxykinase 2 (mitochondrial)
25	PNPLA3	patatin-like phospholipase domain containing 3
26	MVP	major vault protein
27	ATP6V0D1	ATPase, H+ transporting, lysosomal 38kDa, V0 subunit d1
28	DDX1	DEAD (Asp-Glu-Ala-Asp) box helicase 1
29	PPM1K	protein phosphatase, Mg2+/Mn2+ dependent, 1K
30	ACACA	acetyl-CoA carboxylase alpha
31	SUCLA2	succinate-CoA ligase, ADP-forming, beta subunit
32	PDE4DIP	phosphodiesterase 4D interacting protein
33	FADS2	fatty acid desaturase 2
34	ATF4	activating transcription factor 4 (tax-responsive enhancer element B67)
35	ELOVL6	ELOVL fatty acid elongase 6
36	MVD	mevalonate (diphospho) decarboxylase
37	EFHD1	EF-hand domain family, member D1
38	MBNL3	muscleblind-like splicing regulator 3
39	CRBN	cereblon
40	KDM1B	lysine (K)-specific demethylase 1B
41	SMAP2	small ArfGAP2
42	RPA1	replication protein A1, 70kDa
43	OSBPL1A	oxysterol binding protein-like 1A
44	LSS	lanosterol synthase (2,3-oxidosqualene-lanosterol cyclase)
45	ACSS3	acyl-CoA synthetase short-chain family member 3
46	AGPAT2	1-acylglycerol-3-phosphate O-acyltransferase 2
47	G6PD	glucose-6-phosphate dehydrogenase
48	FDPS	farnesyl diphosphate synthase
49	ALDOA	aldolase A, fructose-bisphosphate
50	MYO18A	myosin XVIIIA
51	HSPB8	heat shock 22kDa protein 8
52	FST	follistatin
53	ACSS1	acyl-CoA synthetase short-chain family member 1
54	NRSN2	neurensin 2
55	RUSC1	RUN and SH3 domain containing 1
56	BAG1	BCL2-associated athanogene

57	RAD21	RAD21 homolog (S. pombe)
58	CHMP4C	charged multivesicular body protein 4C

Database:cellular component Name:organelle ID:GO:0043226

C=10651; O=58; E=45.59; R=1.27; rawP=0.0014; adjP=0.0102

Index	UserID	Gene Name
1	SDSL	serine dehydratase-like
2	ACSL1	acyl-CoA synthetase long-chain family member 1
3	CIT	citron (rho-interacting, serine/threonine kinase 21)
4	FAM129B	family with sequence similarity 129, member B
5	FGD2	FYVE, RhoGEF and PH domain containing 2
6	CAMK4	calcium/calmodulin-dependent protein kinase IV
7	ASS1	argininosuccinate synthase 1
8	TYR	tyrosinase (oculocutaneous albinism IA)
9	ULK1	unc-51-like kinase 1 (C. elegans)
10	PAK4	p21 protein (Cdc42/Rac)-activated kinase 4
11	CLIC2	chloride intracellular channel 2
12	LPIN1	lipin 1
13	CDC42EP4	CDC42 effector protein (Rho GTPase binding) 4
14	ECHDC3	enoyl CoA hydratase domain containing 3
15	HMGCS1	3-hydroxy-3-methylglutaryl-CoA synthase 1 (soluble)
16	ASAP2	ArfGAP with SH3 domain, ankyrin repeat and PH domain 2
17	SLC9A3R1	solute carrier family 9, subfamily A , member 3 regulator 1
18	ACSS2	acyl-CoA synthetase short-chain family member 2
19	PRUNE2	prune homolog 2 (Drosophila)
20	FAM107A	family with sequence similarity 107, member A
21	ABCA2	ATP-binding cassette, sub-family A (ABC1), member 2
22	PCYT2	phosphate cytidylyltransferase 2, ethanolamine
23	EPB41L4B	erythrocyte membrane protein band 4.1 like 4B
24	PCK2	phosphoenolpyruvate carboxykinase 2 (mitochondrial)
25	PNPLA3	patatin-like phospholipase domain containing 3
26	MVP	major vault protein
27	ATP6V0D1	ATPase, H+ transporting, lysosomal 38kDa, V0 subunit d1
28	DDX1	DEAD (Asp-Glu-Ala-Asp) box helicase 1
29	PPM1K	protein phosphatase, Mg2+/Mn2+ dependent, 1K
30	ACACA	acetyl-CoA carboxylase alpha
31	SUCLA2	succinate-CoA ligase, ADP-forming, beta subunit
32	PDE4DIP	phosphodiesterase 4D interacting protein
33	FADS2	fatty acid desaturase 2
34	ATF4	activating transcription factor 4 (tax-responsive enhancer element B67)
35	ELOVL6	ELOVL fatty acid elongase 6
36	MVD	mevalonate (diphospho) decarboxylase
37	EFHD1	EF-hand domain family, member D1
38	MBNL3	muscleblind-like splicing regulator 3
39	CRBN	cereblon
40	KDM1B	lysine (K)-specific demethylase 1B
41	SMAP2	small ArfGAP2
42	RPA1	replication protein A1, 70kDa
43	OSBPL1A	oxysterol binding protein-like 1A
44	LSS	lanosterol synthase (2,3-oxidosqualene-lanosterol cyclase)

45	ACSS3	acyl-CoA synthetase short-chain family member 3
46	AGPAT2	1-acylglycerol-3-phosphate O-acyltransferase 2
47	G6PD	glucose-6-phosphate dehydrogenase
48	FDPS	farnesyl diphosphate synthase
49	ALDOA	aldolase A, fructose-bisphosphate
50	MYO18A	myosin XVIIIA
51	HSPB8	heat shock 22kDa protein 8
52	FST	follistatin
53	ACSS1	acyl-CoA synthetase short-chain family member 1
54	NRSN2	neurensin 2
55	RUSC1	RUN and SH3 domain containing 1
56	BAG1	BCL2-associated athanogene
57	RAD21	RAD21 homolog (S. pombe)
58	CHMP4C	charged multivesicular body protein 4C

Database:cellular component Name:cytosol ID:GO:0005829
C=2372; O=20; E=10.15; R=1.97; rawP=0.0018; adjP=0.0104

Index	UserID	Gene Name
1	AACS	acetoacetyl-CoA synthetase
2	MVD	mevalonate (diphospho) decarboxylase
3	FAM129B	family with sequence similarity 129, member B
4	FGD2	FYVE, RhoGEF and PH domain containing 2
5	CAMK4	calcium/calmodulin-dependent protein kinase IV
6	ASS1	argininosuccinate synthase 1
7	PDE8A	phosphodiesterase 8A
8	ULK1	unc-51-like kinase 1 (C. elegans)
9	LPIN1	lipin 1
10	HMGCS1	3-hydroxy-3-methylglutaryl-CoA synthase 1 (soluble)
11	ARHGAP18	Rho GTPase activating protein 18
12	PAFAH1B3	platelet-activating factor acetylhydrolase 1b, catalytic subunit 3
13	ACSS2	acyl-CoA synthetase short-chain family member 2
14	RNF41	ring finger protein 41
15	G6PD	glucose-6-phosphate dehydrogenase
16	FDPS	farnesyl diphosphate synthase
17	ALDOA	aldolase A, fructose-bisphosphate
18	ACACA	acetyl-CoA carboxylase alpha
19	BAG1	BCL2-associated athanogene
20	CHMP4C	charged multivesicular body protein 4C

Database:cellular component Name:vacuole ID:GO:0005773
C=408; O=7; E=1.75; R=4.01; rawP=0.0018; adjP=0.0104

Index	UserID	Gene Name
1	TYR	tyrosinase (oculocutaneous albinism IA)
2	ASS1	argininosuccinate synthase 1
3	ULK1	unc-51-like kinase 1 (C. elegans)
4	ATP6V0D1	ATPase, H+ transporting, lysosomal 38kDa, V0 subunit d1
5	CIT	citron (rho-interacting, serine/threonine kinase 21)
6	OSBPL1A	oxysterol binding protein-like 1A

7	ABCA2	ATP-binding cassette, sub-family A (ABC1), member 2

Database:cellular component Name:actin cytoskeleton ID:GO:0015629
C=365; O=6; E=1.56; R=3.84; rawP=0.0047; adjP=0.0248

Index	UserID	Gene Name
1	SLC9A3R1	solute carrier family 9, subfamily A , member 3 regulator 1
2	CIT	citron (rho-interacting, serine/threonine kinase 21)
3	MYO18A	myosin XVIIIA
4	ALDOA	aldolase A, fructose-bisphosphate
5	CDC42EP4	CDC42 effector protein (Rho GTPase binding) 4
6	RPA1	replication protein A1, 70kDa

Database:cellular component Name:endomembrane system ID:GO:0012505
C=1771; O=15; E=7.58; R=1.98; rawP=0.0073; adjP=0.0353

Index	UserID	Gene Name
1	FADS2	fatty acid desaturase 2
2	LSS	lanosterol synthase (2,3-oxidosqualene-lanosterol cyclase)
3	ASAP2	ArfGAP with SH3 domain, ankyrin repeat and PH domain 2
4	ACSL1	acyl-CoA synthetase long-chain family member 1
5	SLC9A3R1	solute carrier family 9, subfamily A , member 3 regulator 1
6	AGPAT2	1-acylglycerol-3-phosphate O-acyltransferase 2
7	ELOVL6	ELOVL fatty acid elongase 6
8	TYR	tyrosinase (oculocutaneous albinism IA)
9	ULK1	unc-51-like kinase 1 (C. elegans)
10	PCYT2	phosphate cytidylyltransferase 2, ethanolamine
11	PNPLA3	patatin-like phospholipase domain containing 3
12	MVP	major vault protein
13	ATP6V0D1	ATPase, H+ transporting, lysosomal 38kDa, V0 subunit d1
14	CDC42EP4	CDC42 effector protein (Rho GTPase binding) 4
15	LPIN1	lipin 1

Database:cellular component Name:apical part of cell ID:GO:0045177
C=294; O=5; E=1.26; R=3.97; rawP=0.0085; adjP=0.0379

Index	UserID	Gene Name
1	SLC6A20	solute carrier family 6 (proline IMINO transporter), member 20
2	EPB41L4B	erythrocyte membrane protein band 4.1 like 4B
3	SLC9A3R1	solute carrier family 9, subfamily A , member 3 regulator 1
4	ATP6V0D1	ATPase, H+ transporting, lysosomal 38kDa, V0 subunit d1
5	SLC9A4	solute carrier family 9, subfamily A member 4

Database:cellular component Name:intracellular organelle part ID:GO:0044446
C=6725; O=39; E=28.79; R=1.35; rawP=0.0105; adjP=0.0406

Index	UserID	Gene Name
1	ACSL1	acyl-CoA synthetase long-chain family member 1
2	FGD2	FYVE, RhoGEF and PH domain containing 2
3	CAMK4	calcium/calmodulin-dependent protein kinase IV
4	ASS1	argininosuccinate synthase 1

5	TYR	tyrosinase (oculocutaneous albinism IA)
6	ULK1	unc-51-like kinase 1 (C. elegans)
7	LPIN1	lipin 1
8	HMGCS1	3-hydroxy-3-methylglutaryl-CoA synthase 1 (soluble)
9	ASAP2	ArfGAP with SH3 domain, ankyrin repeat and PH domain 2
10	SLC9A3R1	solute carrier family 9, subfamily A , member 3 regulator 1
11	ACSS2	acyl-CoA synthetase short-chain family member 2
12	ABCA2	ATP-binding cassette, sub-family A (ABC1), member 2
13	PCYT2	phosphate cytidylyltransferase 2, ethanolamine
14	PCK2	phosphoenolpyruvate carboxykinase 2 (mitochondrial)
15	PNPLA3	patatin-like phospholipase domain containing 3
16	MVP	major vault protein
17	ATP6V0D1	ATPase, H+ transporting, lysosomal 38kDa, V0 subunit d1
18	DDX1	DEAD (Asp-Glu-Ala-Asp) box helicase 1
19	PPM1K	protein phosphatase, Mg2+/Mn2+ dependent, 1K
20	SUCLA2	succinate-CoA ligase, ADP-forming, beta subunit
21	FADS2	fatty acid desaturase 2
22	PDE4DIP	phosphodiesterase 4D interacting protein
23	ATF4	activating transcription factor 4 (tax-responsive enhancer element B67)
24	ELOVL6	ELOVL fatty acid elongase 6
25	EFHD1	EF-hand domain family, member D1
26	CRBN	cereblon
27	SMAP2	small ArfGAP2
28	RPA1	replication protein A1, 70kDa
29	OSBPL1A	oxysterol binding protein-like 1A
30	LSS	lanosterol synthase (2,3-oxidosqualene-lanosterol cyclase)
31	AGPAT2	1-acylglycerol-3-phosphate O-acyltransferase 2
32	G6PD	glucose-6-phosphate dehydrogenase
33	FDPS	farnesyl diphosphate synthase
34	ALDOA	aldolase A, fructose-bisphosphate
35	MYO18A	myosin XVIIIA
36	ACSS1	acyl-CoA synthetase short-chain family member 1
37	RUSC1	RUN and SH3 domain containing 1
38	RAD21	RAD21 homolog (S. pombe)
39	CHMP4C	charged multivesicular body protein 4C

Database:cellular component Name:organelle membrane ID:GO:0031090
C=2373; O=18; E=10.16; R=1.77; rawP=0.0099; adjP=0.0406

Index	UserID	Gene Name
1	FADS2	fatty acid desaturase 2
2	ACSL1	acyl-CoA synthetase long-chain family member 1
3	ELOVL6	ELOVL fatty acid elongase 6
4	EFHD1	EF-hand domain family, member D1
5	FGD2	FYVE, RhoGEF and PH domain containing 2
6	ASS1	argininosuccinate synthase 1
7	TYR	tyrosinase (oculocutaneous albinism IA)
8	ULK1	unc-51-like kinase 1 (C. elegans)
9	LPIN1	lipin 1
10	OSBPL1A	oxysterol binding protein-like 1A

11	LSS	lanosterol synthase (2,3-oxidosqualene-lanosterol cyclase)
12	ASAP2	ArfGAP with SH3 domain, ankyrin repeat and PH domain 2
13	AGPAT2	1-acylglycerol-3-phosphate O-acyltransferase 2
14	ABCA2	ATP-binding cassette, sub-family A (ABC1), member 2
15	PCYT2	phosphate cytidylyltransferase 2, ethanolamine
16	PNPLA3	patatin-like phospholipase domain containing 3
17	ATP6V0D1	ATPase, H+ transporting, lysosomal 38kDa, V0 subunit d1
18	CHMP4C	charged multivesicular body protein 4C

Database:cellular component Name:mitochondrion ID:GO:0005739

C=1525; O=13; E=6.53; R=1.99; rawP=0.0120; adjP=0.0435

Index	UserID	Gene Name
1	SUCLA2	succinate-CoA ligase, ADP-forming, beta subunit
2	ECHDC3	enoyl CoA hydratase domain containing 3
3	SDSL	serine dehydratase-like
4	ACSS3	acyl-CoA synthetase short-chain family member 3
5	ACSL1	acyl-CoA synthetase long-chain family member 1
6	FDPS	farnesyl diphosphate synthase
7	EFHD1	EF-hand domain family, member D1
8	ASS1	argininosuccinate synthase 1
9	PCK2	phosphoenolpyruvate carboxykinase 2 (mitochondrial)
10	ACSS1	acyl-CoA synthetase short-chain family member 1
11	PPM1K	protein phosphatase, Mg2+/Mn2+ dependent, 1K
12	ACACA	acetyl-CoA carboxylase alpha
13	BAG1	BCL2-associated athanogene

Database:cellular component Name:organelle part ID:GO:0044422

C=6812; O=39; E=29.16; R=1.34; rawP=0.0134; adjP=0.0457

Index	UserID	Gene Name
1	ACSL1	acyl-CoA synthetase long-chain family member 1
2	FGD2	FYVE, RhoGEF and PH domain containing 2
3	CAMK4	calcium/calmodulin-dependent protein kinase IV
4	ASS1	argininosuccinate synthase 1
5	TYR	tyrosinase (oculocutaneous albinism IA)
6	ULK1	unc-51-like kinase 1 (C. elegans)
7	LPIN1	lipin 1
8	HMGCS1	3-hydroxy-3-methylglutaryl-CoA synthase 1 (soluble)
9	ASAP2	ArfGAP with SH3 domain, ankyrin repeat and PH domain 2
10	SLC9A3R1	solute carrier family 9, subfamily A , member 3 regulator 1
11	ACSS2	acyl-CoA synthetase short-chain family member 2
12	ABCA2	ATP-binding cassette, sub-family A (ABC1), member 2
13	PCYT2	phosphate cytidylyltransferase 2, ethanolamine
14	PCK2	phosphoenolpyruvate carboxykinase 2 (mitochondrial)
15	PNPLA3	patatin-like phospholipase domain containing 3
16	MVP	major vault protein
17	ATP6V0D1	ATPase, H+ transporting, lysosomal 38kDa, V0 subunit d1
18	DDX1	DEAD (Asp-Glu-Ala-Asp) box helicase 1
19	PPM1K	protein phosphatase, Mg2+/Mn2+ dependent, 1K

20	SUCLA2	succinate-CoA ligase, ADP-forming, beta subunit
21	FADS2	fatty acid desaturase 2
22	PDE4DIP	phosphodiesterase 4D interacting protein
23	ATF4	activating transcription factor 4 (tax-responsive enhancer element B67)
24	ELOVL6	ELOVL fatty acid elongase 6
25	EFHD1	EF-hand domain family, member D1
26	CRBN	cereblon
27	SMAP2	small ArfGAP2
28	RPA1	replication protein A1, 70kDa
29	OSBPL1A	oxysterol binding protein-like 1A
30	LSS	lanosterol synthase (2,3-oxidosqualene-lanosterol cyclase)
31	AGPAT2	1-acylglycerol-3-phosphate O-acyltransferase 2
32	G6PD	glucose-6-phosphate dehydrogenase
33	FDPS	farnesyl diphosphate synthase
34	ALDOA	aldolase A, fructose-bisphosphate
35	MYO18A	myosin XVIIIA
36	ACSS1	acyl-CoA synthetase short-chain family member 1
37	RUSC1	RUN and SH3 domain containing 1
38	RAD21	RAD21 homolog (S. pombe)
39	CHMP4C	charged multivesicular body protein 4C

Database:cellular component Name:endoplasmic reticulum membrane ID:GO:0005789
C=772; O=8; E=3.30; R=2.42; rawP=0.0171; adjP=0.0496

Index	UserID	Gene Name
1	FADS2	fatty acid desaturase 2
2	LSS	lanosterol synthase (2,3-oxidosqualene-lanosterol cyclase)
3	ACSL1	acyl-CoA synthetase long-chain family member 1
4	AGPAT2	1-acylglycerol-3-phosphate O-acyltransferase 2
5	ELOVL6	ELOVL fatty acid elongase 6
6	PCYT2	phosphate cytidylyltransferase 2, ethanolamine
7	PNPLA3	patatin-like phospholipase domain containing 3
8	LPIN1	lipin 1

Database:cellular component Name:cell part ID:GO:0044464
C=14643; O=69; E=62.68; R=1.10; rawP=0.0170; adjP=0.0496

Index	UserID	Gene Name
1	SDSL	serine dehydratase-like
2	ACSL1	acyl-CoA synthetase long-chain family member 1
3	FAM129B	family with sequence similarity 129, member B
4	FGD2	FYVE, RhoGEF and PH domain containing 2
5	ASS1	argininosuccinate synthase 1
6	TYR	tyrosinase (oculocutaneous albinism IA)
7	CLIC2	chloride intracellular channel 2
8	LPIN1	lipin 1
9	CDC42EP4	CDC42 effector protein (Rho GTPase binding) 4
10	ASAP2	ArfGAP with SH3 domain, ankyrin repeat and PH domain 2
11	ARHGAP18	Rho GTPase activating protein 18
12	ACSS2	acyl-CoA synthetase short-chain family member 2
13	RNF41	ring finger protein 41

14	FAM107A	family with sequence similarity 107, member A
15	ABCA2	ATP-binding cassette, sub-family A (ABC1), member 2
16	EPB41L4B	erythrocyte membrane protein band 4.1 like 4B
17	PPM1K	protein phosphatase, Mg2+/Mn2+ dependent, 1K
18	DDX1	DEAD (Asp-Glu-Ala-Asp) box helicase 1
19	ACACA	acetyl-CoA carboxylase alpha
20	ATF4	activating transcription factor 4 (tax-responsive enhancer element B67)
21	RASEF	RAS and EF-hand domain containing
22	MVD	mevalonate (diphospho) decarboxylase
23	KDM1B	lysine (K)-specific demethylase 1B
24	OSBPL1A	oxysterol binding protein-like 1A
25	LSS	lanosterol synthase (2,3-oxidosqualene-lanosterol cyclase)
26	AZGP1	alpha-2-glycoprotein 1, zinc-binding
27	PAFAH1B3	platelet-activating factor acetylhydrolase 1b, catalytic subunit 3
28	AGPAT2	1-acylglycerol-3-phosphate O-acyltransferase 2
29	G6PD	glucose-6-phosphate dehydrogenase
30	ALDOA	aldolase A, fructose-bisphosphate
31	MYO18A	myosin XVIIIA
32	HSPB8	heat shock 22kDa protein 8
33	SLC6A20	solute carrier family 6 (proline IMINO transporter), member 20
34	FST	follistatin
35	BAG1	BCL2-associated athanogene
36	CHMP4C	charged multivesicular body protein 4C
37	CIT	citron (rho-interacting, serine/threonine kinase 21)
38	CAMK4	calcium/calmodulin-dependent protein kinase IV
39	ULK1	unc-51-like kinase 1 (C. elegans)
40	PAK4	p21 protein (Cdc42/Rac)-activated kinase 4
41	ECHDC3	enoyl CoA hydratase domain containing 3
42	HMGCS1	3-hydroxy-3-methylglutaryl-CoA synthase 1 (soluble)
43	SLC9A3R1	solute carrier family 9, subfamily A , member 3 regulator 1
44	SLC9A4	solute carrier family 9, subfamily A member 4
45	PRUNE2	prune homolog 2 (Drosophila)
46	PCYT2	phosphate cytidylyltransferase 2, ethanolamine
47	MVP	major vault protein
48	PNPLA3	patatin-like phospholipase domain containing 3
49	PCK2	phosphoenolpyruvate carboxykinase 2 (mitochondrial)
50	ATP6V0D1	ATPase, H+ transporting, lysosomal 38kDa, V0 subunit d1
51	SUCLA2	succinate-CoA ligase, ADP-forming, beta subunit
52	FADS2	fatty acid desaturase 2
53	PDE4DIP	phosphodiesterase 4D interacting protein
54	ELOVL6	ELOVL fatty acid elongase 6
55	AACS	acetoacetyl-CoA synthetase
56	EFHD1	EF-hand domain family, member D1
57	CRBN	cereblon
58	MBNL3	muscleblind-like splicing regulator 3
59	PDE8A	phosphodiesterase 8A
60	SMAP2	small ArfGAP2
61	RPA1	replication protein A1, 70kDa
62	ACSS3	acyl-CoA synthetase short-chain family member 3
63	FDPS	farnesyl diphosphate synthase

64	DAB1	disabled homolog 1 (Drosophila)
65	YWHAH	tyrosine 3-monooxygenase
66	ACSS1	acyl-CoA synthetase short-chain family member 1
67	NRSN2	neurensin 2
68	RUSC1	RUN and SH3 domain containing 1
69	RAD21	RAD21 homolog (S. pombe)

Database:cellular component Name:cell ID:GO:0005623
C=14644; O=69; E=62.69; R=1.10; rawP=0.0170; adjP=0.0496

Index	UserID	Gene Name
1	SDSL	serine dehydratase-like
2	ACSL1	acyl-CoA synthetase long-chain family member 1
3	FAM129B	family with sequence similarity 129, member B
4	FGD2	FYVE, RhoGEF and PH domain containing 2
5	ASS1	argininosuccinate synthase 1
6	TYR	tyrosinase (oculocutaneous albinism IA)
7	CLIC2	chloride intracellular channel 2
8	LPIN1	lipin 1
9	CDC42EP4	CDC42 effector protein (Rho GTPase binding) 4
10	ASAP2	ArfGAP with SH3 domain, ankyrin repeat and PH domain 2
11	ARHGAP18	Rho GTPase activating protein 18
12	ACSS2	acyl-CoA synthetase short-chain family member 2
13	RNF41	ring finger protein 41
14	FAM107A	family with sequence similarity 107, member A
15	ABCA2	ATP-binding cassette, sub-family A (ABC1), member 2
16	EPB41L4B	erythrocyte membrane protein band 4.1 like 4B
17	PPM1K	protein phosphatase, Mg2+/Mn2+ dependent, 1K
18	DDX1	DEAD (Asp-Glu-Ala-Asp) box helicase 1
19	ACACA	acetyl-CoA carboxylase alpha
20	ATF4	activating transcription factor 4 (tax-responsive enhancer element B67)
21	RASEF	RAS and EF-hand domain containing
22	MVD	mevalonate (diphospho) decarboxylase
23	KDM1B	lysine (K)-specific demethylase 1B
24	OSBPL1A	oxysterol binding protein-like 1A
25	LSS	lanosterol synthase (2,3-oxidosqualene-lanosterol cyclase)
26	AZGP1	alpha-2-glycoprotein 1, zinc-binding
27	PAFAH1B3	platelet-activating factor acetylhydrolase 1b, catalytic subunit 3
28	AGPAT2	1-acylglycerol-3-phosphate O-acyltransferase 2
29	G6PD	glucose-6-phosphate dehydrogenase
30	ALDOA	aldolase A, fructose-bisphosphate
31	MYO18A	myosin XVIIIA
32	HSPB8	heat shock 22kDa protein 8
33	SLC6A20	solute carrier family 6 (proline IMINO transporter), member 20
34	FST	follistatin
35	BAG1	BCL2-associated athanogene
36	CHMP4C	charged multivesicular body protein 4C
37	CIT	citron (rho-interacting, serine/threonine kinase 21)
38	CAMK4	calcium/calmodulin-dependent protein kinase IV
39	ULK1	unc-51-like kinase 1 (C. elegans)

40	PAK4	p21 protein (Cdc42/Rac)-activated kinase 4
41	ECHDC3	enoyl CoA hydratase domain containing 3
42	HMGCS1	3-hydroxy-3-methylglutaryl-CoA synthase 1 (soluble)
43	SLC9A3R1	solute carrier family 9, subfamily A , member 3 regulator 1
44	SLC9A4	solute carrier family 9, subfamily A member 4
45	PRUNE2	prune homolog 2 (Drosophila)
46	PCYT2	phosphate cytidylyltransferase 2, ethanolamine
47	MVP	major vault protein
48	PNPLA3	patatin-like phospholipase domain containing 3
49	PCK2	phosphoenolpyruvate carboxykinase 2 (mitochondrial)
50	ATP6V0D1	ATPase, H+ transporting, lysosomal 38kDa, V0 subunit d1
51	SUCLA2	succinate-CoA ligase, ADP-forming, beta subunit
52	FADS2	fatty acid desaturase 2
53	PDE4DIP	phosphodiesterase 4D interacting protein
54	ELOVL6	ELOVL fatty acid elongase 6
55	AACS	acetoacetyl-CoA synthetase
56	EFHD1	EF-hand domain family, member D1
57	CRBN	cereblon
58	MBNL3	muscleblind-like splicing regulator 3
59	PDE8A	phosphodiesterase 8A
60	SMAP2	small ArfGAP2
61	RPA1	replication protein A1, 70kDa
62	ACSS3	acyl-CoA synthetase short-chain family member 3
63	FDPS	farnesyl diphosphate synthase
64	DAB1	disabled homolog 1 (Drosophila)
65	YWHAH	tyrosine 3-monooxygenase
66	ACSS1	acyl-CoA synthetase short-chain family member 1
67	NRSN2	neurensin 2
68	RUSC1	RUN and SH3 domain containing 1
69	RAD21	RAD21 homolog (S. pombe)

Database:Wikipathways pathway Name:SREBP signalling ID:WP1982

C=83; O=6; E=0.15; R=40.49; rawP=8.99e-09; adjP=8.99e-09

Index	UserID	Gene Name
1	LSS	lanosterol synthase (2,3-oxidosqualene-lanosterol cyclase)
2	HMGCS1	3-hydroxy-3-methylglutaryl-CoA synthase 1 (soluble)
3	ACSS1	acyl-CoA synthetase short-chain family member 1
4	MVD	mevalonate (diphospho) decarboxylase
5	ACACA	acetyl-CoA carboxylase alpha
6	FDPS	farnesyl diphosphate synthase

Supplementary File S6

Title of data: Validation of RNAseq data by qRT-PCR

Description of data: Correlation between fold changes in the mRNA abundance (log_2 transformed) of genes quantified by RNA-seq and qRT-PCR. The plotted genes correspond to a subset of 4 downregulated (*ACACA*, *ACSS1*, *ACSS2* and *LPIN1*), 4 upregulated (*APP*, *ATF3*, *KLF6* and *PPT1*) and 4 non-differentially expressed genes (*GPAM*, *GPAT4*, *SCD* and *SREBF1*).

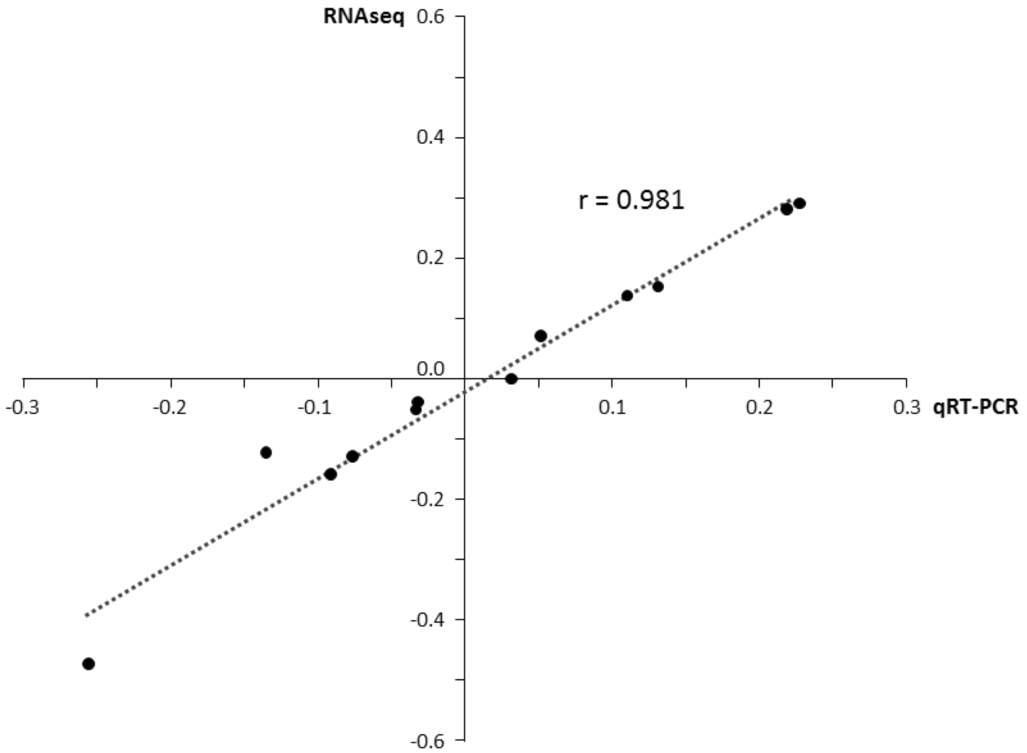

RNAseq
0.6
0.4
0.2
0.0
-0.2
-0.4
-0.6
-0.3
-0.2
-0.1
0.1
0.2
0.3
qRT-PCR
r = 0.981

Supplementary File S7

Title of data: Validation of RNAseq data by qRT-PCR

Description of data: Primer sequences and qRT-PCR performance of genes used to validate the RNA-seq data. To account for variations in RNA integrity and quantification and cDNA synthesis, mRNA abundance was normalized using the geometric mean of 3 reference genes: eukaryotic translation initiation factor 3 subunit K (*EIF3K*), peptidylprolyl isomerase A (*PPIA*) and ubiquitiously expressed prefoldinlike chaperone (*UXT*), which have been identified as suitable internal controls in lactating ruminants (Bionaz and Loor, 2007; Bonnet et al., 2013).

Gene	Accession#	Primers[1]	Nucleotide sequence (5' → 3')	Source[2]	Cq[3]	Slope[4]	(R^2)[5]	Efficiency[6]
ACACA	NM_001009256.1	F.2201	ACCATGCTGGGAGTTGTCTGT	(1)	21.3 ± 0.35	−3.453	0.996	1.95
		R.2319	AGAAGTGTATGAGCAGAGAGGACTTG					
ACSS1	XM_015099680.1	F.683	CGAAGCCATAAAGATCTGTCCAT	(1)	26.8 ± 0.26	−3.464	0.998	1.94
		R.789	CCATCTCCTGCTCAAGAGAAACA					
ACSS2	XM_004014514.3	F.480	AGTGTGTCGGTTCAGCAATG	(2)	26.5 ± 0.44	−3.903	0.984	1.80
		R.623	GCAAACACAATGGAGTGCAG					
APP	XM_004002804.3	F.1198	AGACCACCCAGGAACCTCTT	(3)	28.5 ± 0.27	−3.210	0.985	2.05
		R.1404	CTTGACGTTCTGCCTCTTCC					
ATF3	XM_015099373.1	F.498	CCTCGAGATGTCAGTCACCA	(3)	25.2 ± 0.65	−3.832	0.987	1.82
		R.673	CCTTCAGCTCAGCATTCACA					
GPAM	XM_004020197.2	F.2297	ACCAGCAGTTCATCACCTTC	(4)	20.2 ± 0.24	−3.816	0.976	1.83
		R.2466	GTACACGGCAACCCTCCTCT					
GPAT4	XM_004021798.3	F.1383	ACTTCCATTACATCAGCCTGAGGC	(5)	23.8 ± 0.22	−3.439	0.999	1.95
		R.1483	CGTGAAAGCGAGAGCTATCCTG					
KLF6	XM_012.188639.2	F.281	GCAGCATCTTCCAGGAACTC	(3)	26.2 ± 0.29	−3.071	0.999	2.12
		R.449	AGATCTTCCTGGCTGTCGAA					
LPIN1	NM_001280700.1	F.2041	TGGCCACCAGAATAAAGCATG	(6)	26.2 ± 0.61	−3.418	0.998	1.96
		R.2141	GCTGACGCTGGACAACAGG					
PPT1	XM_004001836.2	F.744	CCCTGTGGATTCTGAGTGGT	(3)	24.5 ± 0.20	−3.178	0.994	2.06
		R.932	TGGGCGTAAAACCATTCTTC					
SCD	FJ513370.1	F.1100	GATGACATCTATGACCCAACTTACCA	(1)	15.9 ± 0.22	−3.351	0.996	1.99
		R.1200	CCCAAGTGTAACAGACCCATGA					
SREBF1	GU206528	F.152	GGGACAAGGTTTGCTCACATG	(1)	24.8 ± 0.45	−3.468	0.997	1.94
		R.267	GGCAGCTTGTCAGTGTCCACTA					
EIF3K	XM_004015230.2	F.368	CCAGGCCCACCAAGAAGAA	(7)	20.8 ± 0.08	−3.467	0.999	1.94
		R.492	TTATACCTTCCAGGAGGTCCATGT					
PPIA	NM_001308578.1	F.176	GGATTTATGTGTCCAGGGTGGTGA	(8)	18.0 ± 0.15	−3.552	0.997	1.91
		R.295	CAAGATGCCAGGACCTGTATG					
UXT	XM_004022128.3	F.417	TGTGGCCCTTGGATATGGTT	(9)	24.6 ± 0.10	−3.500	0.997	1.93
		R.517	GGTTGTCGCTGAGCTCTGTG					

[1]Primer direction (F – forward; R – reverse) and hybridization position on the sequence. The PCR annealing temperature was 60°C in all cases, except in *PPT1*, which was 62ºC.
[2](1) Bichi et al. (2013); (2) Toral et al. (in press); (3) This article; (4) Faulconnier et al. (2011); (5) adapted from Hussein et al. (2013); (6) Bionaz and Loor (2008); (7) Kadegowda et al. (2009); (8) Bonnet et al. (2000); (9) Bionaz and Loor (2007).
[3]Quantification cycle.
[4]Slope of the calibration curve.
[5]Coefficient of determination of the calibration curve.
[6]Calculated as $[10^{(-1 / \text{slope})}]$.

REFERENCES

Bichi, E. *et al.* Dietary marine algae and its influence on tissue gene network expression during milk fat depression in dairy ewes. *Anim. Feed Sci. Technol.* **186,** 36–44 (2013).

Bionaz, M. & Loor, J. J. Identification of reference genes for quantitative real-time PCR in the bovine mammary gland during the lactation cycle. *Physiol. Genomics* **29,** 312–319 (2007).

Bionaz, M. & Loor, J. J. ACSL1, AGPAT6, FABP3, LPIN1, and SLC27A6 are the most abundant isoforms in bovine mammary tissue and their expression is affected by stage of lactation. *J. Nutr.* **138,** 1019–24 (2008).

Bonnet, M., Bernard, L., Bes, S. & Leroux, C. Selection of reference genes for quantitative real-time PCR normalisation in adipose tissue, muscle, liver and mammary gland from ruminants. *animal* **7,** 1344–1353 (2013).

Bonnet, M. *et al.* Lipoprotein lipase activity and mRNA are up-regulated by refeeding in adipose tissue and cardiac muscle of sheep. *J. Nutr.* **130,** 749–56 (2000).

Faulconnier, Y., Chilliard, Y., Torbati, M. B. M. & Leroux, C. The transcriptomic profiles of adipose tissues are modified by feed deprivation in lactating goats. *Comp. Biochem. Physiol. Part D Genomics Proteomics* **6,** 139–149 (2011).

Hussein, M., Harvatine, K. H., Weerasinghe, W. M. P. B., Sinclair, L. A. & Bauman, D. E. Conjugated linoleic acid-induced milk fat depression in lactating ewes is accompanied by reduced expression of mammary genes involved in lipid synthesis. *J. Dairy Sci.* **96,** 3825–34 (2013).

Kadegowda, A. K. G. *et al.* Identification of internal control genes for quantitative polymerase chain reaction in mammary tissue of lactating cows receiving lipid supplements. *J. Dairy Sci.* **92,** 2007–2019 (2009).

Toral, P. G., Hervás, G., Belenguer, A., Carreño, D. & Frutos, P. mRNA abundance of genes involved in mammary lipogenesis during fish oil- or trans-10 cis-12 CLA-induced milk fat depression in dairy ewes. *J. Dairy Sci.* (*in press*). http://doi.org/10.3168/jds.2016-11820
